# Intrinsic organization of the corpus callosum

**DOI:** 10.3389/fphys.2024.1393000

**Published:** 2024-07-01

**Authors:** Paolo Barbaresi, Mara Fabri, Teresa Lorenzi, Andrea Sagrati, Manrico Morroni

**Affiliations:** ^1^ Department of Experimental and Clinical Medicine, Section of Neuroscience and Cell Biology, Marche Polytechnic University, Ancona, Italy; ^2^ Department of Life and Environmental Sciences, Marche Polytechnic University, Ancona, Italy; ^3^ Electron Microscopy Unit, Azienda Ospedaliero-Universitaria, Ancona, Italy

**Keywords:** nNOS, NADPHd, NK1, aspartate, glutamate, Glu_Rs_, Glut_Ts_, vGLU_Ts_

## Abstract

The corpus callosum—the largest commissural fiber system connecting the two cerebral hemispheres—is considered essential for bilateral sensory integration and higher cognitive functions. Most studies exploring the corpus callosum have examined either the anatomical, physiological, and neurochemical organization of callosal projections or the functional and/or behavioral aspects of the callosal connections after complete/partial callosotomy or callosal lesion. There are no works that address the intrinsic organization of the corpus callosum. We review the existing information on the activities that take place in the commissure in three sections: I) the topographical and neurochemical organization of the intracallosal fibers, II) the role of glia in the corpus callosum, and III) the role of the intracallosal neurons.

## 1 The corpus callosum

### 1.1 From images to function. A brief historical overview

**FIGURE 1:**
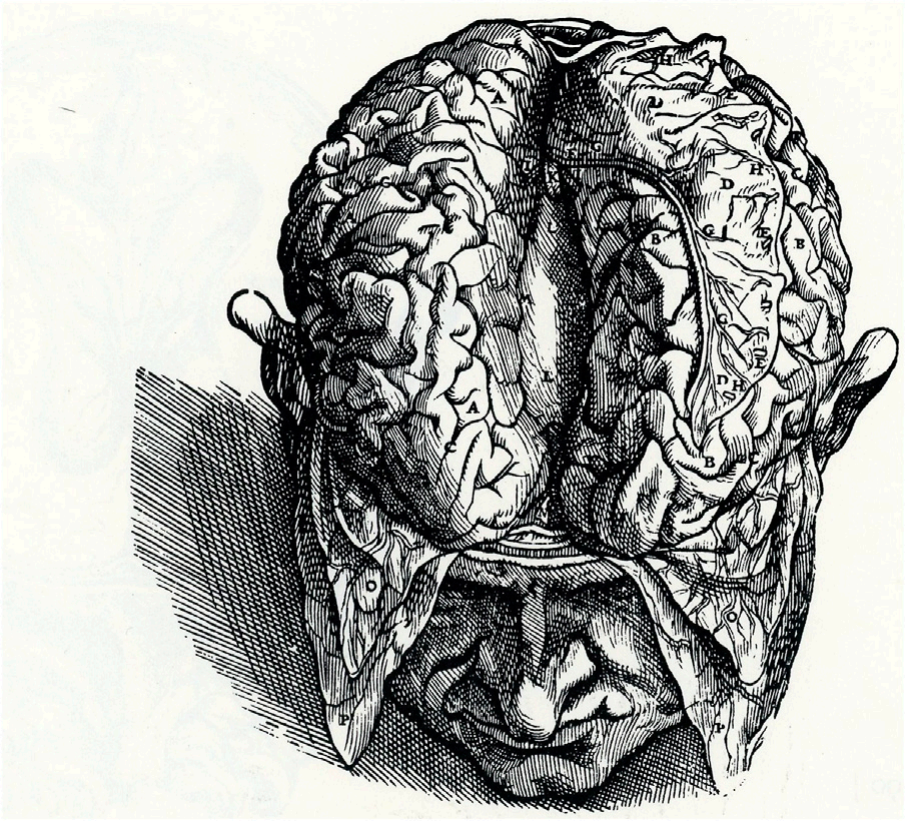
Dorsal view of the human brain. Separation of the two hemispheres exposes the dorsal portion of the CC. Modified from Plate 67:2 (VII: Figure 3), De Humani Corporis Fabrica.

The corpus callosum (CC) is by far the largest neuronal pathway connecting the two cerebral hemispheres (Innocenti, 1986). The earliest images of the CC are probably those drawn by Andreas Vesalius (Andries Wytinck van Wesel, 1514–1564), the greatest anatomist and dissector of the first half of the 16th century who published the first atlas of human anatomy (*De Humani Corporis Fabrica*). The last volume included an extensive and accurate depiction of the brain and its blood vessels and a representation of the CC (Figure 1 VII; Figure 3; Saunders and O’Malley, 1982; Cambiaghi, 2017; Scatliff and Johnston, 2014), which he described as follows: “… *The portion of the brain on the right and on the left have been separated from one another manually so that the superior aspect of the corpus callosum presents itself for inspection*” (Saunders and O’Malley, 1982). However, Vesalius did not advance any new hypothesis on the function of the CC (Manzoni, 2011). More than 150 years after, the Italian physician and neuroanatomist Giovanni Maria Lancisi provided a detailed description of the CC in 1712.

Lancisi hypothesized that sensory stimuli transported by the nerves through a “nervous fluid” were conveyed to the CC, which he therefore considered the seat of the soul and of imagination, deliberation, and judgement (Manzoni, 1998; Di Ieva et al., 2007). The hypothesis held until the end of the 18th century. Then, Franz Joseph Gall, by dissecting alcohol-fixed brains (a new method introduced by Johann Christian Reil for nerve tissue), described a bundle of fibers connecting the two hemispheres (Manzoni, 2011). Even though Gall’s findings were not widely accepted, they did provide an important starting point for subsequent studies on callosal connections.

**FIGURE 2:**
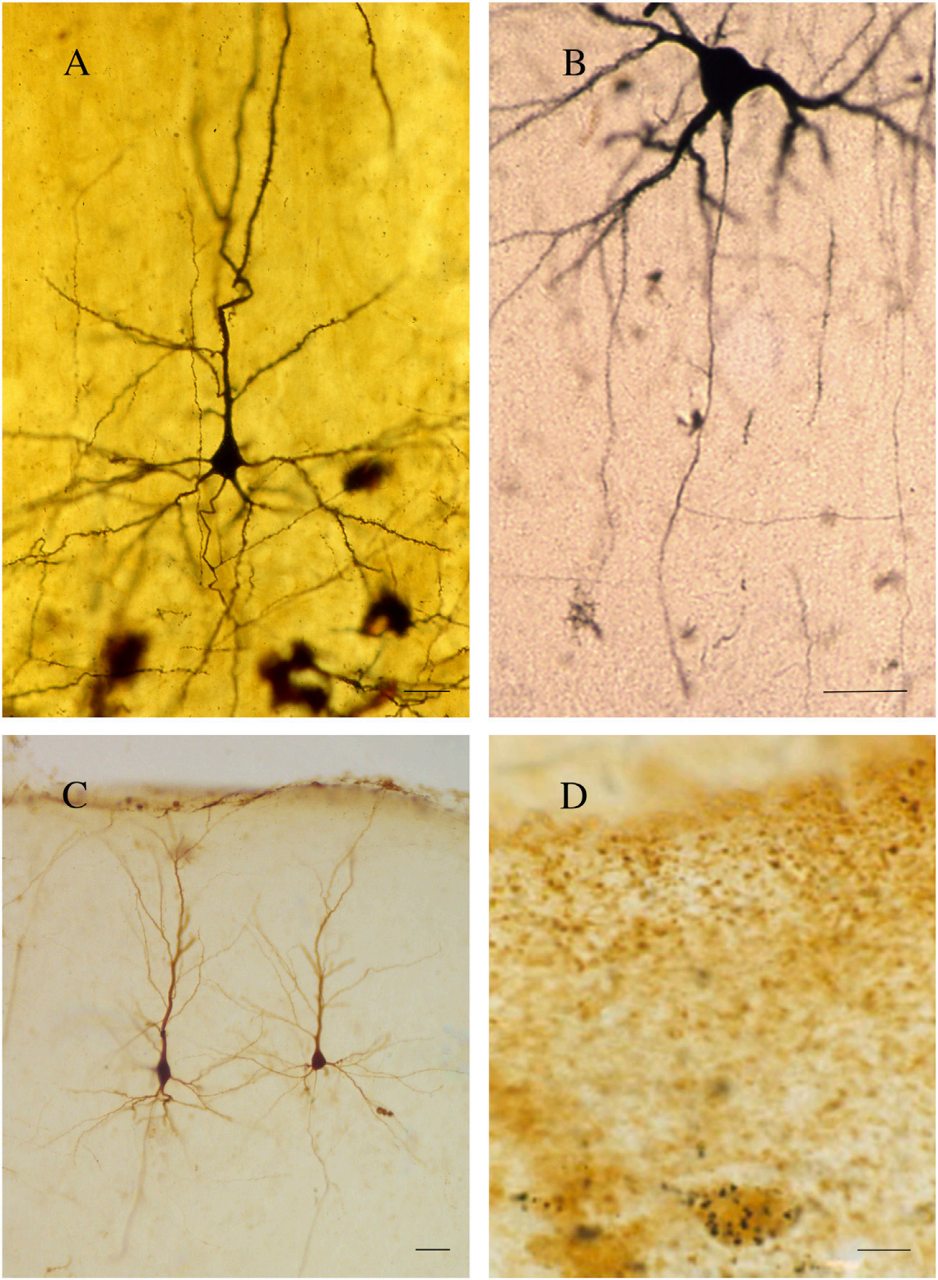
**(A)** A pyramidal neuron. Golgi technique (Golgi-Cox). Second somatosensory area (SII) of the cat. **(B)** A callosal neuron retrogradely labeled after the injection of HRP in the contralateral primary somatosensory area (SI). **(C)** Callosal neurons. Rat cerebral cortex; retrograde labeling with BDA (3 kDa) injected into contralateral SI. **(D)** Rat cerebral cortex; double-labeled neurons. GAD-positive neuron retrogradely labeled after the injection of WGAapoHRP-Au into contralateral SI. Calibration bars: 25 μm for **(A,B)**, 50 μm for **(C)**, and 10 μm for **(D)**. **(D) Figure 5** from Fabri and Manzoni (2004).

Further and more accurate information on callosal organization was obtained with the *reazione nera* (black reaction), a revolutionary silver chromate reaction devised by the Italian physician Camillo Golgi in 1873 (Figure 2A; Pannese, 1999). The Golgi method highlights nerve cells and their processes by covering them in silver salt chromate, which shows them in black against a yellow/orange background (Pannese, 1999). The technique was improved and widely used by Santiago Ramón y Cajal (de Castro et al., 2007), enabling him to advance his “neuron theory” (Cimino, 1999; Pannese, 1999).

**FIGURE 3:**
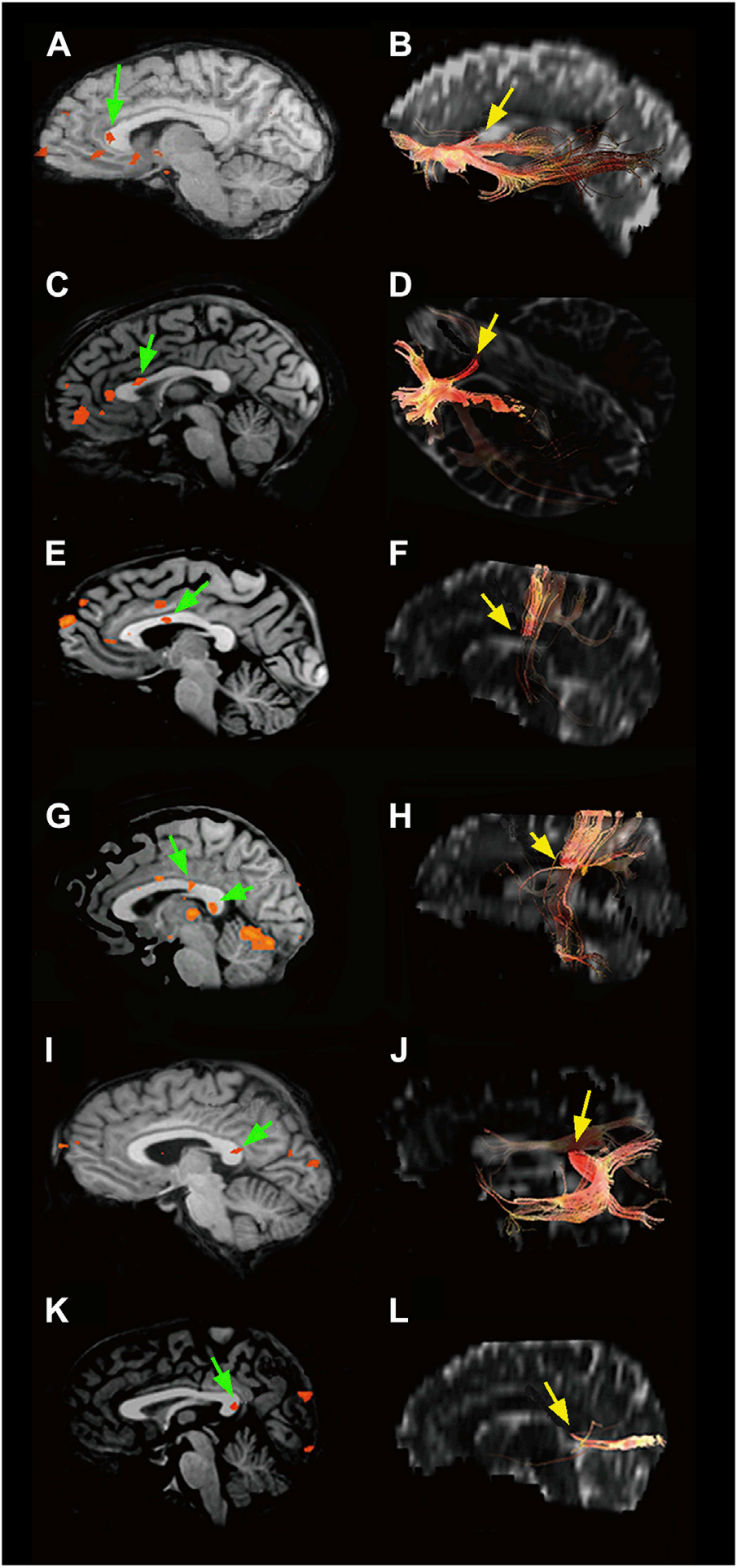
Left: BOLD effect evoked in the CC by different types of peripheral sensory stimulation (green arrows): **(A)** olfactory, **(C)** gustatory, **(E)** motor task, **(G)** tactile, **(I)** auditory, and **(K)** visual. Right: CC sites where fibers connecting activated cortical areas cross the commissure (yellow arrows): **(B)** olfactory cortices, **(D)** primary gustatory areas, **(F)** motor areas, **(H)** somatosensory cortices, **(J)** auditory areas, and **(L)** visual cortices. Modified from: Fabri et al. (2014).

For approximately 130 years, Golgi’s black reaction, though fairly capricious (Rosoklija et al., 2014), was the only technique capable of highlighting nerve cells in their entirety and was also used to confirm Ramón y Cajal’s results (Pannese, 1999; DeFelipe and Jones, 1988). Since the 1960s, it has been partially replaced by retrograde [horse radish peroxidase (HRP); Figure 2B, fluorescent dyes, biotinylated dextran amine (BDA 3 kDal; Figure 2C), and viral tracers] and anterograde (*Phaseolus vulgaris* leucoagglutinin, BDA 10 kDal, and tritiated amino acids) neuronal tracers. The new techniques combined with electrophysiological experiments have provided detailed information not only on the morphology (Manzoni et al., 1980; Tremblay et al., 1987; Soloway et al., 2002), location (Manzoni et al., 1986; Tremblay et al., 1987; Barbaresi et al., 1989; Barbaresi et al., 1994), and the size and complexity of the dendritic arbor and spine density (Soloway et al., 2002) of the neurons projecting throughout the CC but also on the axon terminal structure (Pandya and Rosene, 1993; Barbaresi et al., 1994) and dynamic interactions between the hemispheres (Innocenti, 1994). Subsequent experiments combining electron microscopy (EM) first with the Golgi stain (Cipolloni and Peters, 1983; Pannese, 1999; Fairén, 2005; Peters, 2007) and later with neuronal tracers and/or immunohistochemistry (IHC) allowed studying both the neurotransmitters used by the callosal cells/fibers (Figure 2D) and their ultrastructural organization (Barbaresi et al., 1994; White and Czeiger, 1991; Czeiger and White, 1997; Karayannis et al., 2007; Fairén, 2005). Recent magnetic resonance imaging (MRI)–associated techniques, i.e., functional MRI (fMRI), diffusion tensor imaging (DTI), and diffusion tensor tractography (DTT), provide powerful methods to investigate the human brain *in vivo* (Le Bihan and Karni, 1995). DTI supplies information about microstructural integrity of the CC by measuring fractional anisotropy, which positively correlates with conduction velocity, in turn depending upon myelination and/or axon diameter (Caminiti et al., 2013). DTI and DTT also allow the determination of the topography of the CC in greater detail (Chao et al., 2009). Notably, a combined DTI and fMRI approach can detect CC activation evoked by specific sensory or motor tasks, thus depicting its functional topographic organization (Fabri et al., 2014; Figure 3). Now, optogenetics—the most recent method to revolutionize neuroscience—allows introducing into a neuron population genes that encode light-sensitive channel proteins (opsins), which can then be selectively activated/inhibited through illumination (Scanziani and Häusser, 2009; Deisseroth, 2011). Activation/inhibition of discrete neuronal populations with different wavelengths has allowed the identification of a previously unknown, long-range inhibitory circuit and relating it to a genetically defined type of fibers, GABAergic neurons, involved in interhemispheric communication (Rock et al., 2018). Optogenetics has also enabled an extremely detailed investigation of the myelination process (Gibson et al., 2014).

The functions of the CC began to be understood in the early 20th century, through the study of disconnection syndromes (Barkovich, 1996). However, its key role in integrating the information reaching the two hemispheres was not uncovered until callosotomy was introduced in the 1940s, in an effort to interrupt the seizures of patients with intractable epilepsy (Gazzaniga, 2005). Since split-brain subjects did not show appreciable changes in behavior or intellectual abilities (Sperry, 1961; Gazzaniga, 2000; Glickstein and Berlucchi, 2008), Sperry and Gazzaniga devised specific tests to assess the functions of each hemisphere and the role of the CC by sending stimuli separately to each (disconnected) hemisphere (Gazzaniga, 1975; Gazzaniga, 2005). This is how they discovered the hemispheric lateralization of functions, i.e., the left hemisphere is dominant for speech, calculation, and planning movement, whereas the right hemisphere is dominant for visuospatial construction, mental rotation, and spatial matching (Gazzaniga, 2000; Gazzaniga, 2005).

## 2 Neuronal and non-neuronal elements that make up the CC

The CC consists of neuronal elements: callosal fibers (CFs), i.e., myelinated and unmyelinated axons, intracallosal neurons (ICNs), and non-neuronal elements such as the glial cells (GCs): oligodendrocytes, astrocytes, and microglia.

### 2.1 Neuronal elements

#### 2.1.1 Callosal fibers: topography

The CC is the most prominent commissure in placental mammals. It is the largest commissure of white matter in the human brain, with a cross-sectional area of 500–600 mm^2^ (Griffiths et al., 2009), a thickness ranging from approximately 0.5 cm to slightly more than 1 cm (the splenium is commonly the thickest region), and approximately 200 million axons (Aboitiz et al., 1992). Anatomical studies conducted in several animal species by injecting HRP into various cortical areas have documented that callosal axons (CAxs) mainly originate from the cerebral cortex pyramidal neurons in layers II/III and V and to a lesser extent in layer VI (Conti and Manzoni, 1994; Innocenti, 1986; Jacobson and Trojanowski, 1974; Manzoni et al., 1980; Karayannis et al., 2007; Tremblay et al., 1987; Segraves and Rosenquist, 1982; Shatz, 1977; Caminiti et al., 1979; Jones, 1984). Various types of non-pyramidal neurons—a heterogeneous population of cortical cells that include bipolar and multipolar neurons (Code and Winer, 1985; Innocenti, 1986; Tremblay et al., 1987; Hughes and Peters, 1990; Hughes and Peters, 1992; Conti and Manzoni, 1994; Martínez-García, et al., 1994)—also contribute to CC formation.

From the anterior to posterior, five CC regions have been identified by anatomical studies (Goldstein et al., 2022): the rostrum, hooked around the anterior commissure; the genu, curving gently around the lower edge of the frontal lobe; the trunk or body (subdivided into anterior, middle, and posterior), which constitutes most of the visible callosal portion; the isthmus, a narrow area located between the posterior body and the anterior portion of the splenium, where the fornix joins the CC; and the splenium, a thick swelling resting on the quadrigeminal lamina, which marks the posterior edge of the CC. Each of these regions is traversed by fibers that connect mainly homotopic, but also heterotopic, regions of the two hemispheres. The rostrum is crossed by fibers connecting the orbital regions of the frontal lobes. The fibers of the genu contribute to form the forceps minor and connect the prefrontal cortices. The body of the CC, together with other white mater (WM) pathways, participates in the formation of the corona radiata. In the anterior callosal region, most fibers connect homologous premotor (supplementary motor areas) and motor areas, whereas in the posterior CC, a smaller number of fibers connect the somatosensory and parietal cortices. The isthmus carries the commissural fibers of the pre- and postcentral gyri (motor and somatosensory strips) and of the primary auditory cortex, whereas fibers passing through the splenium give rise to the forceps major and connect the occipital lobes (Aboitiz et al., 1992; Aboitiz and Montiel, 2003; Hofer and Frahm, 2006; Hofer et al., 2008; Caminiti et al., 2009; Raybaud, 2010). Similar findings have been described by an *in vivo* MRI study using DTI, which has also documented a similar callosal topography in humans and other primates (Hofer et al., 2008; Fabri et al., 2014). By applying recent MRI-associated techniques, such as fMRI, DTI, and DTT, to callosotomized patients (Fabri et al., 1999; Fabri et al., 2001; Fabri et al., 2005), it has been demonstrated that touch interhemispheric transfer is accomplished by axons crossing the posterior CC. The examination of other sensory modalities has provided evidence that transfer of visual (Gazzaniga and Freedman, 1973; Clarke et al., 2000) and auditory information (Sugishita et al., 1995; Pollmann et al., 2002) between the hemispheres takes place in the splenium (Fabri et al., 2014). Furthermore, a previous article (Mesulam, 1998) has hypothesized that brain regions are organized according to a gradient ranging from low-order functions involving primary cortical areas (e.g., sensory–motor) to higher-order functions involving transmodal processes (e.g., default mode network), following a hierarchical organization of cognitive functions. This gradient, spanning from perception and action to abstract cognitive functions, is known as the principal gradient and is presumably the same on the two hemispheres. Later, Friedrich et al. (2020) proposed to map the principal gradient onto the CC, the largest commissure of white matter in humans and considered to be mainly responsible for the interhemispheric integration. In his approach, a new parcellation modality of the CC was suggested on the basis of functional organization. Specifically, the anterior part (anterior body, genu, and rostrum) was found associated with high values of the principal gradient, i.e., with high-order-function cortical areas, and the posterior part (posterior body, isthmus, and splenium) was associated with low values of the principal gradient, i.e., with low-order-function cortical areas (Friedrich et al., 2020).

The topography of CFs has also been investigated in cats by the application of HRP into the CC. CFs from the ventral half of the frontal cortex cross through the rostrum, whereas those from the ventral, occipital, and dorsal temporal cortices pass through the ventral splenium (Nakamura and Kanaseki, 1989). More accurate topographical information on the CC in cats has been provided by injecting the bidirectional tracer WGA-HRP (Lanciego and Wouterlood, 2006) into different cytoarchitectonic areas (Matsunami et al., 1994). Injections into areas 4 and 6 (precruciate motor areas) labeled CFs that cross the genu, whereas injections into area 3a (postcruciate somatosensory cortex) stained commissural fibers passing through the posterior genu. Labeled fibers from the rostral lateral gyrus (LG; area 5) pass through the rostral body, whereas fibers from the posterior LG (visual cortex; area 17) pass through the splenium. Fibers coming from the cingulate gyrus follow a rostrocaudal direction, as injections into areas 24 and 23 labeled commissural fibers in the rostral and caudal bodies, respectively. A study of the rat splenium involving WGA-HRP injections into the posterior cerebral cortex has found a fiber organization mirroring the rostrocaudal topography of the cortex. As regards its topographical organization, the more anterior fibers proceed from the anterior 17/18a border and those from the middle and posterior 17/18a borders occupy the middle and posterior portions of the splenium. The most posterior fibers originate from both the 18b border and temporal cortex. Notably, the axons from these cortical regions overlap in the rat splenium (Kim et al., 1996). Light microscopy (LM) and EM studies performed in humans and in non-human primates have disclosed that the CC is chiefly composed of myelinated axons (LaMantia and Rakic, 1990; Aboitiz et al., 1992; Caminiti et al., 2009), whose size shows wide differences along the anteroposterior axis, with thin fibers in the anterior region of the CC (genu) and thicker fibers mainly in the middle and posterior poles of the CC (splenium). Indeed, the genu of the monkey CC contains the highest density of thin fibers, with diameters ranging from 0.4 to 0.6 μm. The axons are thicker in the body (midbody/posterior midbody 1.04–1.13 μm) and smaller in the posterior body (0.82 μm) and anterior part of the splenium (0.77 μm), whereas those crossing through the splenium are again thicker, measuring 0.95 μm (Caminiti et al., 2013). Histological and MRI studies have further confirmed this distribution in the human CC (Aboitiz et al., 1992; Caminiti et al., 2013). The largest commissural fibers (3–5 μm) are found in the human isthmus, midbody, and splenium and show a peak density in the posterior midbody (Aboitiz et al., 1992).

Unmyelinated fibers (UFs) are scarce. The diameter of the few UFs range from 0.1 to 1 μm. In the human CC, quantitative analysis has shown that UFs are more numerous in the genu, where they account for 16% of all fibers, whereas in the other callosal regions, they are fewer than 5% (Aboitiz et al., 1992). In non-human primates, UFs account for less than 10% of all CFs (LaMantia and Rakic, 1990; Caminiti et al., 2009). In mammals such as rodents, felines, and canids, UFs account for more than 30% (Olivares et al., 2001); in particular, they range between 41.6% and 56.1% in adult cats (Koppel and Innocenti, 1983) and 46.35% in rats (Seggie and Berry, 1972). Moreover, in the rat splenium, UFs outnumber myelinated axons by approximately a factor of 7 to 1 (Kim et al., 1996), whereas in lagomorphs, they comprise approximately 45% of the fiber population of the visual CAxs, whose diameter ranges from 0.08 to 0.6 μm (Waxman and Swadlow, 1976). These studies assume a uniform distribution of myelinated tracts throughout the axon length. However, an EM serial reconstruction study has found that some axons of pyramidal neurons of different neocortical layers give rise to CC fibers that exhibit intermittent myelination (Tomassy et al., 2014). Altogether, the studies reviewed above suggest that the CC of humans and non-human primates is a heterogeneous fiber tract, whose rostrocaudal and dorsoventral compositions differ according to the topographical organization of the cortex. In the diverse callosal portions, fibers of different sizes with a different degree of myelination have different conduction velocities. In particular, thin, poorly myelinated, slow-conducting fibers are numerous in the genu and rostral splenium, which connect prefrontal and temporoparietal association areas, whereas CFs crossing through the posterior body, isthmus, and posterior splenium, which connect motor, somatosensory, auditory, and visual areas, are larger, highly myelinated, and fast conducting. The largest CFs could be involved in phasic influence on their contralateral projection field, whereas the small amyelinic axons subserve the slow conduction of information between the two hemispheres, playing a tonic influence or exercising a modulatory effect on the excitation or inhibition produced by faster myelinated fibers (LaMantia and Rakic, 1990; Aboitiz et al., 1992; Innocenti et al., 2022).

Regional differences in CF size as marked in those reported in primates have not been described in the other analyzed mammals. Moreover, CFs show a rough and fairly diffuse topographical arrangement, with an overlap at least in the splenium (LaMantia and Rakic, 1990; Aboitiz et al., 1992; Kim et al., 1996; Aboitiz and Montiel, 2003; Barazany et al., 2009; Caminiti et al., 2009).

#### 2.1.2 Neurotransmitters

##### 2.1.2.1 Glutamate

**FIGURE 4:**
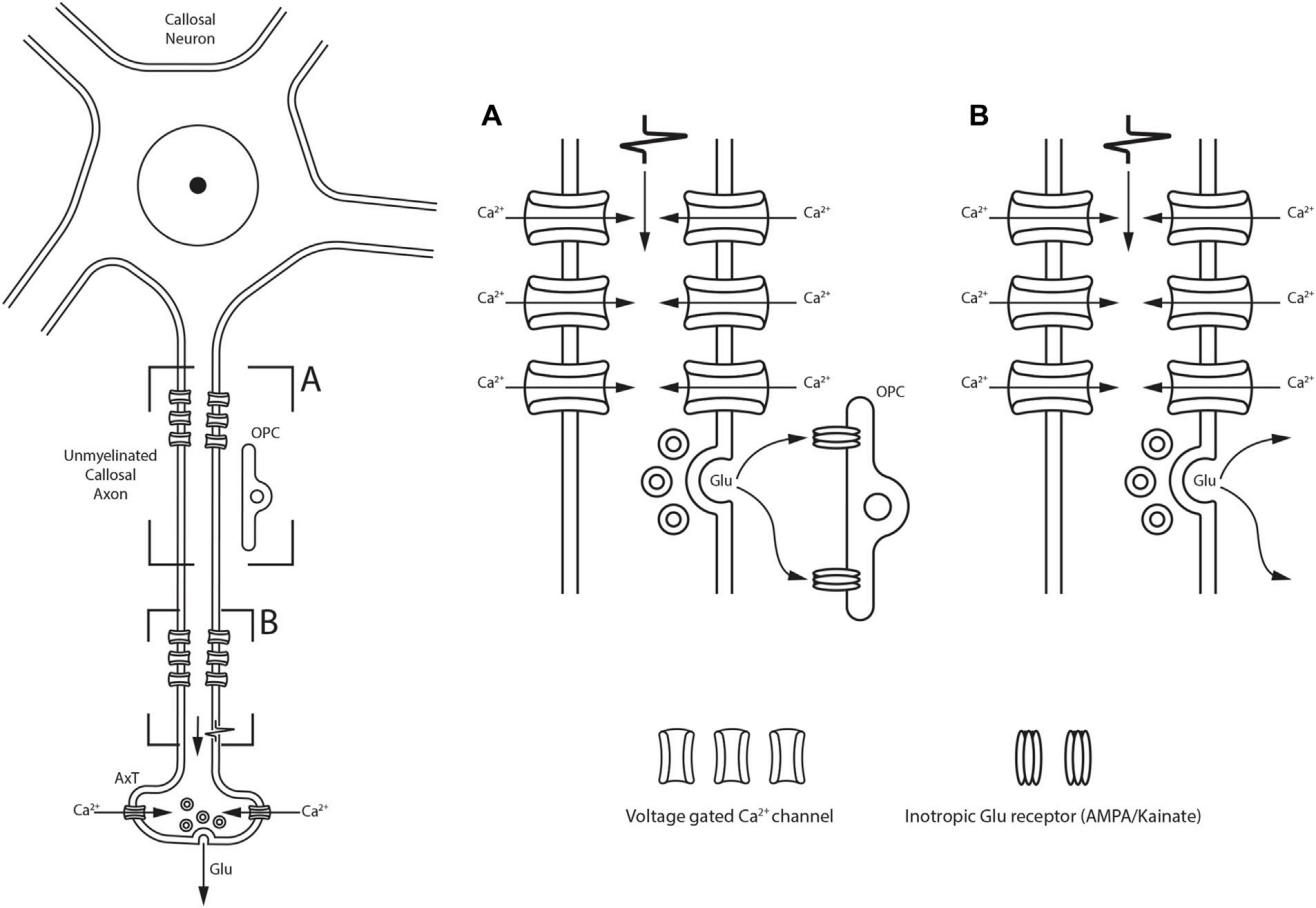
Schematic drawing showing two different modalities of Glu release along the unmyelinated fibers within the CC depending on the synaptic vesicle location. **(A)** synaptic vesicles cluster close to the axon membrane located just in front of OPC; Glu release is detected by ionotropic Glu receptors located on OPC. **(B)** Glu is also released at discrete sites along axons in the callosal parenchyma, not necessarily directly on OPC membranes.

The vast majority of CFs use the excitatory glutamate (Glu) and/or aspartate (Asp) as neurotransmitters (Conti and Manzoni, 1994). Neurotransmitter action is regulated by proteins found on cell and synaptic vesicle membranes: Glu receptors (Glu_Rs_), Glu transporters (Glut_Ts_), and vesicular Glut_Ts_ (vGlu_Ts_). Experiments involving the removal of the cerebral cortex in rats induced a reduction in Asp uptake (Fonnum et al., 1981) and a significant decrease in Glu levels in the contralateral hemisphere (Peinado and Mora, 1986). Furthermore, electrical stimulation of the cortical regions projecting to the lateral suprasylvian area enhanced the release of excitatory amino acids (Hicks et al., 1985). Based on the notion that only cerebral cortex neurons with a high-affinity uptake mechanism for a given neurotransmitter would be retrogradely labeled after injecting such a neurotransmitter (Streit, 1984; Barbaresi et al., 1985; Barbaresi et al., 1987; Elberger, 1989), tritiated metabolic inert D-[^3^H]-Asp (an analog radioactive metabolically inert molecule that is taken up by the same affinity mechanism as L-Asp and L-Glu) was injected into the first (SI) and/or second (SII) cat somatosensory areas of one hemisphere (Barbaresi et al., 1987) or in cat and rat visual areas (Elberger, 1989). The injections induced not only retrograde labeling of several callosal neurons but also intense labeling of several CFs found respectively in the rostral body (see Figures 7A,B of Barbaresi et al., 1987) and the splenium (Elberger, 1989). The distribution, morphology, and proportions of Glu- and Asp-containing neurons that give rise to callosal projections have been explored in the rat, cat, and monkey cerebral cortex by combining retrograde transport of tracers like HRP and/or WGA-HRP with IHC using antisera directed against Glu or Asp (Conti et al., 1988a; Conti et al., 1988b; Dinopoulos et al., 1989; Giuffrida and Rustioni, 1989; Dori et al., 1992). In the rat visual cortex, 38% of the callosal neurons were Glu-positive (Glu ^+^) and 49% were Asp^+^ (Dinopoulos et al., 1989; Dori et al., 1992); similar proportions were found in the motor and somatosensory areas (Giuffrida and Rustioni, 1989). Counts were slightly different in the cat somatosensory areas, where retrogradely labeled Glu^+^ neurons were 40%–50%, whereas callosal Asp^+^ neurons were 55%–57% (Conti et al., 1988a). In the monkey somatosensory areas, Glu^+^ callosal neurons accounted for 61%–68% of the projecting neurons (Conti et al., 1988b). Glu^+^ and Asp^+^ callosal neurons were pyramidal and found throughout layers II–VI (Conti et al., 1988a; Conti et al., 1988b; Dinopoulos et al., 1989; Giuffrida and Rustioni, 1989; Dori et al., 1992). Electrophysiological and neurochemical studies have suggested that Glu is released in the CC (Kukley et al., 2007; Ziskin et al., 2007). In rat and mouse CC, the propagation of action potentials (APs) along the axons leads to rapid vesicular release of Glu in an unusual manner (Figure 4). Voltage-gated Ca^2+^ channels (Ca_Vs_), similar to those of nerve terminals, are found along the axons of unmyelinated CFs. In response to receiving the AP, these proteins mediate an intra-axonal Ca^2+^ increase that induces vesicle fusion with the presynaptic axon membrane, triggering fast vesicle release of Glu (Kukley et al., 2007). Glu release from axons occurs in two different ways, depending on the synaptic vesicle location: i) up to 40 synaptic vesicles cluster close to the axon membrane located just in front of the thin glial processes (NG2^+^, see below)—here, vesicle fusion occurs at the axon–glia interface and Glu release activates Glu_Rs_ on the postsynaptic glial membrane (Figure 4A; Gallo et al., 2008; Kukley et al., 2007); ii) small clusters of vesicles and fusion protein are scattered along an unmyelinated axon and do not interface with the glial processes—here, Glu is released at discrete but arbitrary sites, not necessarily on the glial cell membrane surface (Figure 4B; Kukley et al., 2007; Ziskin et al., 2007). In the latter case, Glu is released by axon fibers in a diffuse manner and can interact with neighboring axons or with ICN membranes (Jovanov-Miloševic’ et al., 2010; see below). Glu release from axons that traverse the CC is enhanced by repetitive stimulation of callosal neurons and can be inhibited by the activation of metabotropic Glu receptors (mGlu_Rs_) autoreceptors (Ziskin et al., 2007). Moreover, CAxs can implement rapid vesicle-filling mechanisms. Brief exposure of tissue sections containing the CC to hyperosmolar sucrose and FM1-43 (a styryl fluorescent dye widely used to visualize secretory vesicle recycling) induced FM1-43 uptake and fluorescent labeling of numerous CFs that have internalized vesicles that take up FM1-43. Perfusion of slices with high K^+^ concentration resulted in a dramatic decrease in fluorescence due to FM1-43 exocytosis. Destaining of putative axon fascicles was prevented by pharmacological blockade of Ca^2+^ entry. These findings suggest that CAxs are capable of highly dynamic exo-endocytotic recycling of Glu-filled vesicles (Kukley et al., 2007). These data are further supported by the presence of vGlu_Ts_ on CFs. Detection of discrete vGlu_T1_^+^ puncta in axons throughout the CC suggests an intense refilling activity in CF vesicles. Confocal microscopic analysis has documented that vGlu_T1_^+^ puncta are often closely related to NG2^+^ processes, whereas electron micrograph observations have shown vGlu_T1_ immunoreactivity in axons forming synaptic junctions (Ziskin et al., 2007).

##### 2.1.2.2 γ-Amino butyric acid

The rat and cat CC contains a small proportion of GABAergic fibers. According to anatomical studies combining retrograde tracers (e.g., WGA-HRP, CTB-Au, or WGAapoHRP-Au) with antibodies for glutamic acid decarboxylase (GAD; Fabri and Manzoni, 2004), the enzyme involved in γ-amino butyric acid (GABA) synthesis (Gonchar et al., 1995), GABAergic callosal neurons in rat and cat somatosensory areas account for 0.6%–1% of all retrogradely labeled cells. Different (and conflicting) results have been reported in the mouse CC. Here, the injection of fast blue (FB), a retrograde fluorescent tracer, into several cortical areas of a GAD67–green fluorescent protein (GFP) knock-in mouse found extremely rare double-labeled GFP-FB neurons, which characterize GABAergic transcallosal fibers (Tomioka et al., 2005). By contrast, studies using viral tracing and optogenetic stimulation have found numerous parvalbumin-expressing (Parv^+^) axons in mouse CC (Rock et al., 2018; Zurita et al., 2018). All Parv^+^ neurons also show intense GABA staining and constitute a large population of GABAergic neurons (Gonchar et al., 2008); notably, 42% of the entire population of Parv^+^ neurons in the mouse auditory cortex give rise to a dense GABAergic-Parv^+^ callosal projection (Rock et al., 2018).

##### 2.1.2.3 Adenosine

Combined electrophysiological and immunocytochemical studies have suggested that CF axonal conduction may be modulated by neurotransmitters. Notably, adenosine A1 receptor (A1_R_) has been described in rat CAxs by using immunocytochemistry (ICC). The lipid-soluble adenosine agonist, cyclopentyladenosine (CPA), acting at A1_Rs_ alters axon physiology by enhancing K^+^ conductance, thus reducing the amplitude of callosal APs. CPA also induces a reduction in the number of neurotransmitters released (Swanson et al., 1998). The myelin (lipid) barrier masks the A1_Rs_, which cannot be reached by water-soluble compounds like adenosine (hydrophilic). Therefore, axonal A1_Rs_ can only respond to adenosine released from CAxs (Swanson et al., 1998).

##### 2.1.2.4 Acetylcholinesterase

Acetylcholinesterase-positive (AChE^+^) CFs with a largely dorsoventral orientation, i.e., perpendicular to the long axis of the CC, have been described in adult monkeys by using ICC (Rockland and Nayyar, 2012). In macaque monkeys, these fibers may originate from pyramidal neurons of layers III and V of the cerebral cortex, which give rise to callosal connections and express strong AChE reactivity (Mrzljak and Goldman-Rakic, 1992).

### 2.2 Intracallosal neurons

#### 2.2.1 ICNs in the adult CC

**FIGURE 5:**
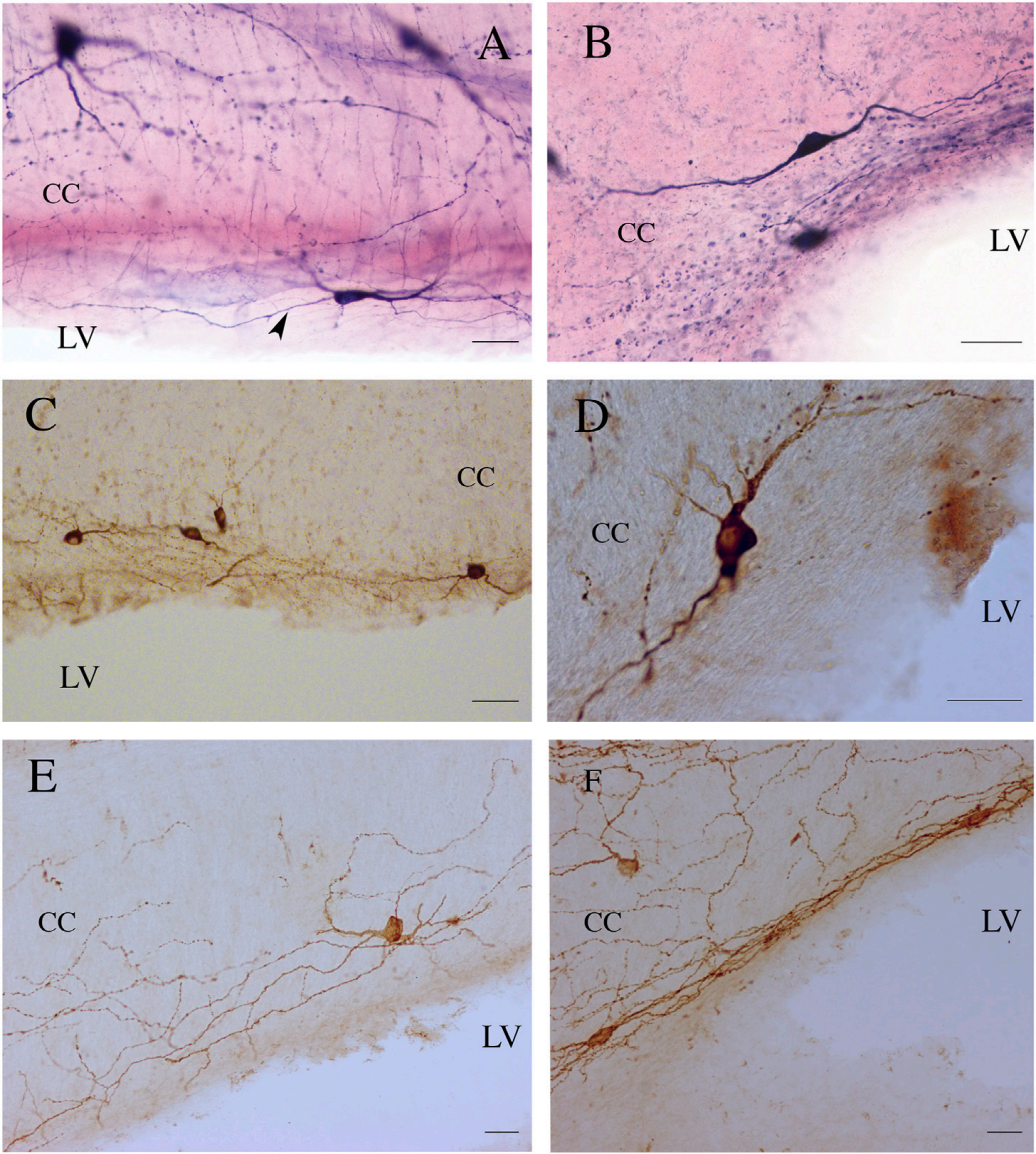
ICNs, fibers, and puncta positive for NADPHd **(A,B)**, nNOS **(C,D)**, and NK1 **(E,F)** in the ependyma of rat CC. Arrowhead in **(A)** indicates a supposed axon emerging from the base of NADPHd ICN. Calibration bars: 25 μm for **(A,B,D,E)**, 50 μm for **(C)**.

**FIGURE 6:**
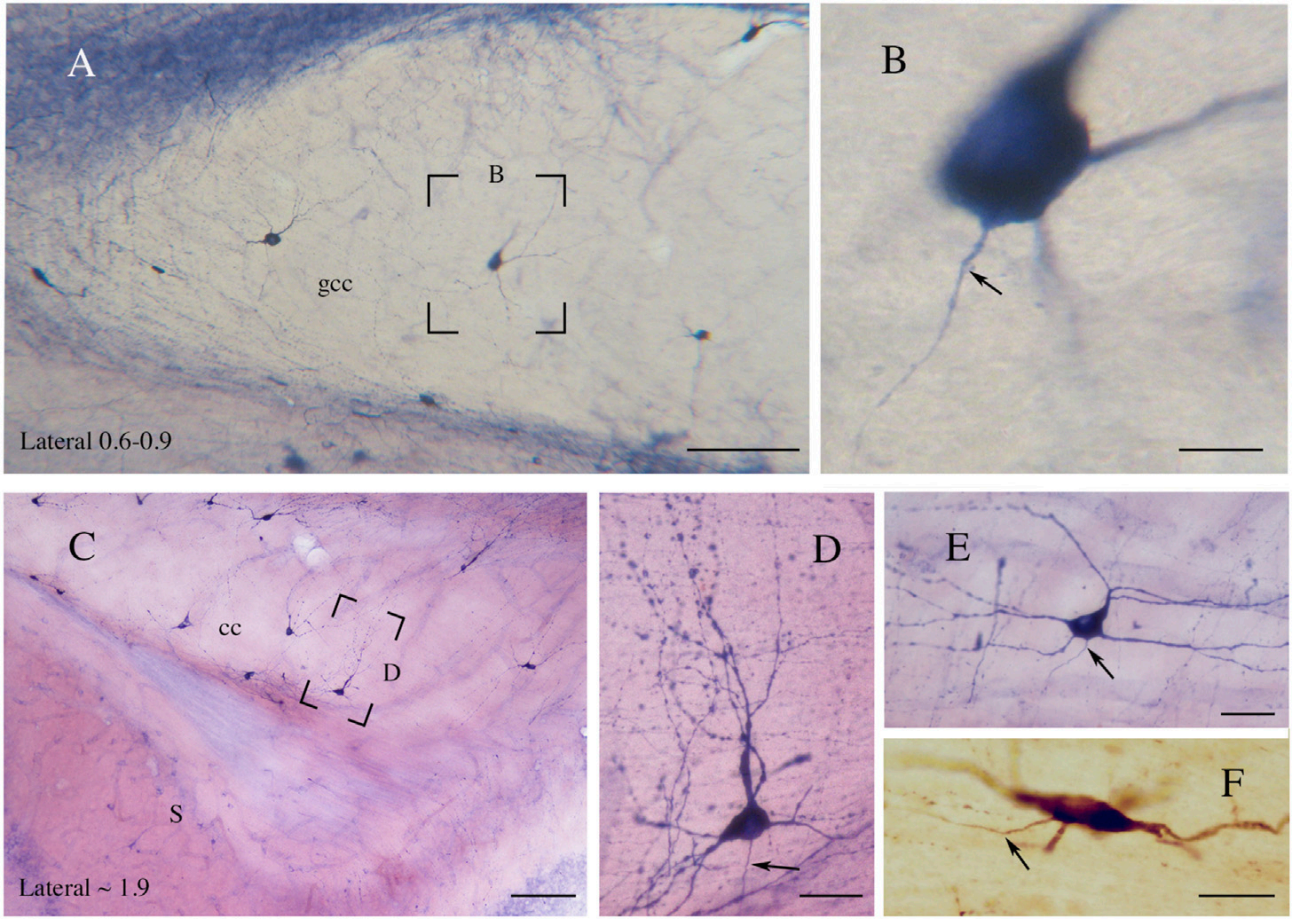
**(A)** Genu of the rat CC showing NADPHd-ICNs. The framed area, enlarged in **(B)**, shows an NADPHd-ICN; an axon emerges from the base of the cell body. **(C)** An NADPHd-ICN in the splenium of rat CC. Framed area enlarged in **(D)**. **(D)** An NADPHd-ICN showing a wide dendritic tree with several varicosities. **(E)** An NADPHd-ICN in the body of rat CC; a triangular ICN showing a wide dendritic field. **(F)** An nNOS-immunopositive ICN in the body of rat CC. Arrows in **(B,D,E,F)** indicate axons. Stereotaxic levels according to the atlas of Paxinos and Watson (1982). S, subiculum. Calibration bars: 250 μm for **(A,C)**, 10 μm for **(B,F)**, 25 μm for **(D,E)**. **(E)** modified from Barbaresi et al., 2020.

**FIGURE 7:**
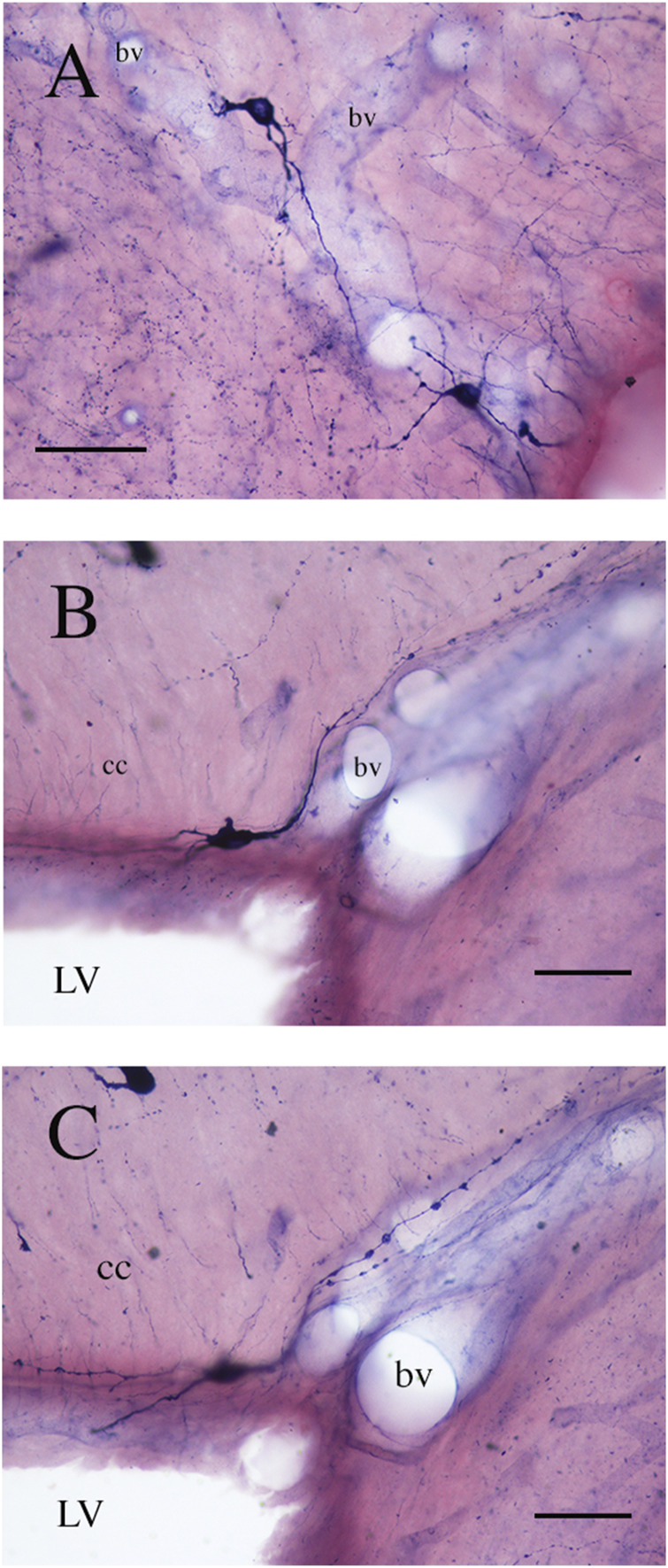
Photomicrographs showing NADPHd-ICNs lying close to blood vessels (bv). **(A)** The soma and dendrites of NADPHd-ICNs are closely apposed to blood vessels, which are also contacted by stained fibers and a spray-like cluster of NADPHd-positive puncta. **(B)** and **(C)** show the same photographic field from two different focal planes. Dendrites show several varicosities wrapped around blood vessels. cc, corpus callosum. Calibration bars: 50 μm for **(A)**, 25 μm for **(B)**.

**FIGURE 8:**
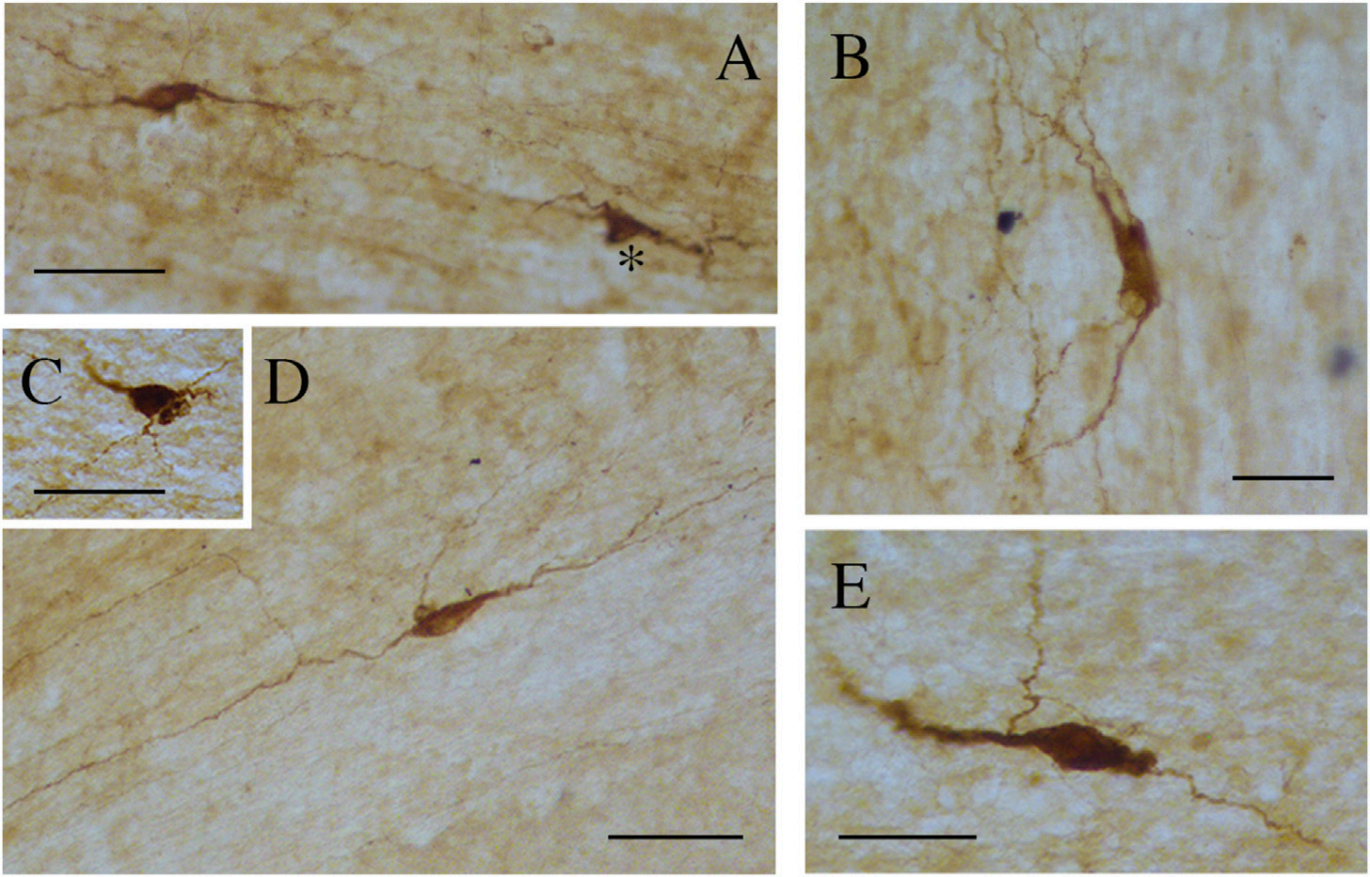
Photomicrographs showing different nNOS-ICN morphologies in human CC. **(A)** A fusiform and a triangular (asterisk) ICN, **(B)** a rectangular ICN, **(C)** a round ICN, **(D,E)** fusiform ICNs. Calibration bars: 50 μm.

**FIGURE 9:**
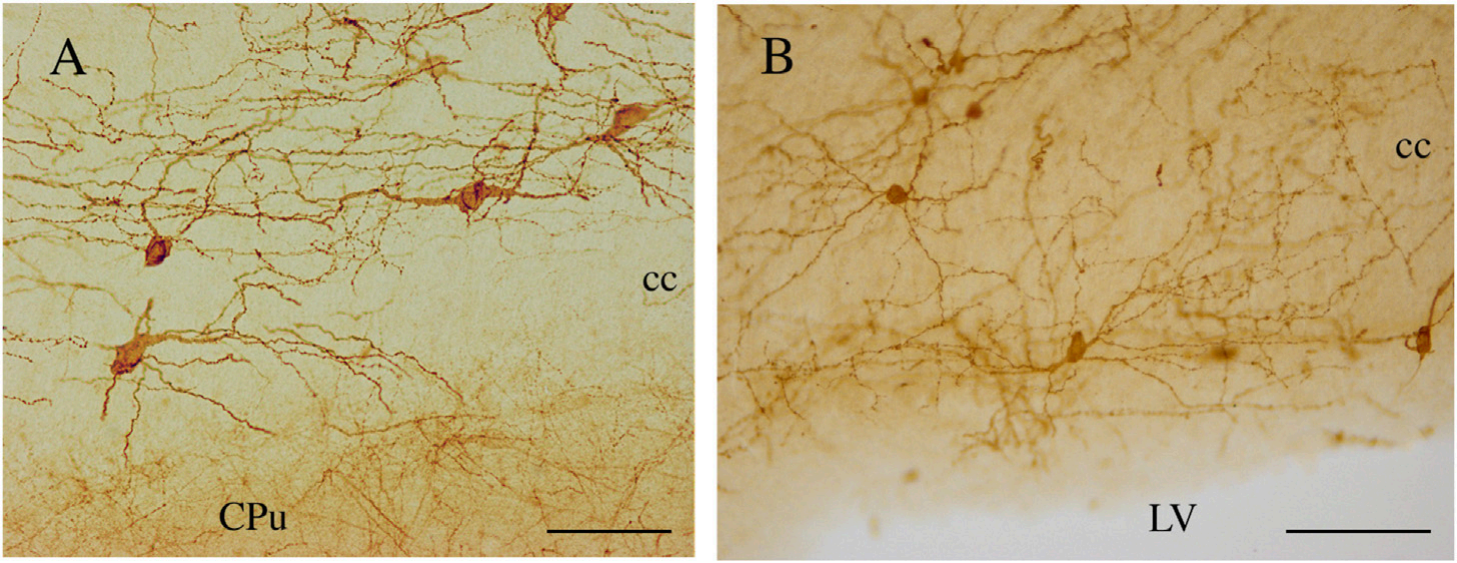
NK1-ICNs [P30 in **(A)**; P20 in **(B)**] forming an intricate dendritic network in the central and ependymal regions of the rat CC. CPu, caudate putamen; cc, corpus callosum. Calibration bars: 50 μm in **(A)**; 100 μm in **(B)**.

**FIGURE 10:**
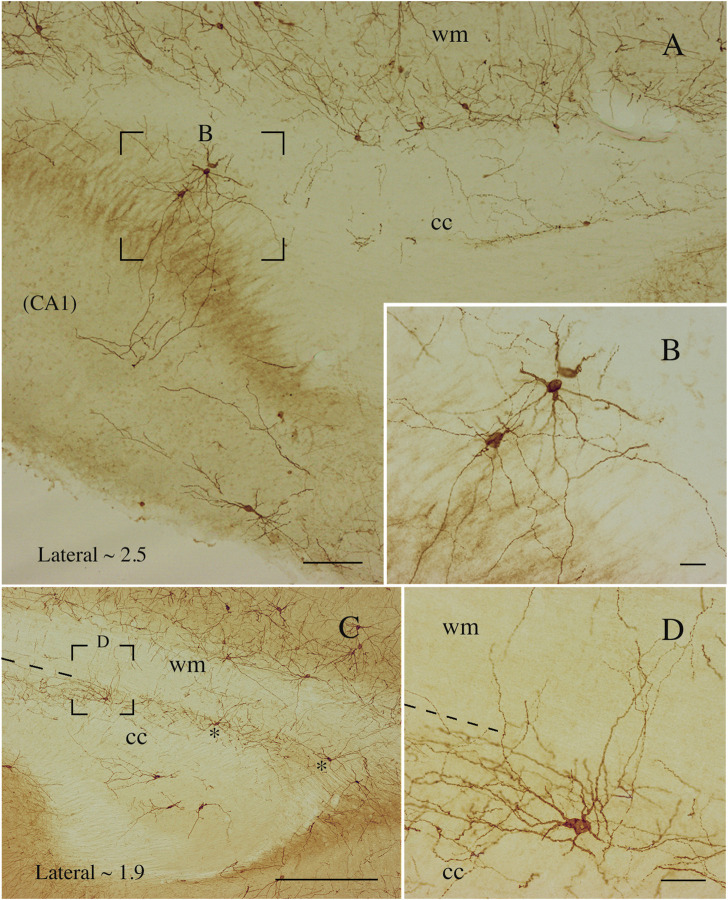
Photomicrographs taken at different lateromedial levels showing NK1-ICNs [lateral ∼2.5 in **(A)**; lateral ∼1.9 in **(C)**]. **(A)** NK1-ICNs sending dendrites into the underlying hippocampus. The framed area in **(A)** is magnified in **(B)** showing NK1-ICNs in the dorsal border of the CC sending dendrites toward the overlying WM. **(C)** Two neurons are marked by asterisks and another, in the framed area, is shown at a higher magnification in **(D)**. **(D)** An ICN showing a wide dendritic arborization reaching the overlying WM. cc, corpus callosum; wm, white mater. Calibration bars: 250 μm for **(A)**, 500 μm for **(C)**, 25 μm for **(B,D)**.

**FIGURE 11:**
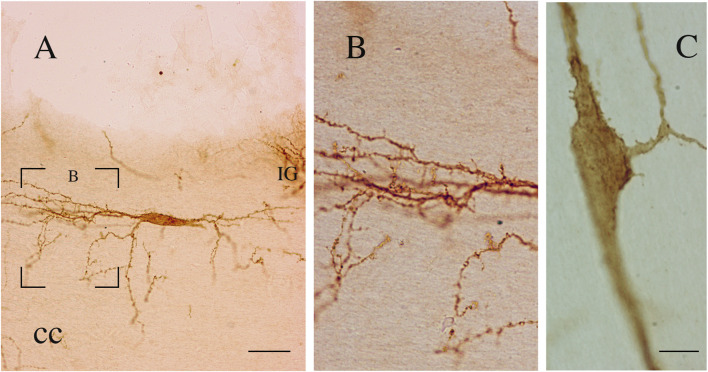
**(A–C)** Two bipolar NK1-immunopositive ICNs (coronal sections). The neuron in **(A)** (P15) shows a wide dendritic field. The framed area, enlarged in **(B)**, shows several dendritic appendages and dendritic spines. **(C)** NK1-ICN (P20) showing somatic and dendritic appendages and dendritic spines. cc, corpus callosum. Calibration bar; IG, indusium griseum. 20 μm in **(A)** and 10 μm in **(C)** B is modified from Barbaresi et al., 2017.

Besides GCs, the CC also contains “intracallosal neurons” (Jovanov-Miloševic’ et al., 2010) (Table 1). In 1984, Malobabic and co-workers were the first to describe two types of neurons in the human CC using Golgi stain. One type had fusiform/ovoid somata with few, long, and slightly ramified dendrites perpendicular to the CFs, while the other type consisted of multipolar neurons with dendrites radiating in all directions, either perpendicular or parallel to the CFs. Most of the neurons that were more frequently impregnated were detected in callosal regions adjacent to the indusium griseum (IG) or in the callosal sulcus, while the others were mostly located in the central and ventral regions (Malobabic et al., 1984). Subsequent ICC studies have provided further information on the intrinsic organization of ICNs. In the cat CC, Riederer et al. (1992, 2004) described ICNs expressing microtubule-associated protein 2 (MAP2), one of the microtubule-associated proteins of the vertebrate nervous system (Nunez, 1988; Goedert et al., 1991). MAP2 consists of at least three isoforms produced from a single gene: two high-molecular-weight isoforms: MAP2a and MAP2b and a low-molecular-mass isoform: MAP2c (Nunez, 1988; Goedert et al., 1991). MAP2b-containing cells were detected in the ventral area of the rostrum (Riederer et al., 2004). Their neuronal nature was demonstrated using double labeling with the glycolic enzyme: neuron-specific enolase (NSE). All MAP2^+^ cells were also positive for NSE (Riederer et al., 2004). MAP2^+^ ICNs showed different morphologies. Some were pyramidal with triangular perikarya and wide dendritic trees, whereas others had spindle-shaped somata with primary dendrites arising from the two poles (Riederer et al., 2004). ICNs expressing the calcium-binding proteins calretinin (CalR) and calbindin (CalB) were also identified by using ICC. Labeled spindle-shaped cell bodies were found on the upper and lower CC borders. Two poorly developed primary dendrites emerged from their somata (Riederer et al., 2004). CalR^+^ ICNs have also been described in mice (Revishchin et al., 2010). They were not numerous and were detected ventral to the cingulum and sometimes also in the dorsal wall of the lateral ventricle (LV). They had small (8–10 μm), round perikarya giving rise to one or, occasionally, two dendrites bearing spine-like processes of different shapes, defined as polymorphous spines (Revishchin et al., 2010). Nitrous oxide (NO)–producing ICNs have been described in several species including humans (Sancesario et al., 1996; Rockalnd and Nayyar, 2012; Barbaresi et al., 2014; Barbaresi et al., 2015; Barbaresi et al., 2020; Sagrati et al., 2023). NO is a small gaseous molecule involved in numerous biological functions in the mammalian nervous system (Bredt et al., 1990; Iadecola, 1993; Garthwaite and Boulton, 1995; Knott and Bossy-Wetzel, 2009; Förstermann and Sessa, 2012). NO-producing neurons were demonstrated by using ICC, by the localization of neuronal NO synthase (Barbaresi et al., 2014), an enzyme that synthesizes NO from L-arginine (Schmidt et al., 1988). Several investigations have addressed NOS distribution in the central nervous system (CNS) using nicotinamide adenine dinucleotide phosphate diaphorase histochemistry (NADPHd-Hi). NADPHd is an NOS and is therefore considered to be a specific marker of NO-producing neurons (Dawson et al., 1991; Hope et al., 1991). Multipolar NO-producing ICNs have been demonstrated in the rat CC and/or in subcortical WM using NADPHd-Hi (Sancesario et al., 1996; Barbaresi et al., 2014), with documented varicose dendrites extending toward the roof of the LV (Figures 5A,B). Some NADPH^+^ ICNs exhibit long axons (Figures 5A, 6), either running parallel to the CFs or curving toward the overlying cerebral cortex or the LV (Figure 5A). Using NADPHd-Hi and NOS-ICC, Barbaresi et al. (2014) described numerous NO-producing neurons in rat CC. NADPHd neurons (NADPHd^+^Ns) and neuronal nitric oxide synthase neurons (nNOS^+^Ns) exhibit the same morphology and distribution, are densely stained, and have a Golgi-like appearance (Figures 5A–D, 6). They are classified into four types by their soma and dendrite characteristics: bipolar (fusiform and rectangular, 28.03% of labeled ICNs); round (19.26%); polygonal (quadrangular, 30.11%); and pyramidal (triangular–pyriform, 22.58%; Table 2). The dendrites of NO-producing ICNs branch in all directions and, depending on their location in the CC, are able to reach different CNS regions (Figure 10). Those found in the genu sent their dendrites to the overlying WM, layer VI of the cerebral cortex, the IG, or the underlying caudate–putamen nucleus. Dendrites of ICNs lying in the anterior callosal body run toward the cerebral cortex, the IG, and/or the ependymal surface, while those in the middle and posterior body, isthmus, and splenium extend inferiorly toward the hippocampal alveolus or the WM (Barbaresi et al., 2014; Barbaresi et al., 2020). The dendrites of all morphological types show varicosities and/or protrusions resembling dendritic spines. Very thin axons originating from the soma or, less frequently, from the base of the proximal dendrites can be followed for several tens of microns (Figures 5A, 6). Labeled neurons are abundant along the rostrocaudal dimension of the rat CC but show regional lateromedial variation. In fact, NADPHd^+^Ns/nNOS^+^Ns are numerous in the lateral regions and progressively diminish in the medial CC, where they are rare or absent (Barbaresi et al., 2014). The vast majority of labeled ICNs (87.7%–89.1%) is in the callosal body, whereas those located in the ependymal region, close to the LV, account for 8.89%–12.70%. Ependymal ICNs are predominantly fusiform (Figures 5A,B; Barbaresi et al., 2014). Labeled ICNs, single or in clusters, are frequently closely associated with blood vessels (Figure 7), which are often completely surrounded by a dense network of nerve fibers and puncta (Barbaresi et al., 2014). ICN processes form a dense network both in the body of the CC and in the ependymal regions (Figure 5). Networks of labeled neurites in the ependymal layer are probably in contact with the cerebrospinal fluid (CSF) and are formed by fibers coming from neurons in the callosal body and from the ICNs in the roof of the LV (Sancesario et al., 1996; Barbaresi et al., 2014; Figure 5). NO-producing neurons are also detected in adult macaque monkeys (Rockland and Nayyar, 2012). However, since the latter study involved only the rostral (rostrum and genu) and medial CC, comparisons of the regional distribution of NO-producing neurons between rodents and primates are not feasible. Sparse NADPHd^+^Ns have been detected in monkey CC parenchyma. NADPHd^+^Ns are also identified along the dorsal and ventral CC margins. The most common ICN type in these regions are multipolar neurons followed by bitufted and bipolar cells. The sparse distribution of the NADPHd^+^Ns does not allow the formation of a dense network of nerve fibers as described in the rat CC. Dendrites of bipolar and multipolar ICNs are closely associated with blood vessels. Fine processes, probably axons, are often detected. No data are available on the presence of neurons in the ependymal regions of the monkey CC (Rockland and Nayyar, 2012). In the human CC, ICNs are generally scattered throughout the rostrocaudal dimension and show a mediolateral gradient, being more numerous in the lateral CC. The body contains more NO-producing ICNs than the other callosal regions. Clusters of neurons also lie at the boundary with the IG. All human NO-producing ICNs are bipolar, showing an ovoid, a round, or a fusiform morphology (Figure 8), and are often closely associated with the blood vessels (Sagrati et al., 2023). AChE^+^ neurons and fibers have occasionally been described in the monkey CC. They usually display a dorsoventral orientation perpendicular to the long axis of the CC (Rockland and Nayyar, 2012). The rat CC also contains neurons expressing neurokinin 1 receptor (NK1_R_) (Figures 5E,F,9; Barbaresi et al., 2015; Barbaresi et al., 2017), the receptor with the highest affinity for substance P (SP; Harrison and Geppetti, 2001). The distribution of NK1^+^Ns is similar to that of nNOS^+^Ns. NK1^+^Ns have been described along the rostrocaudal extension of the rat CC, and along the lateromedial dimension, they increase from the lateral to medial and decrease near the stereotaxic zero point. NK1^+^Ns are less numerous than nNOS^+^Ns at all stereotaxic levels. NK1^+^Ns show a wide dendritic field (Figures 5E,F,9,10), which, based on the location of the cell body in the CC, reaches different brain areas (Figure 10, see above nNOS^+^Ns). Dendrites often bear spines or fine dendritic processes (Figure 11; Barbaresi et al., 2015; Barbaresi et al., 2017). Confocal microscopy examination demonstrates that nearly all NK1^+^Ns (96.43%) contained nNOS and that 84.59% of nNOS^+^Ns co-express NK1. These data suggest that most ICNs release NO as a result of the action of SP. A small proportion of nNOS^+^Ns, which does not contain NK1 and is not activated by SP, might release NO via alternative mechanisms (Barbaresi et al., 2014). The sparse ICNs described in adult humans show poor labeling. One to three neuronal nuclear antigen–expressing (NeuN^+^) neurons *per* section were detected in the rostrum and/or genu of subjects aged 42–59 years. Other antibodies for neuron markers like neuropeptide Y (NPY), MAP2, AChE, and the calcium-binding proteins (CalR and CalB) highlight ICNs only in early postnatal development or during gestation (Jovanov-Miloševic et al., 2010; see below). By contrast, CalB^+^ ICNs were still present and well developed in a 6-year-old child (Jovanov-Miloševic’ et al., 2010). However, in the adult CC, ICNs may be more numerous than highlighted by ICC. In fact, “*the Golgi method impregnates only about 1% to 5% of actually present neurons, and the fact that we found some of them in every (oblique) section, implies that their number is considerable*” (Malobabic et al., 1984). These differences may be due to methodological issues, such as sensitivity of the ICC method, or the small number of NeuN^+^ ICNs in adults.

**TABLE 1:** Classification of ICNs and their location and possible function.

Intracallosal neurons	Species	Technique	Location in the CC	Possible role	References
NO ICNs	Rats	Hi, ICC	Permanent population. Numerous in the lateral CC and gradually decreases in the more medial regions	Composition of ventricular CSF; regulation of CC blood flow; BOLD effect; myelinogenesis; retraction of exuberant and transient CC axons; stabilization of axons with appropriate connections	Sancesario et al. (1996), Barbaresi et al. (2014), Barbaresi et al. (2015), Barbaresi et al. (2020)
NO ICNs	Monkeys	Hi	Permanent population. Sparsely distributed over the anterior two-thirds of the CC, predominantly in the rostrum and genu. The splenium was not examined	Axon guidance; myelination; regulation of callosal blood flow; involvement in widespread network organization	Rockland and Nayyar (2012)
NO ICNs	Humans	ICC	Permanent population found at the upper boundary of the CC according to a mediolateral gradient. More numerous in the lateral callosal regions	Study of callosal neurovascular regulation and BOLD effect	Sagrati et al. (2023)
AChE ICNs	Monkeys	ICC	Data not available for the early postnatal period. Present in the adult CC. Scattered in the genu and rostrum. Dorsoventral orientation	Possible interaction between NO-producing neurons and AChE neurons to control callosal microcirculation. Ach is not found in the CC	Rockland and Nayyar (2012)
AChE ICNs	Humans	Hi	Transient neuronal population, present in the mid-fetal period up to the 29th week post-conception. Rare in the adult CC	Possible interaction of NO-producing neurons and AChE ICNs in control of the callosal microcirculation. Ach is absent in the CC	Jovanov-Miloševic et al. (2010)
MAP2 ICNs	Postnatal and adult cats	ICC	Partially transient ICNs. Numerous at birth. In the adult, a small contingent of MAP2^+^ ICNs are found in the ventral area of the rostrum	Axon stabilization, axon guidance, and dendritic maturation	Riederer and Innocenti (1992), Riederer et al. (2004)
MAP2 ICNs	Humans	ICC	Double-labeling experiments with NeuN. Transient ICNs. Same distribution as NeuN	Axon stabilization, axon guidance, and dendrite maturation	Jovanov-Miloševic et al. (2010)
Peptidergic ICNs NPY ICNs	Rats	ICC	Present in the rostral third of the rat CC (see Figure 1; **Figure 2** in Chronwall et al. (1985). Rare in the adult	Local release of NPY by ICNs could produce a very localized vasoconstriction. NPY ICNS in combination with NO ICNs could participate in callosal blood flow regulation, probably in early development stages	Ding and Elberger (2000), Chronwall et al. (1985), Woodhams et al. (1985)
NPY ICNs	Humans	ICC	Transient population present in infants and children. Morphology changes with age. Rare in the adult CC	NPY ICNs together with NO ICNs could participate in the regulation of callosal blood flow	Jovanov-Miloševic et al. (2010)
Calcium-binding protein (CBP) ICNs Calretinin (CalR) Calbindin (CalB)	Rats. Mice	ICC	Upper and lower border of the CC (both CBPs). Ventral to the cingulum and in the dorsal wall of the LV	CalR, unknown. Probably involved in neurogenesis processes	Riederer, et al. (2004), Revishchin et al. (2010)
CalR-CalB ICNs	Humans	ICC	Transient populations. Neurons were detected in the dorsal and ventral callosal surfaces. CalR-positive cells disappeared after the third postnatal month. CalB-positive cells were still detected in the CC of a 6-year-old child	The early appearance of CalR and CalB immunoreactivity in human CC suggests a yet unknown role for them, possibly in neurite elongation and remodeling, during postnatal development	Jovanov-Miloševic et al. (2010)
Neuron-specific nuclear protein (NeuN)	Humans	ICC	In combination with MAP2. Transient neuronal population distributed throughout the rostrocaudal CC. In the mid-prenatal period, they were numerous in the ventral CC; in the postnatal period, they were located dorsally, bordering the IG	The physiological function of NeuN is unknown	Jovanov-Miloševic et al. (2010)
NeuN	Humans	ICC	In combination with nNOS; same distribution as NO ICNs	The physiological function of NeuN is unknown	Sagrati et al. (2023)
NK1_R_ ICNs	Rats	ICC	NK1 ICNs; double-labeled ICNs: NK1/nNOS-NK1 ICNs show the same distribution as NO ICNs	NK1-ICNs increased between P5 and P10, then declined, but unlike other ICNs, NK1-ICNs make up a significant population in the adult CC through the activation of NK1_Rs_ by SP; NO is released in the CC, the CSF, and other CNS areas	Barbaresi et al. (2015), Barbaresi et al. (2017), Barbaresi et al. (2020)

**TABLE 2:** Number and percentage of subtypes of ICNs-NADPHd+ neurons. Modified from Barbaresi et al. (2014).

Case	Bipolar fusiform rectangular, N (%)	Round, N (%)	Polygonal, N (%)	Pyramidal triangular pyriform, N (%)	Total
Case1	105 (22.3) 17 (3.62) 122 (26.01)	106 (22.6%)	142 (30.37)	37 (7.88) 62 (13.21) 99 (21.10)	469
Case2	147 (25) 26 (4.42) 173 (29.42)	106 (18.02	176 (29.93)	65 (11.05) 68 (11.56) 133 (22.61)	588
Case3	102 (26.08) 9 (2.30) 111 (28.38)	67 (17.13)	118 (30.17)	53 (13.55) 42 (10.74) 95 (24.29)	391
Total	354 (24.44) 52 (3.59) 406 (28.03)	279 (19.26)	436 (30.11)	155 (10.70) 172 (11.87) 327 (22.58)	1448

#### 2.2.2 Connections of ICNs

Their large dendritic arborization and length of axons allow ICNs to reach different CNS regions (Figures 5,9, 10). In a study (Engelhardt et al., 2011), ICNs bearing serotonin 5-HT_3A_ receptor and identified in transgenic mice by the expression of enhanced green fluorescence protein (EGFP) were filled with biocytin and subsequently reconstructed (Engelhardt et al., 2011). The three to five dendrites emerging from the soma of biocytin-filled ICNs were aspinous but often showed irregular varicosities. Depending on their position in the CC, they extend to the lower layers of the cingulate or retrosplenial cortex and simultaneously to the underlying hippocampus or striatum. Axons often originate from the base of the main dendrite and form an elaborate plexus on the postsynaptic pyramidal cell located in the overlying cingulate cortex. At other times, the axon crosses the midline, arborizing in the contralateral CC and cingulum (Engelhardt et al., 2011). Notably, patch-clamp recordings of connected pairs of EGFP-expressing ICNs and cortical cells have revealed that some cells are reciprocally connected. EGFP-expressing ICNs receive excitatory and inhibitory input from cortical and subcortical cells and some of them exert an inhibitory action on postsynaptic cortical neurons. In these cells, inhibitory postsynaptic potentials are completely blocked by gabazine, a GABA_AR_ antagonist, consistent with the GABAergic phenotype of EGFP-expressing ICNs (Engelhardt et al., 2011).

#### 2.2.3 Postnatal development of ICNs and fibers

##### 2.2.3.1 Postnatal development of ICNs

**FIGURE 12:**
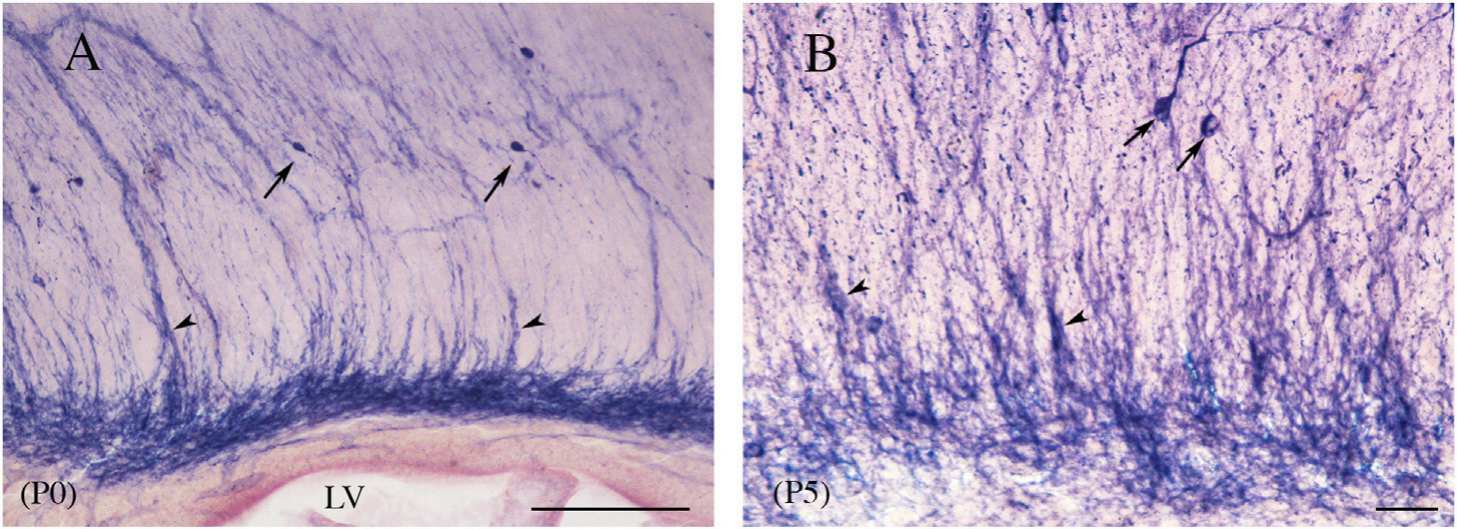
Sagittal view of rat CC at P0 **(A)** and P5 **(B)**. Streaks of different widths, interpreted as callosal septa (arrowheads), depart from dense bands of labeling lying at the base of the CC, reaching the dorsal callosal region. Arrows point to NADPHd-ICNs; arrowheads indicate to the callosal septa. Calibration bars: 100 μm for **(A)**, 50 μm for **(B)**.

An important feature of postnatal callosal development is transient ICNs and transient ICFs (Innocenti, 1986). A conspicuous cluster of GABA^+^ neurons coming from the rat medial and caudal ganglionic eminence (Niquille et al., 2013) has been described in the middle CC from late prenatal life to P0. GABA^+^ ICNs have also been detected at later postnatal ages, but they begin to decrease at P1 and disappear by P21 (DeDiego et al., 1994; Niquille et al., 2009; Niquille et al., 2013). Notably, transient GABAergic ICNs crossing through the CC have been reported to express the transcription factor Sp8. During the second postnatal week, these cells are densely packed, forming elongated chain-like aggregates traversing the CC (Cai et al., 2015). An immunocytochemical study has described transient GABA-immunopositive neurons in the mouse CC. They are numerous at post-natal day 0 (P0) and disappear over the first 3 postnatal weeks; between P5 and P8, several neurons show the morphology of degenerating cells (Del Rio et al., 1992). Another population of ICNs in the developing rat CC, NPY^+^ cells, increased up to P7 (Ding and Elberger, 2000) and then gradually decreased to a sparse presence in adults (Chronwall et al., 1985; Woodhams et al., 1985; Ding and Elberger, 2000). In adults, NPY^+^ ICNs were bipolar, vertically oriented, or multipolar, without a specific orientation, and were predominantly located in the rostral third (Chronwall et al., 1985). These ICNs send their axons through the CC (Ding and Elberger, 2000). NPY^+^ ICNs are regulated by neurotrophic factors such as brain-derived neurotrophic factor (BDNF), whose administration significantly increases the number of NPY-immunopositive neurons and fibers in P5–P6 rats (Yoshimura et al., 2009). These data implicate the BDNF in the development and maturation of NPY^+^ ICNs (Yoshimura et al., 2009). Other transient populations consist of glutamatergic ICNs expressing CalR, which disappear abruptly between P1 and P3, and of GAD67/Mash1-GFP^+^ GABAergic ICNs, which disappear progressively between P7 and P21 (Niquille et al., 2009). Migrating ICNs have been described in C57BL/6 mice. 1, 1′-Dioctadecyl-3,3,3′,3′-tetramethylindocarbocyanine perchlorate (DiI) crystals implanted in the mid-sagittal CC labeled cell bodies of pioneer neurons. These cells, which are found in the CC bundle, have bipolar and fusiform perikarya from which branch two short processes. A double-labeling ICC study using DiI and vimentin antibody, to exclude the glial nature of these cells, found no DiI^+^ cells labeled with vimentin (Deng and Elberger, 2001). Reelin, a 400-kDa glycoprotein, with an important role in the formation of laminated structures like the cerebral cortex, is expressed by numerous rat and mouse ICNs. Reelin-expressing ICNs are present in rat/mouse brain P7, P14, and P21 (Misaki et al., 2004). A double immunofluorescence study with NeuN antibody confirmed their neuronal nature, as all reelin-expressing cells were also NeuN^+^. These neurons are bipolar and project two slender cytoplasmic processes within the fascicles of the CC. All reelin^+^ ICNs are also GABAergic (Misaki et al., 2004). However, in the absence of any information on the number of ICNs found at the various postnatal ages or in adults, these data are difficult to evaluate and compare with previous studies. During postnatal development, MAP2-immunopositive neurons also appear transiently in the cat CC. They were ∼570 at birth and ∼200 in the adult (i.e., a 65% reduction). Their distribution changed during development since at P1–P11, they were detected throughout the anteroposterior dimension of the CC, whereas in the adult, they were concentrated in the ventral area of the rostrum (Riederer et al., 2004). Similar data have been reported for human ICNs (Jovanov-Milošsevic’ et al., 2010). ICNs labeled with neuronal markers (MAP2, NeuN, NPY, CalR, CalB) were more numerous and more morphologically complex at the end of the fetal period and diminished after the first postnatal year, with only 5%–10% of the initial population remaining in the adult (Jovanov-Miloševic’ et al., 2010). The postnatal trend of NO-producing neurons described in rat CC partially differs from that in previous studies (Riederer et al., 2004; Jovanov-Miloševic’ et al., 2010; Misaki et al., 2004; Ding and Elberger, 2000; Niquille et al., 2009; DeDiego et al., 1994). NADPHd^+^Ns have been detected already at P0, their numbers increase in the first few postnatal days, peaking at P5. Between P5 and P10, they begin to decline, although only by approximately 25%. Since their numbers remain constant up to P30, the adult CC contains as many as 2,000 NO-producing ICNs (Barbaresi et al., 2014; Barbaresi et al., 2020). The size of NADPHd^+^Ns increases in the first postnatal month, peaking between P0 and P15. From P5, cell bodies and dendrites are often associated with blood vessels. Furthermore, in the first five postnatal days, labeled striations of different widths, intermingled with NADPHd^+^Ns, are seen to run radially from the ventral to the dorsal CC (Figure 12; Barbaresi et al., 2020). These fibrous complexes, denominated “callosal septa” (Jovanov-Miloševic’ et al., 2006), divide the CC into irregular segments crossed by bundles of axon fibers and have also been described in the developing monkey and human CC (Killackey and Chalupa, 1986; Rakic and Zecevic, 2003).

A pattern similar to that reported for NO-producing ICNs has been described for NK1+Ns. These cells first appear at P5, their numbers then increase from P5 to P10 and decline up to P30. However, unlike other ICNs (see above; Jovanov-Miloševic’ et al., 2010; DeDiego et al., 1994; Niquille et al., 2009; Niquille et al., 2013; Cai et al., 2015; Ding and Elberger, 2000; Deng and Elberger, 2001; Yoshimura et al., 2009; Riederer et al., 2004), NK1+Ns are a significant population in the adult CC (Barbaresi et al., 2017). From P5 onward, their distribution is adult-like and similar to that of NO-producing neurons (see above). Their size increases constantly from P5 (102.3 μm^2^) to P30 (262.07 μm^2^) they stain densely; and their Golgi-like appearance allows a detailed morphological study. At P5, NK1+Ns have predominantly round and irregular cell bodies and thick primary dendrites with frequent varicose swellings. A more mature morphology of the perikaryon and a striking increase in dendrite complexity are seen at P10 (Figure 9B). Dendrites reaching both the ependymal layer and the overlying WM display many spines and short, thin appendages that are particularly numerous at P15 (Figure 11). Between P15 and P30, NK1+Ns are qualitatively indistinguishable from adult ICNs (Figures 9A,B) and can be classified as bipolar (fusiform and rectangular), round–polygonal, and pyramidal (triangular–pyriform). Their dendrites can be followed into the overlying WM and the CC ependymal region, where several NK1^+^Ns are also detected, for hundreds of microns. Dendrites bear several swellings and some spines, though fewer than they do at P15 (Figure 11C; Barbaresi et al., 2017).

##### 2.2.3.2 Postnatal development of ICFs

The presence of different contingents of transient axonal bundles is well established in the developing CC. In the perinatal rat CC, a GABA antiserum applied to label axon-like processes disconnected from their parent cell bodies has demonstrated GABA-immunoreactive (ir) fibers mostly grouped into longitudinally and/or vertically oriented bundles that sometimes span the entire dorsoventral CC (Cobas et al., 1988). Isolated transverse axons crossing the midline, predominantly located in the dorsal region of the CC, are also observed. Axons are particularly abundant at P0, but progressively decrease during postnatal development so that at P6, only a narrow fiber bundle is still visible through the whole thickness of the CC (Cobas et al., 1988). A similar distribution has been described in adult mice, where sparse GABA-ir fibers seem to be axons of transient transcallosal cortical cells (Ottersen and Storm-Mathisen, 1984). The origin of these fibers has been investigated in rat pups (P0–P1) by combining retrograde labeling with ICC and electrophysiology (Kimura and Baugham, 1997). Transcallosal GABAergic cells identified by double labeling (ICC plus retrograde labeling) have accounted for 21% of the whole callosal population, whereas those identified by electrophysiology are 57%. The discrepancy is probably due to the different experimental approaches. The number of transcallosal GABAergic cells plummeted later in development, reaching 0.6%–1% in the adult rat/cat (Gonchar et al., 1995; Fabri and Manzoni, 2004). In the postnatal mouse, transcallosal GABAergic cells have been reported to exert a monosynaptic inhibitory action, mediated by GABA_ARs_, on postsynaptic cortical neurons and glia (Berger et al., 1992b; Kimura and Baugham, 1997). The developing rat CC also contains transient peptidergic ICFs. NPY-ir and somatostatin-ir fibers show a similar temporal pattern, although at the same age, somatostatin-ir CAxs are fewer than NPY-ir CFs. Both fiber types are present at birth (Woodhams et al., 1985; Ding and Elberger, 2000). Their density increases with age, peaking around P7–P10, and then decreases until adulthood (Woodhams et al., 1985; Ding and Elberger, 2000). The morphology of NPY-ir CAxs changes during development. Growth cones, easily identified in the first few postnatal days, gradually diminish and are observed only occasionally at P13. At P15–P17, some NPY-ir axons exhibit short ramifications called “short pedicles” or “spinelike appendages” (Ding and Elberger, 1994). At the same time of postnatal development, several NPY-ir CAxs show an unusual configuration, where fibers heading toward the contralateral hemisphere cross the midline, then loop back to their hemisphere of origin. Between P2 and P36, NPY-ir CAxs show relatively large, round/ovoid varicosities, whose size, shape, and density vary with age. At P17–P22, the varicosities shrink and became opaque; from P27 onward, their densities decrease and several CFs have a smooth appearance (Ding and Elberger, 1994). A small number of NPY-ir processes are found in the cat CC at all ages, including adults, without detectable age-related changes (Hogan and Berman, 1992). Their presence suggests that at least in the first few postnatal days, a large number of cortical neuronal cell bodies containing peptides participate in the formation of callosal connections.

#### 2.2.4 Possible functional implications of ICNs

##### 2.2.4.1 ICNs contacting CSF

**FIGURE 13:**
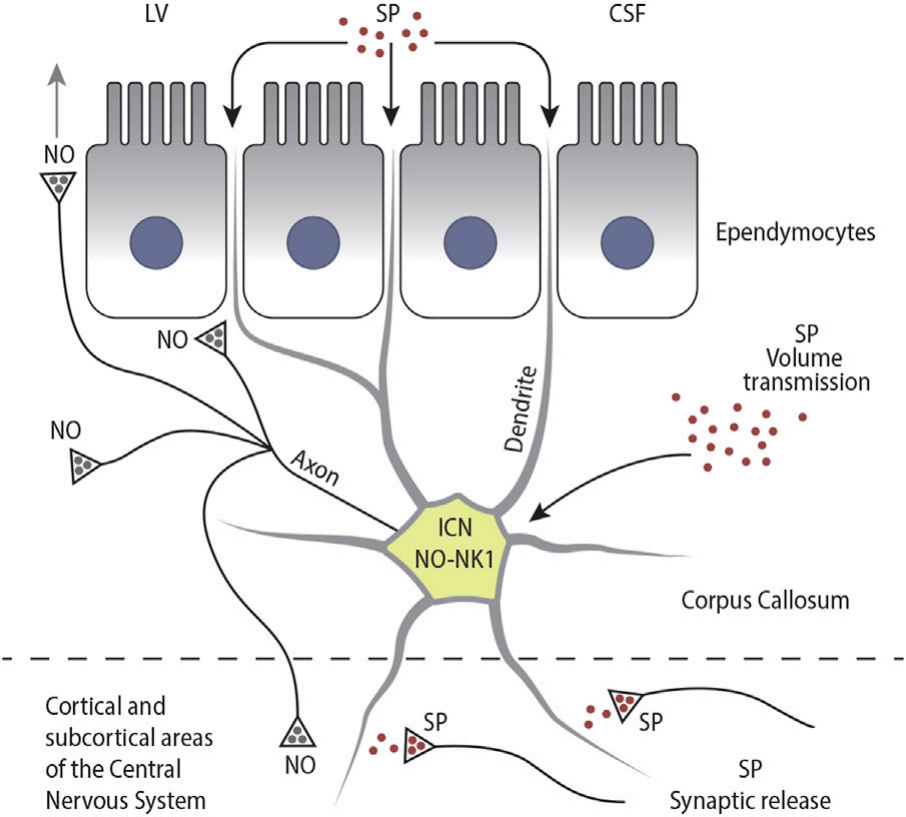
Schematic drawing illustrating the different modes of activation of NK1-ICNs by SP: I) by SP circulating in the CSF; II) via volume transmission; and III) via synaptic release. In turn, NK1-ICNs could release NO into the callosal parenchyma, influencing the activity of other ICNs, ependymocytes, and CFs. NK1-ICNs could also release NO into the CSF either directly or through diffusion and release NO into the cerebral cortex.

In the ventral part of the CC, there are two morphologically distinct cellular layers lining the LV: i) the ependyma, which forms a continuous layer around the brain ventricles, separating the CSF from the callosal parenchyma, and ii) the adjacent subependyma, which is only a few cells thick (Morshead and van der Kooy, 2001; Vigh et al., 2004). In these layers, immunocytochemical and histochemical studies have described cell bodies, dendrites, and axons of several neurons expressing NK1_R_ (Figures 5E,F; Barbaresi et al., 2015; Barbaresi et al., 2017), NO (Figures 5A–D; Sancesario et al., 1996; Barbaresi et al., 2014; Barbaresi et al., 2015), or both (Barbaresi et al., 2015). Neurons in the ependymal layer display different morphologies: fusiform (46.76%), polygonal (25.17%), round (19.06%), and pyramidal (8.99%) (Barbaresi et al., 2014). A dense network of dendrites, partly originating from neurons in the ependymal and subependymal layers and partly from neurons in the body of the CC, have been detected very close to the roof of the LV, suggesting that they might be in contact with the CSF (Figures 5E,F, 9B; Barbaresi et al., 2014; Barbaresi et al., 2015; Sancesario et al., 1996). The dendrites of the CSF-contacting neurons expressing both NO and NK1_R_ may be sensitive to SP circulating in the CSF (Tam et al., 1985; Zubrycka and Janenka, 2002; Muñoz and Coveñas, 2014). Since NK1_R_^+^ ICNs send their axons and terminals to the CC (Sancesario et al., 1996; Barbaresi et al., 2014; Barbaresi et al., 2015) and/or to some CNS areas (see above; Engelhardt et al., 2011), they can conceivably release NO in these regions. The dendritic spines on ICNs located in the proximity of the CSF suggest that they may receive synaptic input from other CNS regions (Engelhardt et al., 2011; see below), which can be integrated with responses to SP circulating in the CSF (Barbaresi et al., 2015). Thus, the hypothesis advanced by Vigh et al. (2004) that “*the chemical information taken up by the CSF-contacting neurons from the ventricular CSF may influence other areas of the CNS*” would apply to the ICNs expressing both NO and NK1_R_ in the ependymal and subependymal layers (see also Sancesario et al., 1996; Barbaresi et al., 2014; Barbaresi et al., 2015). ICN axons run parallel to the LV wall for several tens of microns (Figure 4A), then head toward the ventricular surface, probably terminating on or wedging between ependymal cells. ICNs can participate in the secretory processes involved in ventricular CSF formation either by releasing NO directly into the CSF (direct release model) or by facilitating, through NO release, the presynaptic release of a still unknown neurotransmitter that, by acting on ependymocytes, would induce neuropeptide release from them (amplification model; Alpár et al., 2019). The two models probably coexist (Figure 13; Alpár et al., 2019; Leak and Moore, 2012; Kuriyama and Ohkuma, 1995). The CSF concentrations of NO and its metabolites can help diagnose several neurological diseases (Orts-Del’Immagine and Wyart, 2017; Bratasz et al., 2004; Kho et al., 2021).

##### 2.2.4.2 Blood flow regulation by ICNs

NO is considered an atypical neurotransmitter involved in a variety of physiological functions such as cerebral blood flow (CBF) regulation (Iadecola, 1993; Iadecola, 2004). In the CC, the soma, dendritic processes, and axon terminals of NO-producing neurons are closely associated with the blood vessels (Figure 7; Barbaresi et al., 2014; Rockland and Nayyar, 2012; Sagrati et al., 2023). In rats, these cells account for approximately 38% of the entire NO-producing ICN population (Barbaresi et al., 2014), although this proportion is probably underestimated because it has not been always possible to relate NADPHd^+^/NOS^+^ cytoplasmic processes to any labeled cell body (Figure 7A; Barbaresi et al., 2014). Neuronal processes branch widely around an intracallosal blood vessel and its branches; often, more than one neuron cluster around a vessel (Figure 7A). Such an organization can be viewed as a multiple active NO source in the CC, covering a large diffusion area with a radius of at least 500 μm (Wood and Garthwaite, 1994), thus influencing a large number of neuronal elements as well as intracallosal blood vessels. NO participates in the maintenance of the resting cerebrovascular tone. A reduction in the resting CBF has been induced using different NOS inhibitors (Iadecola, 1993; Wang et al., 1995). It has also been hypothesized that nNOS accumulated in dendrites that are in close apposition to blood vessels may be involved in local NO production, induced by Ca^2+^ entry during dendritic depolarization. NO diffuses from these processes and causes activation of soluble guanylyl cyclase, present in neighboring brain blood vessels, inducing vasodilation. This would not require depolarization of the entire cell (Iadecola, 1993). Alternatively, excitatory synaptic events on ICNs (see below) could lead to the generation of propagated APs with a consequent increase in NO production and its local release from intracallosal axon terminals. Moreover, some ICNs have long-distance projections and could release NO in the overlying gray matter (GM), thus influencing both the vascular tone of cerebral cortex vessels and the functional activity of cortical neuronal networks (Figure 13; Kara and Friedlander, 1998; Engelhardt et al., 2011; Tomioka et al., 2005; Tomioka and Rockland, 2007; Higo et al., 2007).

##### 2.2.4.3 Neuronal signaling to ICNs

Neuronal NOS^+^ ICNs are subject to the potent excitatory effect of SP (Lamour et al., 1983; Jones and Olpe, 1984; Dittrich et al., 2012). A CC confocal microscopy study in rats has demonstrated that NK1_R_ is expressed (NK1_R_^+^) by the overwhelming majority of NO-producing neurons (Barbaresi et al., 2015). In the CC, similar to other CNS regions, stimulation of NK1_R_-expressing ICNs by SP can lead to activation of G_q_ protein, that in turn activates β-type phospholipase C, which hydrolyzes phosphatidylinositol bisphosphate to diacylglycerol (DAG) and inositol trisphosphate (IP_3_). IP_3_ acts as the second messenger to mobilize endoplasmic reticulum (ER)–stored Ca^2+^ via activation of specific receptors, whereas DAG activates protein kinase C (PKC) open L-type calcium channels in the plasma membrane (Mantyh et al., 1984; Khawaja and Rogers, 1996; Endo et al., 2014; Matsumura et al., 2021). The increase in [Ca^2+^]_i_ activates nNOS, a calmodulin-requiring enzyme, which catalyzes NO production from arginine (Bredt and Snyder, 1990; Hope et al., 1991). Different SP sources can influence ICN activity. The cerebral cortex, striatum, and hippocampus are all reached by the wide dendritic arborization of ICNs (Figure 10; Barbaresi et al., 2014; Barbaresi et al. 2015; Barbaresi et al. 2020; Engelhardt et al., 2011). In the rat cerebral cortex, SP-immunopositive neurons make up 2%–3% of all neurons and are found throughout the cortical layers, whereas SP^+^ fibers and terminals show different distributions in different areas. Moderate bilaminar ICC labeling in layers II and IV has been observed in the temporal and dorsal prefrontal cerebral cortices, whereas in the medial prefrontal cerebral cortex, SP labeling has been detected throughout layers II to IV. The network is less dense in the other cortical layers (Ljungdahl et al., 1978; Penny et al., 1986; Iritani et al., 1989). Notably, the neocortex receives important SP innervation from the ascending reticular system. Combined retrograde tracing and ICC experiments, conducted to study the SP cortical projection from the laterodorsal tegmental (TLD) nucleus, show that true blue injection into the prefrontal cortex labeled numerous TLD nucleus neurons on the injected side and, to a lesser extent, on the contralateral side. ICC revealed that several TLD nucleus projecting neurons contain SP (Vincent et al., 1983b). Another possible SP source is the striatum, which contains numerous SP^+^ neurons (Li et al., 2000; Li et al., 2001). Here, several SP-containing neuron projections emit numerous axonal branches with multiple varicosities, interpreted as SP-releasing zones (Li et al., 2001). A third potential SP source is the hippocampus. Here, SP^+^ fibers exhibit elongated or small round varicosities, whereas SP-ir puncta lie around or among the somata of individual neurons. Several of these terminals originate from local interneurons or from sources outside the hippocampus (Roberts et al., 1984; Davies and Köheler, 1985; Shults et al., 1987; Iritani et al., 1989). An important SP projection to the hippocampus originates from the septal area. Other more caudal nuclei, whose fibers pass through the septal area, are further SP sources to the hippocampal formation (Vincent and McGeer, 1981). Electrolytic lesions in this area significantly reduce SP levels in the hippocampus. Although SP terminals could contact NK1^+^ ICNs via classic synaptic transmission (direct synaptic contact), there is a marked spatial mismatch between NK1_R_- and SP-containing axon terminals in several CNS regions (Liu et al., 1994; Nakaya et al., 1994; Li et al., 2000; Vruwink et al., 2001; Wolansky et al., 2007; Dittrich et al., 2012). Therefore, SP could act on ICN NK1_Rs_ also through a non-synaptic/paracrine mechanism (or volume transmission; Agnati et al., 1995), whereby SP would reach distant NK1_Rs_ by diffusing into the extracellular space (Liu et al., 1994; Nakaya et al., 1994; Li et al., 2000; Vruwink et al., 2001; Wolansky et al., 2007; Dittrich et al., 2012). NO-producing ICNs may be activated by the afferents reaching the three structures. Since the caudate putamen (CPu–striatum) is considered a hub, receiving information from different brain regions such as the neocortex, thalamus, and midbrain (Fino and Venance, 2011; Silberberg and Bolam, 2015), it is conceivable that NO-producing ICNs receive inputs, albeit indirect, from all these structures. Moreover, thalamocortical and claustrocortical afferents have been shown to form a plexus at the level of layer VI of the cerebral cortex (Zhang and Deschenes, 1998; Arnold et al., 2001; Oda et al., 2004). From here, these afferents and layer VI neurons could contact ICN dendrites, which may also receive synaptic inputs from the collaterals of cortical afferent and efferent systems.

##### 2.2.4.4 NO action on developing CAxs

NO released by SP stimulation (via synaptic contact or volume transmission) can play an important role in CF myelination in the first month of life (Seggie and Berry, 1972; Valentino and Jones, 1982). NO diffusing into the adjacent callosal parenchyma interacts with the soluble isoform of guanylyl cyclase, triggering production of the classic second messenger guanosine 3′-5′-cyclic monophosphate (cGMP) in oligodendrocytes (OLGs; Tanaka et al., 1997). cGMP ICC, performed in brain slices of immature rats aged 1–4 weeks, demonstrated cGMP in callosal OLGs. cGMP labeling of OLGs diminished progressively with age, suggesting a role for NO in OLG development and related myelinogenesis (Tanaka et al., 1997). Indeed, nNOS-deficient mice show a remyelination delay following chemical demyelination (Linares et al., 2006), and Sprague–Dawley pups inhaling NO-enriched air in the first postnatal week show increased CAx myelination (Olivier et al., 2010). In the rat CC, NO released by ICNs could affect differentially the CFs that seek their target in the contralateral cortex and the exuberant CAxs that are destined to be eliminated or do not enter the cerebral cortex at all. In the former case, NO could promote the filopodial extension that drives the growth cone toward its target in the contralateral cortex (Gally et al., 1990) by stimulating the enzyme guanylyl cyclase, which in turn increases cGMP production. cGMP activates PKG, which in turn leads to production of cyclic ADP-ribose (cADPR). cADPR causes Ca^2+^ release from intracellular stores via gated ryanodine receptors. The [Ca^2+^]_i_ increase affects filopodia morphology directly via binding to calmodulin (Van Wagenen and Rehder, 1999; Welshhans and Rehder, 2007). In the case of the exuberant/transient CAxs, NO could promote the retraction of such axons and of their collaterals and the stabilization of axons with appropriate connections in the first postnatal days (Gally et al., 1990; Kadhim et al., 1993; Wu et al., 1994; Ernst et al., 2000; Mize and Lo, 2000). Although the mechanism is still to be elucidated, exposure to BDNF and NO has been reported to stabilize correctly targeted branches, possibly by altering actin cytoskeleton dynamics (Ernst et al., 2000).

##### 2.2.4.5 Blood oxygen-level–dependent signal effect

In addition to the functions described above, NO-expressing ICNs could be involved in the blood oxygen-level–dependent signal (BOLD) signal (Barbaresi et al., 2014; Sagrati et al., 2023). The BOLD effect is a complex and still not completely understood hemodynamic signal. It consists of an increase in local blood flow accompanied by an increased oxyhemoglobin concentration related to the neuronal activity of a particular area of the brain (Iadecola, 2004; Schaeffer and Iadecola, 2021), which has been investigated by functional brain imaging, such as fMRI (Mazerolle et al., 2010; Schaeffer and Iadecola, 2021). Hemodynamic changes induced by motor and visuomotor tasks and peripheral stimulation (Mosier and Bereznaya, 2001; Tettamanti et al., 2002; Omura et al., 2004; Weber et al., 2005; D’Arcy et al., 2006; Mazerolle et al., 2010; Fabri et al., 2011) can evoke responses in the WM and in specific CC regions (Figure 3). These responses could relate to the presence of NO-producing ICNs, whose depolarization can cause an increase in blood flow (Iadecola, 1992; Iadecola, 1993). Such depolarization may occur in two ways. First, excitation of some CNS regions via peripheral stimulation can induce depolarization of those NO-producing ICNs whose dendritic trees reach the activated overlying cerebral cortex, hippocampus, or striatum (see above); this in turn could induce NO release from neuronal processes associated with intraparenchymal callosal vessels. Second, an increase in cortical neuronal activity—consistent with the fact that most axons traversing the CC originate from Gluergic neurons (Barbaresi et al., 1987; Elberger, 1989; Conti et al., 1988a; b; Giuffrida and Rustioni, 1989; Dinopoulos et al., 1989; Dori et al., 1992)—induces axonal Glu release in the CC (Kukley et al., 2007; Ziskin et al., 2007), possibly exciting NO-producing ICNs (Iadecola and Nedergaard, 2007) through *N*-methyl-D-aspartate receptors (NMDA_Rs_; Garthwaite, 1991; Garthwaite, 2008). Double-labeling immunofluorescence experiments performed in the rat CC have indicated that nearly all NK1^+^ ICNs contain nNOS, whereas 84.59% of nNOS-containing neurons are NK1^+^. These data suggest that all NK1^+^ ICNs can release NO through the action of SP and that the CC contains two populations of NO^+^ ICNs, one of which is not activated by SP but could release NO via alternative mechanisms, probably through the action of Glu from CFs acting on NMDA_Rs_ (Barbaresi et al., 2015). Moreover, it cannot be excluded that SP and Glu may act on the same ICNs. An immunofluorescence study (Lin et al., 2008), where NK1_R_ and NMDA_R_ immunoreactivity colocalized in the same neurons in the rat nucleus tractus solitarii, supports this hypothesis. Glu interaction with NMDA_Rs_ can therefore be necessary for BOLD responses in the CC, as in other CNS regions, where NMDA_R_ antagonists attenuate blood flow responses (Iadecola et al., 1996; Nielsen and Lauritzen, 2001; Gsell et al., 2006; Busija et al., 2007; Hoffmeyer et al., 2007; Tiede et al., 2012). However, the concomitant role for astrocytes (ASs) in callosal neurovascular coupling (Attwell et al., 2010) cannot be ruled out. Current findings indicate that GCs lack NO-producing enzymes. Thus, Glu released from CAxs can induce the release of vasoactive agents other than NO from AS end-feet, like cyclo-oxygenase products, whose inhibition significantly reduces vasodilation (Zonta et al., 2003; Takano et al., 2006).

##### 2.2.4.6 AChE-containing ICNs

AChE is a highly efficient enzyme that hydrolyzes ACh at cholinergic synapses (Zimmerman and Soreq, 2006; Silman and Sussman, 2008). An immunocytochemical study has described several AChE-rich neurons and fibers (Rockland and Nayyar, 2012) in the monkey CC, although ACh levels in this region are absent or at least unreported. In addition, an increasing number of studies suggest that “non-cholinergic functions” of AChE, such as neurogenesis, neurotransmitter recycling, blockade of excitatory amino acid uptake, and neurite outgrowth (Appleyard, 1994; Zimmerman and Soreq, 2006; Silman and Sussman, 2008), may be important for intrinsic callosal activity.

##### 2.2.4.7 Peptidergic ICNs and ICFs

###### 2.2.4.7.1 Neuropeptide Y

NPY, a polypeptide composed of 36 amino acid residues, is widely distributed throughout the central and peripheral mammalian nervous systems, such as in the human brain (Lundberg et al., 1982; Adrian et al., 1983; Nakagawa et al., 1985; De Quidt and Emson, 1986; Dyzma et al., 2010). It is involved in the regulation of several physiological functions such as learning, cognition, thermoregulation, sleep, cardiovascular dynamics, and neural diseases (Dyzma et al., 2010; Li et al., 2019). Moreover, NPY causes a pronounced concentration-dependent contraction of cerebral arteries both *in vitro* and *in situ* (Estrada and DeFelipe, 1998; Li et al., 2019). NPY-like immunoreactivity has been detected in neuronal cell bodies and fibers in several CNS regions (Adrian et al., 1983; Nakagwa et al., 1985; De Quidt and Emson, 1986), which include the CC (Ding and Elberger, 1994; Nakagawa et al., 1985; Jovanov-Miloševic’ et al., 2010; Hogan and Berman, 1992; Chronwall et al., 1985; Woodhams et al., 1985; Yoshimura et al., 2009; see above). Little is known of the function or regulation of NPY-expressing neurons in the CC. However, the high colocalization rate of NADPHd/nNOS and NPY^+^ neurons found in several CNS regions (Huh et al., 1997; Vincent et al., 1983a; Estrada and DeFelipe, 1998) suggests that nitrergic vasodilator ICNs and NPY^+^ vasoconstrictor ICNs could be involved in combined local blood flow regulation in the CC.

## 3 Non-neuronal elements

### 3.1 GCs and intracallosal GCs

In the CNS, the main GC types are OLGs, ASs, and microglia (Gundersen et al., 2015). In the rat CC, the GC population comprises ∼75% OLGs, ∼20% ASs, and ∼5% microglia (Mori and Leblond, 1970). A fourth type of neuroglial cell, identified by its expression of NG2 chondroitin sulfate proteoglycan (NG2 glia; Nishiyama et al., 1999), has also been described in the CC. NG2 cells are already detected in the early postnatal days and are able to differentiate into OLGs. Their numbers decline with age, but they are still present in the adult CNS, when they can differentiate to adult OLGs (for a review, see Nishiyama et al., 1999; Peters, 2004). OLGs produce the insulating myelin sheath, which is essential for AP propagation along axons and to preserve axon integrity via production of neurotrophic factors (Gundersen et al., 2015). ASs are involved in a variety of functions such as the regulation of ion and neurotransmitter concentrations in the extracellular space, the promotion of synapse formation, neuronal spiking regulation, synaptic plasticity, the refinement of neural connectivity, and the formation and maintenance of the blood–brain barrier (BBB) and brain blood flow (Gundersen et al., 2015). As regards microglial cells, they have a small soma and branched morphology with fine cellular processes. They are involved in CNS development, neuronal connectivity, and physiological synaptic stripping/pruning (Gundersen et al., 2015).

#### 3.1.1 Oligodendrocytes

##### 3.1.1.1 Classification of intracallosal oligodendrocytes

Three OLG classes have been described in the CC of young rats: light, medium, and dark.

**Light OLGs.** Light OLGs have a wide soma with a large pale nucleus and cytoplasm. The nuclear diameter ranges from 6 to 8.5 μm; fine unbranched processes, originating from the perikaryon, contain 1–20 microtubules, which continue along their course. Light OLGs make up approximately 6% of CC GCs (Mori and Leblond, 1970).

**Medium OLGs.** These cells are smaller than light OLGs and have an oval nucleus whose diameter averages from 4 to 7 μm. The cytoplasm is denser and less abundant than in light OLGs; fine processes containing microtubules depart from the soma. Medium OLGs account for ∼25% of callosal GCs (Mori and Leblond, 1970).

**Dark OLGs.** Dark OLGs are among the smallest GCs; they have a dense cytoplasm and a round nucleus measuring 3.5–5.5 μm. Several lamellar bodies are associated with the membranes of their organelles and their inner surface. A small number of short processes with the same density as the cytoplasm sprout from the soma. Dark OLGs account for ∼40% of the callosal glial population (Mori and Leblond, 1970). Notably, in young rat CC, ^3^H-thymidine autoradiography has documented that proliferating light OLGs are the most actively dividing cells, transforming into medium OLGs and ultimately into postmitotic dark OLGs (Mori and Leblond, 1970).

##### 3.1.1.2 Oligodendrocyte organization in the CC

OLGs are a morphologically heterogeneous population (Mori and Leblond, 1970). In the CNS, they form multilamellar, spirally wrapped myelin sheaths around neuron axons, which allow rapid saltatory conduction and provide metabolic support to neurons. Callosal OLGs are arranged in rows between the nerve fibers (Domercq and Matute, 1999; Tanaka et al., 2021) and have therefore been called interfascicular OLGs (I-OLGs; Peters et al., 1991). I-OLGs have two types of organizations: short chains of three or more mature OLGs (row I-OLGs) or single OLGs (isolated I-OLGs). The cell bodies of both types are morphologically oriented. Thick processes extending from the cytoplasm-rich part of the cell participate in myelin sheath formation. Using serial block scanning EM, these processes have been classified into i) those that myelinate an axon without branching and ii) those where each of the several branches myelinates a single axon. In either type, a single cytoplasmic extension forms myelin sheaths around distant axons and seldom forms multiple sheaths around a single axon. Measurements of the I-OLG processes wrapped around CAxs suggest that each I-OLG has a characteristic myelination pattern and/or a preferred target axon diameter. Moreover, adjacent myelin sheaths have a similar thickness, even if they are derived from different I-OLGs. These data suggest that a single axon may influence myelin sheath thickness, probably by releasing axon-derived soluble factors like neuregulin-1 (Lemke, 2006; Tanaka et al., 2021). In rodents, most OLGs develop in the first few postnatal weeks, differentiating from migratory proliferative OLG precursor cells (OPCs) originating from the CNS ventricular zones. They are also called polydendrocytes, due to their highly branched processes, and can be identified by the expression of NG2 chondroitin sulfate proteoglycan antigen (Rivers et al., 2008; Gundersen et al., 2015).

##### 3.1.1.3 Oligodendrocyte precursor cells

###### 3.1.1.3.1 Origin of oligodendrocyte precursor cells

Neural stem cells differentiate into OPCs in the neuroepithelial zones around the ventricles, under the influence of the OLG-specific transcription factors Olig1/2, Nkx2.2, Sox10, and the endogenous retinoic acid (RA)–synthesizing enzyme retinaldehyde dehydrogenase 2 (RALDH2). OPCs require the vasculature as a physical substrate to migrate to their final position (Tsai et al., 2016), where they proliferate under the regulation of transcription factors such as Id2, Id4, Tcf4, and Hes5. In turn, OPCs differentiate into immature premyelinating OLGs and ultimately into mature OLGs, which produce the myelin sheath (Nishiyama et al., 1999; Salzer and Zalc, 2016; van Tilborg et al., 2018; Morrison et al., 2020).

###### 3.1.1.3.2 Relationship between oligodendrocyte precursor cells and CFs

As noted above, unmyelinated CAxs are endowed with a discrete synaptic machinery that forms synaptic specializations with NG2^+^ OPCs (Kukley et al., 2007; Gallo et al., 2008). NG2^+^ cells appear in the CC before birth (Ziskin et al., 2007); they have a small (10–15 μm) bipolar cell body and an intricate tree of processes extending predominantly from the poles of the soma for ∼100 μm (Dawson et al., 2003; Kukley et al., 2007; Fröhlich et al., 2011). Multiple small varicosities (nodules), found along these processes, may be branching points or points of synaptic contact (Fröhlich et al., 2011). NG2^+^ OPCs can proliferate throughout their life, transferring both their morphological and functional features, such as synapses, to daughter cells during mitosis (Dawson et al., 2003; Kukley et al., 2008; Ge et al., 2009). Given their position at the boundary between functionally distinct axon bundles, NG2^+^ OPCs might be involved in regulating the development and growth of CAxs originating from the same cortical area and being directed toward the contralateral hemisphere, as well as influencing the myelination of adjacent axon bundles (Mangin and Gallo, 2011).

##### 3.1.1.4 Receptors on oligodendrocyte precursor cells (NG2^+^) and oligodendrocytes

###### 3.1.1.4.1 Ionotropic Glu receptors

Glu release through vesicle fusion occurs at the axon–glia interface, which consists of small drop-like protrusions (50–100 nm wide) of the axon membrane into NG2^+^ OPCs. In these cells, axonal Glu release induces quantal α-amino-3-hydroxy-5-methyl-4-isoxazolepropionic acid receptor (AMPA_R_)–mediated currents (Kukley et al., 2007; Ziskin et al., 2007) and entry of Ca^2+^ ions via the Ca^2+^-permeable AMPA_Rs_ lacking Glu_R2_ subunit (Kukley et al., 2007; Table 3). Although NG2^+^ cells appear in the CC before birth, these currents are not recorded in such cells until postnatal day P5. Currents are small in young animals (P7–P9; amplitude range, 5–30 pA) and increase with age, reaching 40–400 pA in mature animals (P20–P30). Currents have also been elicited in NG2^+^ OPCs of adult mice (Ziskin et al., 2007). AMPA_R_-mediated currents grew progressively with increasing stimulus intensity, suggesting that each NG2^+^ OPC receives glutamatergic input from hundreds of synaptic contacts (Bergles et al., 2000; Kukley et al., 2007; Ziskin et al., 2007). However, in the CC, axon–glia synaptic contacts are rapidly lost as NG2^+^ OPCs differentiate into OLGs (De Biase et al., 2010). Furthermore, activation by Glu of Ca^2+^-permeable AMPA_Rs_ on NG2^+^ OPCs induces NG2^+^ OPC proliferation and/or differentiation to myelinating OLGs. Indeed, blockade of spiking activity or AMPA_Rs_ adversely affects myelination. Moreover, OPCs lacking all three AMPA_R_ subunits (GluA2, GluA3, and GluA4) receive a reduced synaptic input. This in turn affects OLG lineage development, resulting in ∼25% reduction in OLGs and in ∼20% fewer myelin sheaths (Kukley et al., 2007; Ziskin et al., 2007; Rivers et al., 2008; Fannon et al., 2015; Kougioumtzidou et al., 2017). A key role of AMPA_R_ in early postnatal life has also been shown by other studies. The distribution of AMPA_R_ subunit Glu_R4_ was investigated by IHC in the CC of immature and mature rats using LM and EM. In the rat CC, Glu_R4+_ OPCs were few in rats aged 1–2 days, increased at 7–10 days, and were rare in adult rats (Ong et al., 1996). Randomly scattered Glu_R4+_ OPCs have also been observed in the entire adult bovine CC (García-Barcina and Matute, 1998).

**TABLE 3:** Receptors in NG2/OLG cells.

Receptor type	Species (age)	Techniques	Types, localization, function	References
Ionotropic Glu_Rs_				
AMPA				
Glu_R4_	Rats (P1–P2; P7–P10–adult). Bovine (adult)	IHC, LM, EM	Glu_R4_-OPCs were few in P1/P2 rats; they increased in P7 rats and were rare in adult rat and bovine CC. They could play a part in regulating OLG development	Ong et al. (1996), García-Barcina and Matute (1998)
NMDA				
NR1 subunit	Rats (P7–P14). Developing and mature mice	IHC, EM	NR1 subunit was detected in OLG myelinating processes, in the outer- and innermost membranes, and in myelin. In mouse CC, NMDA_R1_-deficient OPCs exhibited normal rates of development and physiological morphological properties. These data suggest that NMDA_R_ signaling plays no role in NG2^+^/OPC development/proliferation. They could regulate AMPA_R_-dependent signaling with surrounding axons	De Biase et al. (2011), Karadottir et al. (2005), Micu et al. (2006), Ziskin et al. (2007)
Kainate				
Subunits Glu_R5-7_ Glu_R6-7_ KA-2	Bovine (adult)	IHC	Glu_R5-7_ immunoreactivity (IR) was found in 49% of OLGs; Glu_R6-7_ IR was found in ∼34% of OLGs, and KA-2 IR was found in 37% of OLGs. GluR_5-7_ and KA-2 subunits were co-expressed in the majority of macroglial cells. Kainate receptors may be important in axon-to-glia and/or glia-to-glia signaling	García-Barcina and Matute (1998)
Metabotropic Glu_Rs_				
mGlu_R5_	Rats (P4–P7)	ICC	mGlu_R5_ is strongly expressed in callosal OLGs of P4 and P7 rats. Changes in [Ca^2+^]_i_ levels are due to calcium release from the ER. The Glu thus released could activate in a paracrine manner I-OLG mGlu_Rs_, which may be involved in regulating OLG division	Kukley et al. (2007), Luyt et al. (2003), Di Giorgi Gerevini et al. (2004)
Dopamine receptors (D_Rs)_				
D_3R_	Mice (P3, P9, P14, P25–adult)	RT-PCR, ICC	Transient D_3R_ expression occurred in precursors and immature OLGs but not in mature OLGs. ICC showed D_3R_ in I-OLGs located in the genu of the CC. D_3R_ was detected only in the CC at the developmental stage at which OLGs are beginning to envelop axons. Although the functions of these receptors require further investigation, D_3R_ expressed at different stages in OLG development may be involved in regulating myelin formation	Bongarzone et al. (1998)
D_2R_	Rats (P5, P7, P10, P15, P20, P25, and adults: P90–P120). Mice (P3, P9, P14, P25, and adults)	IHC, *in situ* hybridization, Northern blotting, RT-PCR, ICC	Cells containing D_2R_ mRNA became evident from P15 to P20, around the peak of myelination, and had the morphological characteristics of I-OLGs. Although the functions of this receptor remain to be elucidated, D_2R_ may be involved in the regulation of myelin formation	Bongarzone et al. (1998), Howard et al. (1998)
Purinergic receptors				
Metabotropic P_2y1_	Bovine. Rats	Northern blotting, RT-PCR, ICC	Rat and bovine CC is rich in mRNA-encoding functional ATP receptors. The molecular subtype of ATP receptor predominantly expressed in the CC is P_2y_ purinoceptor. ATP may have trophic effects during development and/or following injury	Agresti et al. (2005b), Morán-Jiménez and Matute (2000), Deng et al. (1998)
Ionotropic P_2x7_	Rats (adult)	RT-PCR, *in situ* hybridization, combined IHC, and *in situ* hybridization	P_2X7_ mRNA colocalizes with the OLG marker CNPase, indicating that P_2X7_ is expressed in OLGs. Its functions in the CC are unknown. Findings from other CNS regions suggest that P_2X7_ receptor may play a variety of roles in a wide range of cell types in the brain	Yu et al. (2008)

Callosal OPCs and OLGs express functional NMDA_Rs_ for Glu in addition to AMPA_Rs_ (Káradóttir et al., 2005; Ziskin et al., 2007). NMDA_Rs_ are oligomeric ligand-gated ion channels formed by several subunits. Some subunits have been cloned and consist of an essential subunit, NR1, and various modulating subunits: four NR2 subunits (NR2A, NR2B, NR2C, and NR2D) and two NR3 subunits (NR3A and NR3B). Activated NMDA_Rs_ become permeable to Na^+^, K^+^, and Ca^2+^. NR1 has a unique role in determining the activity of the endogenous NMDA_R_, contains the glycine binding site, and is critical for functional NMDA_R_ formation (Forrest et al., 1994; Wong, 2006; DeBiase et al., 2011). Electron microscopic immunochemistry has documented the NR1 subunit in OLG myelinating processes, in the outer- and innermost membranes, as well as in myelin (Káradóttir et al., 2005). *In vitro* studies of various CNS regions indicate that NMDA_Rs_ induce intracellular calcium [Ca^2+^]_i_ transients and likely play a role in controlling OLG development and myelination (Káradóttir et al., 2005; Micu et al., 2006; Ziskin et al., 2007). By contrast, an *in vivo* study of the CC of developing and mature mice with genetic deletion of the NMDA_R1_ subunit from OPCs and their OLG progeny has found that NMDA_R1_-deficient OPCs exhibited normal rates of proliferation, oligodendrogenesis, and myelination; had preserved physiological and morphological properties; and formed synapses with glutamatergic axons, although they displayed an enhanced expression of Ca^2+^-permeable NMDA_Rs_. These data suggest that NMDA_R_ signaling plays no role in NG2^+^ OPC development/proliferation but regulates AMPA_R_-dependent signaling with surrounding axons (De Biase et al., 2011). Notably, NMDA_Rs_ are expressed in ∼60% of callosal OPCs (Ziskin et al., 2007). Consistent with these data, De Biase et al. (2011) found that 22%–33% of callosal OPCs did not exhibit an NMDA_R_-mediated current. These findings support the existence of two separate populations of callosal NG2^+^ OPCs (Ziskin et al., 2007; De Biase et al., 2011; Mangin and Gallo, 2011), whose local functional significance remains to be elucidated. However, given their proximity to ASs and microglia in glial scars, NG2^+^ OPCs expressing NMDA_Rs_ might facilitate the detection of local lesions and promote their repair (De Biase et al., 2011).

Samples of bovine CC were used immunocytochemically to study the presence and distribution of cells expressing kainate-glutamate receptor subunits. Analysis of the results reveal that GluR5–GluR7 subunits are expressed in approximately 50% of OLGs while GluR6–GluR7 and KA2 subunits in approximately 35% of OLGs. In addition, double-labeling experiments indicate that the majority of OLGs expressing kainate receptors co-express GluR5–GluR7 and KA2 subunits. The wide distribution of these receptors in various fibrous systems such as in the CC has led to the hypothesis of their involvement in axon-to-glia and/or glia-to-glia signaling (García-Barcina and Matute, 1996).

###### 3.1.1.4.2 Metabotropic Glu receptors

In addition to ionotropic Glu_Rs_, callosal OPCs also express mGlu_Rs_. An ICC study has found a frequent colocalization of O4 and O1 OLG markers in brain sections from 4- to 7-day-old rats (Luyt et al., 2003; Table 3). These data suggest that mGlu_Rs_ are already present in early postnatal callosal development. mGlu_R_ activation induces [Ca^2+^]_i_ oscillations mediated by Ca^2+^ release from the endoplasmic reticulum (Luyt et al., 2003), which is typically associated with cell growth and proliferation (Di Giorgi Gerevini et al., 2004). Although there are no data on the presence of mGlu_Rs_ at the contact sites between CAxs and NG2^+^ OPCs, it has been reported that in several cases, small clusters of functional vesicles and fusion proteins are not associated at the axon–NG2^+^ OPC interface (Kukley et al., 2007). Glu released at these sites could thus activate, in a paracrine manner, I-OLG mGlu_Rs_, which may be involved in regulating OLG division (Luyt et al., 2003).

###### 3.1.1.4.3 Purinergic receptors

Different types of purinergic receptors have been described in the CC since the mid-1990s. Adenosine and ATP, applied to mouse callosal slices, act at the P1 and P2 purinoceptors, respectively, and increase cytoplasmic [Ca^2+^]_i_ (Table 3). The increase induced by adenosine occurs through InsP_3_-sensitive channels of the ER of GCs, whereas Ca^2+^ responses due to ATP consist of two components: fast release from intracellular stores (ER) and a slower Ca^2+^ influx from the extracellular space. Although several GCs appear to co-express both types of purinergic receptors, the GC type generating the two responses has not been determined (Kirischuk et al., 1995; Bernstein et al., 1996). In a study where an ATP receptor clone was isolated using the *Xenopus laevis* oocyte expression system, Northern blot and RT-PCR analysis of various bovine brain tissues documented high levels of ATP receptor mRNA in bovine CC. Moreover, *in situ* RT-PCR experiments demonstrated wide ATP receptor expression in several callosal cells, which based on their size and morphology seemed to be macroglia, OLGs, and ASs, and that the pharmacological profile of the receptor was consistent with P_2y1_ purinoceptor (Deng et al., 1998). These data have been confirmed by ICC studies. Characterization of a polyclonal antiserum to a carboxy-terminal epitope of P_2y1_ receptor and its use in immunolocalization studies in rat and bovine CNS have indicated that P_2y1_ is widely expressed by callosal ASs and OLGs (Morán-Jiménez and Matute, 2000). As regards the P_2x7_ subunit, P_2X7_ receptor mRNA expression has been described in several CNS regions including nerve fiber tracts such as the anterior commissure, optic tract, internal capsule, and CC. Double-labeling experiments for *in situ* hybridization in rats have employed the neuron-specific protein marker NeuN, the microglial marker CD11b, the AS marker glial fibrillary acidic protein (GFAP), and the OLG marker 2′,3′-cyclic nucleotide 3′-phosphodiesterase (CNPase) to identify the callosal cells expressing P_2X7_ receptor mRNA. Detection of CNPase^+^ cells indicated that only callosal OLGs expressed P_2X7_ receptor mRNA (Yu et al., 2008). Several studies suggest that purinergic P2 receptor is involved in the generation of Ca^2+^ waves, a type of long-distance signaling among GCs (ASs, OLGs, and their late precursors; Kirischuk, et al., 1995). ATP release from stimulated ASs, exogenously applied or co-released with Glu (Fields and Burnstock, 2006) into the extracellular space, is followed by purinergic receptor activation, which in turn induces [Ca^2+^]_I_ release from cytoplasmic stores (Kirischuk, et al., 1995). Ca^2+^ signals evoke ATP release from ASs to propagate the Ca^2+^ wave, activating other types of GCs (Kirischuk, et al., 1995; Bernstein et al., 1996; Schipke et al., 2002; Haas et al., 2006). Such waves spread from their site of origin over hundreds of micrometers. In the CC, electrical stimulation or local ATP ejection generates a Ca^2+^ wave over at least half a millimeter from the point of origin to the ventricular wall, most likely to ependymal cells (Schipke et al., 2002). In addition to their involvement in the generation of glia–glia signaling, purinergic receptors in callosal OLGs could play an important role in neuron–glia signal transfer in normal and pathological conditions (Kirischuk, et al., 1995; Bernstein et al., 1996; Deng et al., 1998). The precise physiological roles of P_2y_ and P_2x_ receptors in the CC are still unclear, and their functions can be inferred only from the results of experiments conducted in other CNS regions. *In situ* experiments indicate that P_2Y_ and P_2X_ have distinct roles in GC physiology and pathology. Metabotropic P_2Y_ receptor mobilizes [Ca^2+^]_i_ at physiological ATP concentrations, whereas inotropic P_2X_ purinoceptor induces a Ca^2+^ influx across the plasmalemma at high ATP concentrations, such as those occurring after CNS injury (James and Butt, 2001). P_2Y1_ is also expressed in OPCs. Double immunofluorescence experiments using an NG2 and a P_2Y1_ antibody detected several NG_2_^+^/P_2Y1_ OPCs in the cerebral cortex, subcortical WM, and the cerebellar granule layer. The addition of ATP and ADP to cultured oligodendroglial cell lineages stimulated OPC migration, inhibited their mitogenic responses, and promoted their differentiation (Agresti et al., 2005a). Moreover, P_2Y1_ is involved in repair processes in areas damaged by ischemia and might regulate remyelination in inflammatory demyelinating diseases like multiple sclerosis (Agresti et al., 2005b). According to another study, P_2X7_ is localized in the cytoplasm of myelinating Schwann cells and in the outer membrane facing the adjacent myelinated axons of the sciatic nerve; moreover, P_2X7_ knockout nerves possess significantly more unmyelinated axons than the wild type, and myelin thickness in the knockout nerve is unaltered. These findings suggest a larger role for P_2X7_ in SC maturation than in myelin formation (Faroni et al., 2014). The above studies provide indirect information on the functions mediated by P_2Y1_ and P_2X7_ receptors in the CC, which due to their strategic position could be relevant for OLG differentiation as well as myelin formation and conservation (Matute et al., 2007b; Matute, 2008).

Adenosine is a normal constituent of the extracellular space in the CNS. It can be generated by the action of ectoenzymes that dephosphorylate ATP, yielding ADP, adenosine monophosphate, and adenosine (Zimmermann, 1994). Adenosine is involved in neuronal response modulation and neurotransmission and in tissue protection from damage following ischemia (Liu et al., 2019). Several of the neuroprotective and neuromodulatory effects of adenosine are regulated by A1_R_, the most widely expressed adenosine receptors (A_R_) in the brain (Liu et al., 2019). An IHC study that has provided evidence for A1_Rs_ in rat CAxs has demonstrated that A1_Rs_ are physiologically active. The lipid-soluble agonist CPA produces a dose-dependent reduction of the evoked AP amplitude, probably due to increasing K^+^ conductance, an effect that is reversed by an A1_R_ antagonist. These data indicate that axonal A1_Rs_ can influence, i.e., modulate or block, callosal transmission (Swanson et al., 1998). Moreover, it is conceivable that axonal A1_Rs_ respond only to the adenosine released by CAxs because the hydrophilic nature of adenosine, found in interstitial liquid, would prevent it from passing through a structure with high lipid content, i.e., myelin (Swanson et al., 1998). In the CC, A1_Rs_ could be expressed on OLG membranes (Swanson et al., 1998), therefore adenosine released in the CC could also influence OLGs or OPCs (Swanson et al., 1998). Interestingly, a concentration-dependent increase in migration has been described in OPCs treated with adenosine or with the A1_R_ agonist CPA, suggesting that A1_Rs_ transduce the effects of adenosine, thereby stimulating OPC migration. OPC migration plays a role in myelin repair following local traumatic, inflammatory, or toxin-induced myelin loss (Othman et al., 2003). The role of A_Rs_ and adenosine in modulating enzymes in the CC warrants further investigation.

###### 3.1.1.4.4 Dopamine receptors

According to neuroanatomical studies, dopamine receptors (D_Rs_) are expressed in non-neuronal GM and WM elements. In binding site preparations of rodent, monkey, and human prefrontal cortex, 35% of D_2_ receptor (D_2R_) binding activity was associated with ASs (Khan et al., 2001; Table 3). The presence of D_2Rs_ on ASs has further been confirmed by light and electron microscopic observations. Double-labeling experiments with D_2R_ and GFAP antibodies detected the receptor in perisomatic AS processes surrounding cortical interneurons (Khan et al., 2001). D_2Rs_ were also documented in GCs in layers III and IV of the primary somatosensory cortex (Yu et al., 2019). Further evidence for DA_Rs_ in GCs has come from *in vitro* and *in vivo* experiments showing D_2_ class receptors in OLGs of WM regions, particularly the CC. D_3R_ was detected in primary glia cultures, both by RT-PCR (as early as 5 days) and by using double immunofluorescence analysis. Experiments using D_3R_ antibody and OPC markers have indicated that D_3R_ is expressed in OPCs and immature OLGs but not in mature OLGs. In line with these data, *in vivo* immunocytochemical results have shown D_3R_ in callosal GCs with the morphological features of interfascicular OLGs. D_3R_ labeling was visible from the early postnatal days, peaked at P14, and then declined. In the adult nervous system, only scattered callosal cells were labeled (Bongarzone et al., 1998). Northern blot analysis, *in situ* hybridization, and histochemistry have been used to study callosal D_2R_ distribution. Cells containing D_2r_ mRNA became evident from P15 to P20 (i.e., around the peak of myelination) and had the morphological characteristics of I-OLGs, having a somatic diameter of 5–7 μm and a very thin rim of perikaryal cytoplasm. Moreover, starting from P25, they formed “strings” of adjacent labeled cells, a common feature of I-OLGs (Howard et al., 1998). The function of D_2R_ and D_3R_ remains unclear. Given their peak expression during postnatal development, they may be involved in the callosal myelination process. D_3R_ could prevent OLGs from myelinating prematurely by delaying subsequent myelin sheath elaboration (Bongarzone et al., 1998), whereas D_2R_, whose expression peaks at P15–P25, coinciding with peak CC myelination (Seggie and Berry, 1972; Valentino and Jones, 1982; Mack et al., 1995), could be involved in extending oligodendroglia formation, which is integral to myelination (Howard et al., 1998).

##### 3.1.1.5 Proliferation and differentiation of callosal NG2^+^ oligodendrocyte precursor cells

The proliferation of OPCs and their differentiation into OLGs underpin callosal myelination. Experimental evidence has suggested that electrical activity and synaptic Glu release may regulate myelination both during postnatal development and in adulthood (Gallo et al., 2008).

Optogenetic stimulation of the premotor cortex in awake-behaving mice has demonstrated that activating subcortical WM projections in the CC elicited the mitogenic response of neural progenitor cells and OPCs, thus promoting oligodendrogenesis, and increased myelination in the deep layers of the premotor cortex and in subcortical CFs, thus improving the motor function of the corresponding limb. Moreover, intraperitoneal injection of the thymidine analog EdU (5-ethynyl-2′-deoxyuridine), which labels dividing cells, showed a rapid proliferative response of callosal OPCs beginning 3 hours after optogenetic stimulation (Gibson et al., 2014). In line with this work, pharmacogenetic stimulation of a small subset of somatosensory cortical neurons induced a significant increase in proliferating OPCs in the CC of juvenile and adult rats, several of which transformed into mature myelinating OLGs. The stimulated axons were more likely to be myelinated and have a thicker myelin sheath than neighboring non-stimulated axons (Mitew et al., 2018). Reduction of the activity of a subpopulation of transcallosal axons arising from SI, obtained through overexpression of the inward-rectifier potassium ion channel (Kir2.1; *in utero* electroporation technique), suppressed myelination of the inhibited axons without any effects on proliferating OPCs or adult OLGs (Mitew et al., 2018). These findings demonstrate that neuronal activity potentiates OPC proliferation (see also Clarke et al., 2012) also in the adult brain, increases mature OLG density, and selectively enhances myelination of active axons (Mitew et al., 2018). Notably, the mechanisms promoting OPC proliferation and/or differentiation depend on the pattern of neuronal activity (frequency and duration) of CAxs that differentially modulate vesicular Glu release on OPCs, which in turn can convert the different synaptic inputs into different intracellular events (Nagy et al., 2017). *In vivo* callosal stimulation at frequencies of 5, 25, and 300 Hz affected OPC proliferation and/or differentiation in diverse ways. Stimulation at 5 Hz was the most efficient frequency in triggering OPC differentiation, whereas 25 Hz was the most effective in promoting OPC proliferation (Nagy et al., 2017). Subsequent studies by the same group suggested that OPC proliferation and/or differentiation could also depend on the properties and subunit composition of AMPA_Rs_ at axon-OPC synapses. Modifications of AMPA_Rs_ containing the GluA2 subunit affect different functional properties at such synapses, since pore-dead GluA2 subunit mutation—by increasing AMPA_R_ Ca^2+^ permeability—enhances OPC proliferation and reduces their differentiation, whereas overexpression of the cytoplasmic C-terminal of GluA2 (C-tail) reduces OPC differentiation without affecting their proliferation. Therefore, Ca^2+^ permeability and AMPA_R_ subunit composition could play a key role in proliferation and/or differentiation mechanisms (Chen et al., 2018).

OPCs continue to proliferate and generate new OLGs throughout life (Young et al., 2013; Tripathi et al., 2017). It has been suggested that all OPCs are capable of division (Clarke et al., 2012; Young et al., 2013). The rate of OLG generation is high in the CC of young adult mice and diminishes with age (∼3.0 per mm^2^/day from P60 to P102; ∼1.5 per mm^2^/day from P60 to P380; and ∼0.4 per mm^2^/day from P240 to P380; Tripathi et al., 2017). Similarly, most human callosal OLGs form in the first 5–10 years of life and diminish with age (Yeung et al., 2014). These data are in line with the reduction in the cell division rate [cell cycle time (T_c_) at P21, ∼3 days vs. T_c_ at P60, ∼10 days; Young et al., 2013]. However, the rate of OLG generation is higher in the CC than in other fibrous systems. Moreover, a significant fraction of adult OLGs have an extended life since in the adult rat (P60), more than 90% of callosal myelinating OLGs are still alive at the age of 20 months, corresponding to a half-life (T_1/2_) greater than 10 years (Tripathi et al., 2017). Several studies have described NG2 glia, which continues to divide to generate new myelinating OLGs in the adult CC. Adult NG2^+^ cells expressing platelet-derived growth factor α receptor (PDGF α_R_), an OPC marker (Nishiyama et al., 1999), generate new myelin-forming OLGs in the CC and, to a lesser extent, in the cortical GM. Mapping studies of PDGFAR-Cre^ER^ transgenic mice have found that the new OLGs generated by callosal OPCs in adulthood exceed 20% of all OLGs generated in 90 days starting from P45 (Rivers et al., 2008). Slightly different results have been obtained between P60 and P120 using a different method of OLG identification and count. In the CC of P120 NG2-CreER™ transgenic mice, 30% of OLGs were generated by NG2 cells (Zhu et al., 2011). These data are consistent with those of an earlier radioautographic study, where intraperitoneal ^3^H-thymidine infusion showed that new OLGs continue to be generated in the CC of both young adult (4-month-old) and aged (9-month-old) male mice (McCarthy and Leblond, 1988). Similarly, a mouse study of the cell cycle dynamics of callosal NG2 cells (Psachoulia et al., 2009) has found that NG2 cells can divide until late in life and produce new myelinating OLGs up to 8 months of age. The above findings indicate that in the adult CC, OPCs continuously divide and differentiate into myelin-forming OLGs throughout life. Enriched environments (EE, consisting of a large cage containing wood shavings, toys, and small constructions, changed once a week) also affect callosal OLGs. Labeling of myelin-forming OLGs fromrats exposed to an EE or to a standard environment, with the myelin-associated enzyme 2′,3′-cyclic nucleotide 3′-phosphodiesterase (CNPase) demonstrated a 343% significant increase in CNPase^+^ cells in EE rats (Zhao et al., 2011). By contrast, social isolation, nursery rearing, and neglect impaired myelination, resulting in a reduced callosal area in both primates and humans (Sánchez et al., 1998; Teicher et al., 2004). There are several hypotheses on the roles of OPCs and new adult OLGs in the adult CC. Traumatic CAx injury in mouse causes a transient proliferative response of residing OPCs, which may contribute to remyelination by replacing dead OLGs (Flygt et al., 2017). OPCs may also be involved in replacing OLGs that degenerate during life or, alternatively, could give rise to remyelinating cells in demyelinated lesions. Indeed, studies of demyelinating disease models, such as toxicity induced by cuprizone (CPZ; a copper chelator; bis-cyclohexanone-oxaldihydrazone) in mouse CC have documented that remyelination requires OPC proliferation and differentiation into new OLGs, which go on to form the new myelin sheaths (Kuhn et al., 2019). New OLGs could also be involved in remyelination processes in adulthood and old age. In the mouse CC, myelination continues at a reduced rate (Sturrock, 1980; Richardson et al., 2011) until adulthood, when only 28% of CAxs are myelinated (Sturrock, 1980). Notably, EM investigations suggest that in the rodent CC, myelinated axons increase in adulthood (Nuñez et al., 2000; Yates and Juraska, 2007). In the genu of old rhesus monkeys, normal aging involves a 21% reduction in the number of myelinated nerve fibers *per* unit area, while the remaining nerve fibers show evident signs of myelin sheath degeneration, with splitting or ballooning of the sheath, which fills with fluid or dense cytoplasm. Simultaneous with sheath deterioration, CFs show evident albeit inadequate signs of remyelination, especially an increased frequency of paranodal profiles and redundant myelin sheaths (Peters and Sethares, 2002; Bowley et al., 2010). Adult-born OLGs might complete the myelination of callosal neurons with intermittent myelination or *de novo* myelinate naked axons during their lifetime (Young et al., 2013; Tomassy et al., 2014). Adult myelination is essential for learning motor skills. Production of newly formed callosal OLGs is accelerated in adult mice that are learning a new skill (running on a wheel with irregularly spaced rungs; McKenzie et al., 2014). Similar results have been obtained in humans. An MRI study of adult individuals who had practiced the piano during childhood and adolescence has found a positive correlation between practice and callosal myelination. A significant fractional anisotropy correlation was found in two callosal regions, the isthmus, which contains fibers connecting auditory regions in the superior temporal gyri, and the body, which contains fibers connecting the dorsal premotor cortices and the mesial premotor areas, which play key roles in bimanual coordination and learning of motor sequences (Bengtsson et al., 2005).

##### 3.1.1.6 Voltage-gated Na^+^ channels on callosal NG2 cells

In addition to Glu_Rs_, callosal NG2^+^ cells also express voltage-gated Na^+^ channels (Na_Vs_; Berger et al., 1992a; De Biase et al., 2010; Chittajallu et al., 2004; Káradóttir et al., 2008), which may underpin the ability of some NG2 cells to produce (atypical) APs (Chittajallu et al., 2004; Káradóttir et al., 2008; De Biase et al., 2010). There are conflicting reports on the presence of NG2 cells producing APs in the CC. Some studies have first described two classes of NG2 GCs, endowed with different properties, in the WM and CC. One group lacks Na_Vs_ and synaptic input, whereas the other receives synaptic input and expresses voltage-gated Na^+^ currents capable of generating APs. Both classes persist into adulthood after myelination is complete (Káradóttir et al., 2008). Yet subsequent studies have not found them. Cell voltage-clamp recordings performed in callosal slices of developing and mature mice have documented tetrodotoxin-sensitive currents, which are therefore mediated by Na_Vs_, in callosal NG2 cells (Berger et al., 1992a). Na_Vs_-mediated currents were recorded in both mature (P40–P45; 18/18 cells studied) and younger (P5–P8; P12–P15; P20–P26) mice, without age-related changes in current density. Moreover, in the first postnatal week (P5–P8) single, small, atypical Na-dependent spikes were evoked in some NG2 cells (4/7 cells) in response to an injection of depolarizing current. However, these spikes did not fulfill the criteria for APs, in that i) they exhibited a high activation threshold; ii) they did not overshoot 0 mV; and iii) their amplitude increased with larger current injections. These rudimentary APs were not found in adult callosal NG2 cells. Recordings performed in callosal NG2 cells of adult and younger mice indicated that these cells were incapable of generating APs, since injections of depolarizing current only induced changes in membrane potential (De Biase et al., 2010). Moreover, the transformation of NG2 cells into premyelinating OLGs resulted in Na_V_ downregulation (De Biase et al., 2010). Since Na_Vs_ do not generate APs, their role in the adult CC is still elusive. It has been hypothesized that they could execute a myelination program independent of transmitter release from axons (Káradóttir et al., 2008) or contribute to maintain cell proliferation and migration ability in the adult brain (De Biase et al., 2010).

##### 3.1.1.7 NG2 cells at the nodes of Ranvier

In the rodent CC, as in other fibrous tracts such as the optic nerve and spinal cord (Butt et al., 1999; Serwanski et al., 2017), ASs and processes of NG2 OPCs are found at the nodes of Ranvier (NoRs). In the rat CC, 95% of the NoRs contained AS processes, whereas 35% contained NG2^+^ cell processes. NG2 OPCs and AS processes showed different arrangements; although the former contacted the nodal membrane at discrete points, while the latter had broader processes surrounding the node (Serwanski et al., 2017). Although the significance of NG2 cells at the NoRs is still being investigated, their position would enable them to limit the lateral extension of OLGs in the late myelination stage, or to sense changes in myelin state, triggering, where needed, their own transformation into myelinating cells. NG2 cells synthesize matrix metalloproteinases and could be involved in tissue maintenance and remodeling at the NoR or at the paranodal junction (Serwanski et al., 2017).

##### 3.1.1.8 Metabolic support of OLGs

The myelin sheath and axons are a functional and mutually interactive, metabolically coupled unit. Their action is supported by lactate, delivered by OLGs. Studies of the optic nerve suggest that OLGs and ASs take up blood-derived glucose via the glucose transporter, GLT-1, for glycolysis. Coupling of ASs and OLGs through gap junctions allows transferring lactate, produced by glycolysis, to OLGs. Lactate from ASs and from OLG metabolism is moved to the periaxonal space through monocarboxylate transporter 1 (MCT1), which resides in the internodal myelin. Lactate can then be taken up by axons through MCT2 (located in the axonal compartment) and used to support axonal functions. During myelin biogenesis, OLGs may use AS-derived lactate as an energy source for mitochondrial ATP production or, more directly, to synthesize myelin lipids (Sánchez-Abarca et al., 2001; Rinholm et al., 2011; Funfschilling et al., 2012). In the periaxonal space, OLG MCT1 plays a key role in providing energy for myelination. *In vivo*, OLG-specific downregulation of MCT1 in both the CC and optic nerve has been seen to induce axon degeneration (Lee et al., 2012). The CC has a different energy supply from the optic nerve, as lactate is insufficient to sustain axonal functions. In acute brain slices of mouse CC, exogenous glucose deprivation efficiently abolished compound action potentials (CAPs), which were rescued by filling single OLGs with glucose; however, filling with lactate resulted in only partial rescue (Meyer et al., 2018). Notably, callosal OLGs are directly coupled with other OLGs by homotypic gap junctions, formed by oligodendrocytic connexins Cx47 and Cx32, rather than with ASs (Maglione et al., 2010). Filling OLGs lacking Cx47 with glucose failed to prevent CAP loss. This suggests that an OLG functional syncytium depending on Cx47 provides glucose to sustain axonal electrical activity (Meyer et al., 2018). The activation of NMDA receptors, expressed by OLGs in response to Glu release by axonal electrical activity, can play an important role in regulating glucose uptake, preventing abnormal lactate accumulation (Saab et al., 2016). Notably, Cx47 and Cx32 are critical for myelination since animals that lack both undergo dramatic CNS demyelination (Menichella et al., 2003). Moreover, as described in the optic nerve, Cx47 and Cx32, together with the neuronal K^+^ channel (Kir4.1) located in the axolemma, can play a key role also in the CC, buffering K^+^ ions during neuronal activity (Menichella et al., 2006). In the rat, each OLG ensures rapid AP conduction by sheathing ∼10 CAxs with myelin (Bakiri et al., 2011) and supports axon survival and function by supplying them with metabolic substrates.

##### 3.1.1.9 OLG membrane vesicle transfer

Axonal function and long-term integrity seem to be sustained by the transfer, from OLGs, of membrane vesicles in the form of exosomes besides glycolysis products. Exosomes are released into the periaxonal space, where they deliver specific myelin proteins such as proteolipid protein (PLP) and myelin-associated glycoprotein. Exosome secretion is closely related to the electrical activity of axons; indeed, electroactive axons release Glu (Kukely et al., 2007; Ziskin et al., 2007), which induces Ca^2+^ entry through oligodendroglial Glu receptors (AMPA/NMDA). The increased [Ca^2+^]_i_ triggers exosome release from OLGs along the internodes; once free, exosomes are internalized by axons (Frühbeis et al., 2013a; Frühbeis et al., 2013b). Some studies have suggested that the electrical activity of nerve fibers could influence the overlying myelin sheath. APs running through myelinated fibers open Ca_Vs_ in the internodal axon region. Axonal Ca^2+^ acts on gated ryanodine receptors present on axoplasmic reticulum membranes to release additional Ca^2+^ ions in order to promote vesicular fusion and Glu release in the periaxonal space. Glu activates myelin Glu receptors (AMPA/NMDA5), which dynamically modulate myelin physiology and structure also in the CC (Micu et al., 2016).

### 3.2 Astrocytes

The second most abundant GC type in the CC are fibrous ASs (Mori and Leblond, 1970). In the normal adult CC, ASs are continuously generated in the subventricular zone by nestin^+^ neural progenitor cells at a rate that declines with age, replacing apoptotic ones and keeping numbers constant. In mice, more than 10% of callosal ASs are replaced between 3 and 9 months of age (Sohn et al., 2015). GFAP, an intermediate filament protein expressed in ASs, is the typical AS marker in the CNS (Kimelberg and Nedergaard, 2010; Zhang et al., 2019). ASs immunolabeled with the GFAP antibody have a star or spider shape. Their processes emanate radially from the perikaryon, extending parallel or at an angle to the CFs. They are thin, poorly branched, and irregular and have a smooth surface and interdigitate with processes from neighboring ASs (Oberheim et al., 2009; Sun et al., 2010). In the bovine CC, a small number of elongated fibrous ASs exhibit processes that mainly originate from the two poles of the cell body and run parallel to the CFs (Matute et al., 1994a). GFAP^+^ ASs increase by 7% from P6 to P30. Notably, GC density varies among callosal regions. In the adult rat, GFAP^+^ AS density diminishes from the genu (1,824/μm^2^) to the splenium (1,788/μm^2^) and the body (1,405/μm^2^), possibly with relation to changes in callosal size and number of CAxs (Reyes-Haro et al., 2013). Immunocytochemical analysis of cultured ASs from newborn (5-days-old) rat CC and cerebral cortex has found that at least 95% of cells were GFAP^+^. Whereas in the cerebral cortex, ASs were endowed with large cytoplasmic regions with laminar processes and moderate GFAP immunoreactivity; callosal ASs exhibited a reduced cytoplasmic volume, smaller nuclei, longer and branched processes, and intense GFAP immunostaining (Goursaud et al., 2009). The presence of GFAP in ASs has been related to numerous functions. Several observations indicate that GFAP is essential for WM integrity; for instance, GFAP-deficient mice exhibit reduced myelin levels and CC thickness, whereas the WM shows reduced vascularization and structural and functional BBB impairment. These findings suggest that GFAP may be involved in the crosstalk between ASs, capillaries, and OLGs (Liedtke et al., 1996). GFAP also plays a key role in modulating astrocytic Glu_T_ function. In particular, GFAP acts as an intracellular protein anchoring glutamate aspartate transporter (GLAST) to the cytoskeleton. GLAST anchoring to the cytoskeleton is therefore an important element in Glu homeostasis (Sullivan et al., 2007). These findings are further supported by observations that suggest a reduction in Glu transport activity in the brains of GFAP-deficient mice (Hughes et al., 2004).

#### 3.2.1 Glutamate receptors in callosal astrocytes

Glu_Rs_ expression in callosal GCs has been studied in postnatal development and adulthood using various techniques (Table 4). The ontogeny of non-NMDA Glu_Rs_ has quantitatively been studied by *in vitro* receptor autoradiography of [^3^H] kainic acid binding sites. In the rat CC, binding was detected already at P1, it showed a transient increase in the second postnatal week (P14) and then a slight reduction in the third week, reaching the adult pattern at P60. This trend probably reflects concurrent callosal maturation (Miller et al., 1990). Non-NMDA receptors have also been detected in adult rat CC. The regional expression pattern of the five functionally identified high-affinity kainate receptor subunit genes has been investigated with oligonucleotide probes in adult rat brain sections. As in previous findings (Miller et al., 1990), the results indicate that high-affinity kainate receptor KA-1 mRNA (GluK4 according to the NC-IUPHAR nomenclature; see Collingridge et al., 2009) is widely expressed in the adult rat CC. Its activation seems to be common throughout the CNS and often leads to sustained Ca^2+^ oscillations (Wisden and Seeburg, 1993). In patch-clamp experiments, in frontal brain slices of the CC, glioblasts from 6- to 8-day-old mice expressed Glu responses similar to those of kainate receptors. The addition of NMDA to the bath solution did not cause current responses, indicating a lack of NMDA receptors (Berger et al., 1992a; Berger et al., 1992b). In addition to kainate, callosal glial precursor cells express AMPA-type Glu_Rs_. Precursor cells, identified by their voltage-gated current, were easily discernible in ASs and OLGs. Kainate induces two effects: activation of a cationic current and block of K^+^ conductance. These effects are abolished by 6,7-dinitroquinoxaline-2,3-dione (DNQX), a non-selective AMPA/kainate receptor antagonist. Because K^+^ currents are critical for the proliferation of several cell types, AMPA/kainate receptor activation may be the first step leading to the arrest of proliferative processes. The presence of these receptors in precursor cells could have an important role in AS and OLG differentiation (Berger, 1995). Electrophysiological techniques and *Xenopus* oocytes have been employed to study the expression of neurotransmitter receptors encoded by mRNA extracted from human and bovine CC. Oocytes injected with mRNA from the CC of these species expressed functional receptors to Glu and to other neurotransmitters (ACh, serotonin), as well as Ca^2+^ channels. Glu and its analogs, NMDA, kainate, and AMPA, elicited small, smooth inward currents. Non-NMDA receptors showed a strong rectification and were blocked by DNQX, as seen in the case of the AMPA/kainate Glu_Rs_. Moreover, the electrophysiological properties of the AMPA receptors expressed by callosal mRNA suggest that they are molecularly related to Glu_R1_ (NC-IUPHAR nomenclature, GluA1; Collingridge et al., 2009) and Glu_R3_ (NC-IUPHAR nomenclature, GluA3) receptor subtypes. *In situ* hybridization experiments combined with GFAP ICC suggest that Glu_R1_ and Glu_R3_ are expressed by type-2 ASs (Matute and Miledi, 1993). An RT-PCR study has demonstrated mRNAs encoding kainate-preferring Glu_R_ subunits Glu_R5-7_ (NC-IUPHAR nomenclature, GluK1-3), KA-1 (NC-IUPHAR nomenclature, GluK4), and KA-2 (NC-IUPHAR nomenclature, GluK5) in the adult bovine CC. Double-labeling experiments disclosed Glu_R5-7_ (GluK1-3) and KA-2 (GluK5) in ∼50% of GFAP^+^ ASs. Moreover, callosal ASs co-expressed Glu_R5-7_ and KA-2 subunits, whereas ASs expressing kainate subunit Glu_R5-7_ expressed the AMPA receptor subunit Glu_R1_ (García-Barcina and Matute, 1996). In an RT-PCR and ICC study, García-Barcina and Matute (1998) investigated the presence of AMPA Glu_Rs_ in the adult bovine CC. Consistent with previous work, they found that Glu_R1_ and Glu_R3_ are both expressed by fibrous ASs. Glu_R1_ was particularly abundant in astroglial processes surrounding capillaries, where they formed intensely labeled end-feet (Matute et al., 1994a). In addition to the ionotropic receptors (iGluR_s_), callosal ASs also express mGluR_s_. Cultured ASs from rat CC express mGlu_R5_. Their density is particularly high, and immunoblot analysis has shown that mGlu_R5_ is 7.7 times denser in WM (CC) than in GM and that CC ASs show a high density of GFAP, vimentin, and nestin (Goursaud et al., 2009).

**TABLE 4:** Receptors in callosal astrocytes.

Glutamate receptors (Glu_Rs_)	Species (age)	Techniques	Types, localization, presumed function	References
Ionotropic (non-NMDA)				
Kainate	Rats (P3–P13)	Electrophysiology	Block of K^+^ conductance. Regulation of AS proliferation	Berger (1995)
	Rats (P1–P28–adult)	Autoradiographic localization of [^3^H] kainic acid binding	Transient increase during the second postnatal week. Cellular mechanism mediating neuronal morphology regulation	Miller et al. (1990)
	Humans. Bovine	ICC, electrophysiology, *in situ* hybridization	Bovine and human CC possess mRNAs coding for Glu. AMPA/kainate Glu_Rs_ (Glu_R1_ and Glu_R3_) show a strong rectification at positive potentials, Glu_R1_ and Glu_R3_ cRNA probes hybridize predominantly with a subpopulation of GFAP-positive cells: type-2 ASs. Neurotransmitter receptors in ASs may be activated by neurotransmitters released from axons, which in turn may induce cytokine and growth factor release. Such interactions may be involved in processes such as preservation of axon–glia integrity or guidance of axons during injury repair	Matute and Miledi (1993)
	Rats (adult)	*In situ* hybridization	KA-1 type autoradiographic signal (KA-1 mRNA) was the most noticeable in the CC and was transiently expressed, showing a developmental binding peak 2 weeks after birth	Wisden and Seeburg (1993), Miller et al. (1990)
	Bovine	RT-PCR, ICC	KA-2, Glu_R5-6-7_. Widespread distribution. Glu_R5-7_ and KA-2 subunits and Glu_R6_ and AMPA receptors are co-expressed. Important in axon-to-glia and/or glia-to-glia signaling	García Barcina and Matute (1996)
AMPA	Bovine	RT-PCR, ICC, Western blotting, cell cultures	Glu_R1-2-3-4c_ are abundantly expressed in the CC. In particular, Glu_R1_, located in the AS cell body and radial processes, contacts blood vessels, forms intensely labeled end-feet, participates in regulating BBB permeability, and controls new vessel formation. The functional significance of the other AMPA Glu_Rs_ *in situ* remains to be elucidated	Matute et al. (1994), García Barcina and Matute (1998)
Metabotropic Glu_Rs_				
mGlu_R5_	Rats (P5–adult)	Callosal AS cultures, immunoblotting	Immunoblot analysis revealed high mGlu_R5_ expression in callosal ASs (7.7 times denser in the CC than in GM). mGlu_R5_ positively modulates GLT expression and function. The Glu–mGlu_R5_ interaction triggers a chain of events that leads to PGE2 activation, hence vasodilation, and to AQP-4 phosphorylation to control AS water permeability	Goursaud et al. (2009), Hall et al. (2014), Sweeney et al. (2016), Atwell et al. (2010), Hamilton et al. (2010), Gunnarson et al. (2008), Iacovetta et al. (2012)
Angiotensin receptors				
AT_1_, AT_2_	Bovine. Humans. Rats (3-week-old)	Northern blotting, *in situ* hybridization, Western blotting, AS cultures	The function of Ang II receptor in the CC is still elusive, but the presence of densely stained ASs in close contact with callosal microvessels suggests that they may regulate callosal blood flow and fluid homeostasis through the release of prostaglandins and aldosterone	Matute et al. (1994b), Montiel-Herrera et al. (2006)
Purinergic receptors				
Metabotropic P_2y1_	Bovine (adult)	Northern blotting, RT-PCR	Astrocytic P_2y1_ activation increases cytosolic Ca^2+^, which in turn can trigger several cascades of events in GCs, such as the generation of propagating Ca^2+^ waves in ASs and other GCs. Astrocytic P_2y1_ participates in various neuron–glia and glia–glia signaling processes	Deng et al. (1998), Schipke et al. (2002)

#### 3.2.2 Glutamate transporters in callosal astrocytes

ASs are involved in numerous functions such as the regulation of neurotransmitter concentrations in the extracellular space. The physiological level of extracellular Glu in the CC and other CNS regions is ensured by Na^+^-dependent Glu_Ts_, a group of proteins found in cytoplasmic membranes. Several Glu_Ts_ have been cloned: GLT-1 (or excitatory amino acid transporter 2, EAAT2), GLAST (EAAT1), excitatory amino acid carrier-1 (EAAC1 or EAAT3), EAAT4, and EAAT5 (Matute et al., 2007a). The first report of Glu_Ts_ in the CC dates back from 1992 (Danbolt et al., 1992). Polyclonal antibodies raised against the purified protein were used to study GLT immunolocalization by using LM and EM. Stained astroglial-like elements were detected in rat neocortical GM and WM, including the CC. LM and EM both indicated that GLT labeling tended to be more intense in glial-like processes than in AS cell bodies. A subsequent immunohistochemical study by the same group using antibodies against synthetic peptides demonstrated that two Glu_Ts_, GLAST and GLT-1, are expressed by the same astrocytic cell bodies and processes (Lehre et al., 1995), whereas an *in situ* hybridization histochemical study documented EAAC1 in OLGs in the rat CC (Kiryu et al., 1995). Further immunocytochemical and Western blot (WB) experiments (Domercq and Matute, 1999) have investigated the phenotype of the cells expressing Glu_Ts_ in the adult bovine CC using EAAC1, GLAST, and GLT-1 antibodies. EAAC1 expression was detected in a small population of immature oligodendroglial cells, probably adult OPCs (Domercq and Matute, 1999). Unlike the rat CC, where GLAST is expressed only by ASs (Lehre et al., 1995), in bovine CC, it was detected in 71% of cells of a particular class of I-OLGs. These cells, which have a small, round body without visible processes (at least with this technique; see below; Tanaka et al., 2021) clustered in short rows parallel to axon fibers (Domercq and Matute, 1999). GLAST was also observed in a small population of cells with the typical star shape of ASs (Domercq and Matute, 1999). GLT-1-expressing cells had a star morphology and were always GFAP^+^ ASs (Domercq and Matute, 1999). The maintenance of Glu homeostasis in synapsis-poor brain regions, like the CC, may be critical to prevent oligodendroglial and axonal damage and to ensure normal axonal electrical activity. Indeed, inhibition of Glu uptake in another axonal tract, i.e., the optic nerve, has been reported to increase Glu levels and to induce OLG excitotoxicity and massive demyelination (Domercq et al., 2005; Matute et al., 2007a). In addition to ensuring Glu homeostasis in the CC, Glu taken up into ASs can be metabolized and converted to glutamine, a process catalyzed by the astrocytic enzyme glutamine synthetase (Norenberg and Martinez-Hernandez, 1979). In turn, glutamine is released into the extracellular space by an electroneutral Na^+^-dependent transporter (SN1; Chaudhry et al., 1999; Bröer and Brookes, 2001). Glutamine is then probably taken up by ICNs (see below), by a system A transport (SAT/ATA; Bröer and Brookes, 2001). Once inside the neurons, glutamine is converted by phosphate-activated glutaminase (PAG) to Glu which is then used to fill synaptic vesicles or is converted to α-ketoglutarate to enter the tricarboxylic acid cycle (Magi et al., 2019). *In vivo* enzymatic analysis has revealed that callosal GS and PAG activities are 11%–25% and 25%, respectively, of those in the cerebral cortex (Hassel et al., 2003). By contrast, in an immunoblotting study of cortical and callosal astroglial cultures, GS expression was 1.8-fold higher in WM ASs than in GM ASs (Goursaud et al., 2009).

#### 3.2.3 Astrocytic control of capillary blood vessels and extracellular homeostasis

Fibrous ASs contact blood capillaries through their end-feet and are therefore BBB constituents. The other elements making up the BBB are GCs in the wall of blood vessels and the basal lamina, located between the endothelial cells (ECs) and astrocytic end-feet (Peters et al., 1991). Enclosed in the endothelial basal lamina are pericytes, which express a contractile protein (α-smooth muscle actin; Bandopadhyay et al., 2001). ECs and pericytes are connected to the basal lamina by several types of integrin molecules; in areas lacking the basal membrane, they interdigitate with each other, forming direct connections that contain several different transmembrane junctional proteins. These BBB constituents are also connected by pairs of Cx43 hemichannels. Different types of tight junction proteins connect ECs to one another; moreover, Cx43 forms gap junctions at the AS–endothelial cell (AS-EC) interface (Winkler et al., 2011). In the rat CC, the BBB is fully formed by the second postnatal week. Studies of its ultrastructure in the developing CC indicate that blood vessels of 1-day-old rats are wholly devoid of astrocytic processes, whereas ECs show tight junctions and an ill-defined basal lamina. In 7-day-old rats, ECs are partially covered by astrocytic end-feet. At 14 days, the wall of the callosal capillaries is well developed; the basal lamina is denser and well defined, and is surrounded by a continuous sheath of astrocytic end-feet (Xu and Ling, 1994). In the adult rat CC, GFAP^+^ astrocytic processes completely surround the blood vessels. The cytoplasm and processes of perivascular ASs are thick and abundant compared with those of the cerebral cortex; in addition, the vascular walls of the callosal vessels are characterized by perivascular glial rows and cylindrical segmented astrocytic terminations (as noted above, GFAP expression is essential for BBB integrity; see Liedtke et al., 1996). EM observations have documented that astrocytic processes continuously cover the outer vascular wall of callosal blood vessels and are thicker than those found in the cerebral cortex (Suzuki et al., 2003). These findings suggest that in the CC, a full-fledged BBB exists from P14 onward; moreover, in the adult CC, each AS has a wide contact area with the outer wall of microvessels (Xu and Ling, 1994; Suzuki et al., 2003). A similar anatomical organization has also been described by ICC (Matute et al., 1994a). Glu_R1_, an (AMPA)/kainate receptor subtype, was particularly abundant in the end-feet and glial fibers surrounding callosal capillaries. In a study of cultured GCs, ASs exposed to Glu sprouted numerous filopodia from the cell surface in a receptor-mediated process activated by kainate (Cornell-Bell et al., 1990). Interestingly, Glu may play an important role in regulating cerebral blood flow and much of this control may be mediated by AS-pericyte interactions (Atwell et al., 2010; Hamilton et al., 2010; Hall et al., 2014; Sweeney et al., 2016). Glu released at arbitrary sites along CFs (Kukley et al., 2007; Ziskin et al., 2007) can diffuse in all directions into the neighboring space (Vizi et al., 2010) and reach extrasynaptic mGlu_R5_ in callosal distal astrocytic processes (Goursaud et al., 2009; Panatier and Robitaille, 2016). mGlu_R5_ can also be reached by Glu released at axon–glia synaptic junctions (see above; Kukley et al., 2007; Ziskin et al., 2007) by a “spillover” mechanism (Vizi et al., 2010). Glu–mGlu_R5_ interaction induces a [Ca^2+^]_I_ increase in ASs, in turn activating phospholipase A_2_ followed by the production of arachnoid acid from membrane phospholipids. Arachnoid acid is then metabolized to prostaglandin E_2_ via cyclo-oxygenase-1. PGE_2_ from ASs binds prostaglandin EP4 receptor, which dilates the capillaries by activating an outward K^+^ current in pericytes; this chain of events ultimately increases the blood flow (Atwell et al., 2010; Hamilton et al., 2010; Hall et al., 2014; Sweeney et al., 2016). Pericyte relaxation is also mediated by NO. In the CC, Glu acting on ICNs is capable of releasing NO (Sancesario et al., 1996; Barbaresi et al., 2014; 2020; Rockland and Nayyar, 2012; Sagrati et al., 2023), which in turn inhibits cytochrome P450 in ASs and cytochrome P450 4A in pericytes, preventing the conversion of arachnoid acid to epoxyeicosatrienoic acid and the vasoconstricting agent 20-hydroxyeicosatetraenoic acid, respectively (Atwell et al., 2010; Hamilton et al., 2010; Hall et al., 2014; Sweeney et al., 2016). In addition, ASs are strategically placed to control ion and water homeostasis in the interstitial space. In the CC, astrocytic end-feet are rich in the water channel protein aquaporin-4 (AQP-4), which points at a key role of ASs in regulating brain water permeability (Sohn et al., 2015). There is strong evidence that Glu heightens water permeability of ASs expressing AQP-4. Glu binds to group I mGlu_R5_ on ASs, triggering the release of [Ca^2+^]_i_ stores, NO production, and activation of protein kinase C (PKG) –mediated phosphorylation of the AQP-4-serine^111^ residue on loop B, which is found in a strategic position to control water channel gating (Gunnarson et al., 2008; Iacovetta et al., 2012). In the CC, AQP-4 co-localizes with the astroglia-specific excitatory amino acid transporter GLAST. Although several types of inwardly rectifying K^+^ channels have been detected in cultured callosal ASs by electrophysiological investigations (Reyes-Haro et al., 2005; Montiel-Herrera and García-Colunga, 2010), there are no data on the presence on callosal astrocytic end-feet of the inward rectifier potassium channel (Kir 4.1). In other CNS regions, Kir 4.1 has been proposed as a molecular partner of AQP-4 in spatial buffering of K^+^, facilitating water movement through the plasma membrane (Benfenati and Ferroni, 2010; Nagelhus and Ottersen, 2013). These findings indicate that in the CC, as in other CNS regions, astrocytic AQP-4 may be the molecular target for Glu-mediated regulation of AS water permeability.

#### 3.2.4 Metabolic support of astrocytes

ASs directly contribute to the metabolic support of CAxs. As noted above, 95% of CF NoRs contain AS processes (Serwanski et al., 2017). From this position, ASs can easily supply lactate, derived from glycogen, to CAxs through MCT1/4 astrocytic transporters. In turn, axons can take up glycogen-derived lactate through the MCT2 transporter and use it to fuel mitochondrial ATP synthesis in response to low energy levels and/or during high-discharge frequency of impulse transmission (Wender et al., 2000; Brown et al., 2004; Tekkök et al., 2005; Saab et al., 2013). Both monocarboxylate transporters are widely expressed in the CC. RT-PCR and WB analysis have shown that in the rat CC, MCT1 gene and protein are consistently expressed from birth to adult age. Moreover, a confocal microscopy investigation involving double labeling with the GFAP glial marker has found that at P0, 70% of MCT1^+^ cells were also GFAP^+^; the proportion of double-labeled cells then began to decline to 30% at P14 and to ∼19% at P21, when it plateaued until adulthood (Dong et al., 2017). MCT4 expression is very low at birth and reaches adult level at P14. Indeed, a peroxidase IHC study of rat CC (Rafiki et al., 2003) found some weakly MCT4+ astroglial cells at P7 and a larger number of more intensely stained cells at P14. A similar pattern was observed in the adult CC. It has been speculated that in the early postnatal period, when the glucose transporters are not yet fully mature, ASs in direct contact with the blood vessels take up lactate directly from the blood through MCT1/MCT2, expressed on ECs (Rinholm et al., 2011) and astrocytic end-feet, and export it to axons/neurons; at later development stages and in adults, axons/neurons would predominantly be fueled by lactate from glucose metabolism through MCT4 (Rafiki et al., 2003; Dong et al., 2017).

#### 3.2.5 Renin–angiotensin system

The renin–angiotensin system is one of the developmentally oldest hormone systems (Table 4). It plays an important role in maintaining extracellular volume and in regulating arterial pressure, vasoconstriction, sodium intake, and potassium excretion. Its chief effector peptide is angiotensin II (Ang II), an octapeptide derived from angiotensinogen through sequential enzymatic cleavages by renin and angiotensin converting enzyme. Ang II acts through activation of two pharmacologically distinct G protein–coupled receptors, Ang II type 1 (AT_1_) and 2 (AT_2_), which usually induce opposite effects. The receptors are found in several body tissues including many CNS regions (Phillips et al., 1993; Reagan et al., 1994; Jackson et al., 2018). Northern blot analysis and *in situ* hybridization performed in adult bovine and human CC sections and in GC cultures have demonstrated that Ang II receptors are molecularly related to astrocytic AT_1_ type (Matute et al., 1994b). WB analysis of protein extracts from bovine CC and IHC performed in slices of bovine and human CC using AT_1_ antibody have confirmed earlier data that demonstrated AT_1_ receptor in the CC. Indeed, numerous densely stained ASs, several of which were in close contact with microvessels, were detected throughout the CC, as were numerous, moderately labeled cells with a round morphology, interpreted as I-OLGs (Fogarty and Matute, 2001). At variance with these studies, a recent article has described Ang II responses mediated by AT_2_ receptor activation in cultured callosal ASs from newborn and 3-week-old rats (Montiel-Herrera et al., 2006). The discrepancy is probably due to methodological issues such as subject age and species, since cultured cells can change characteristics over time. The function of Ang II receptors in the CC is still elusive. CC experiments suggest that glial Ang II receptor activation leads to secretion of prostaglandins (PGs) and aldosterone. PGs released near callosal blood vessels can have an important role in regulating cerebral blood flow and influence neuronal K^+^ channel activity. Notably, aldosterone increases the expression of (Na, K)-ATPase alpha3 and beta1 subunits by influencing the activity of neurons involved in body fluid homeostasis (Matute et al., 1994b; Fogarty and Matute, 2001).

### 3.3 Microglia

#### 3.3.1 Classification and morphology of callosal microglial cells

In addition to the two glial elements described above, the callosal parenchyma also contains microglia, i.e., the ubiquitous CNS immune cells (Kettenmann et al., 2013). In the cerebral cortex, their distribution varies in the different cytoarchitectonic areas. In the CC, microglia comprise up to 5% of the glial population (Mori and Leblond, 1969; Mori and Leblond, 1970; Lawson et al., 1990). Microglia have a heterogeneous morphology in relation to their location. Three morphological types have been described: i) compact cells in the circumventricular organs, characterized by a small (<150 μm^2^) round soma and three short processes; ii) longitudinally branched cells in fiber tracts, characterized by an elongated cell body (175–225 μm^2^) and long processes running parallel to surrounding axons; and iii) radially branched cells in all GM areas, having the largest (>275 μm^2^) round or slightly elongated cell bodies from which originate 3–5 principal branches (Lawson et al., 1990). These processes are very active and dynamic even in the resting condition (resting microglia). Microglial cells play an important surveillance role in the brain environment. In response to any type of brain injury or pathological event capable of altering CNS homeostasis, they undergo changes in morphology, behavior, and gene expression, turning into active microglia with a high phagocytic capacity (Nimmerjahn et al., 2005; Soulet and Rivest, 2008; Kettenmann et al., 2011; Gundersen et al., 2015). Early investigations have identified two types of microglia in rat CC. Pericytal microglia, found in the expansion of the basement membranes of capillaries, usually have a thin cytoplasm, an elongated nucleus, and few, short processes departing from the soma. Interstitial microglia are scattered throughout the CC. They have a small soma, an elongated nucleus, and a thin ring of cytoplasm containing many dense bodies (presumed to be lysosomes); processes are long and numerous with few branches (Mori and Leblond, 1969).

#### 3.3.2 Origin of callosal microglial cells

Injection of ^3^H-thymidine, which depicts proliferating cells, did not evidence pericytal or interstitial microglia, suggesting that they do not proliferate in normal conditions and that apoptotic cells are continuously replaced by fresh cells possibly coming from the circulation (Mori and Leblond, 1969). Indeed, EM investigations in young rat CC (Ling and Tan, 1974) have described ameboid microglial cells (AMCs), which at P5 accounted for 6% of the total glial population and by P15 had disappeared. By contrast, microglia, which were not detected at P5, increased with age, reaching 6% of the total glial population at P15. These findings have prompted the hypothesis that microglia derive from AMCs. Notably, AMCs have an intimate structural association with blood capillaries, suggesting an origin from blood. Further evidence for the notion has comes from Imamoto and Leblond (1978), who have confirmed earlier reports (Ling and Tan, 1974) that callosal AMCs disappear as microglial cells appear. Moreover, EM observations have highlighted that AMCs accounted for ∼6% of the glial population and showed various stages of transition from monocytes. The origin of callosal microglia has also been studied by injecting colloidal carbon into the rat circulation. Carbon-labeled monocytes were observed first in the lumen of callosal vessels, then in perivascular regions, and later at some distance from callosal vessels. After a few days, carbon-labeled AMCs and, subsequently, microglia were detected in the developing CC (Ling, 1979). In further experiments, Ling et al. (1980) injected carbon-labeled monocytes from donor Lewis rats into the circulation of 2- to 3-day-old syngeneic rats. They detected labeled monocytes in the CC that evolved to carbon-labeled macrophagic AMCs, which in turn disappeared after the first postnatal week, ultimately to transform into carbon-labeled ramified microglia. Taken together, these studies strongly support the hypothesis that monocytes, which enter the CC through the wall of local blood vessels and give rise to AMCs, are the precursors of ramified microglia (Ling et al., 1980). However, an origin from other sources, such as the transient cavity of the septum pellucidum or subependymal cysts, cannot be excluded. Several studies have shown that these structures serve as depots for monocyte-derived AMCs before they are dispatched to their respective sites (for a review, see Ling and Wong, 1993). The transformation of AMCs into ramified microglia is coincident with the onset of CAx myelination. Indeed, in the early postnatal days, AMCs are surrounded by a wide interstitial space—as CAxs are still unmyelinated, have a round morphology, have short, stout processes, and contain abundant cytoplasm with numerous lysosomal granules, several vacuoles, and some phagosomes. The transformation into ramified microglia, which have an elongated soma with slender processes, occurs in the second postnatal week, when the extracellular space has shrunk as a result of axon myelination. These data suggest that in the first 15 days of postnatal life, AMCs gradually lose their phagocytic ability and turn into a resting form of microglia (Kaur et al., 1985; Wu et al., 1992; Wu et al., 1994; Ling et al., 2001).

#### 3.3.3 Functions of callosal microglial cells

The presence of AMCs in the rodent and cat CC in the first postnatal weeks, shortly before the onset of myelination, has been related to the profound changes that remodel the commissure during this development phase. At this time, an excessive number of neurons send axons to the contralateral hemisphere through the CC (Innocenti et al., 1977; Innocenti and Caminiti, 1980). CAx overproduction, which is partly due to axon branching (Kadhim et al., 1993), is transient, and axon numbers eventually decline as they degenerate and are cleared by phagocytosis (Ling, 1976; Ling et al., 1980; O’Leary et al., 1981; Innocenti et al., 1983a; b; Kadhim et al., 1993). Electron micrographs of transient CAxs have revealed a sequence of degenerative morphological changes such as autophagic vacuoles, dark inclusions, and swollen profiles. Additionally, they are often surrounded by AMCs displaying clear signs of phagocytic activity, with small and large dense bodies and dark phagosomes containing cellular elements and bundles of unmyelinated nerve fibers (O’Leary et al., 1981; Innocenti et al., 1983a; Innocenti et al., 1983b; Ling, 1976; Ling et al., 1980). The role of AMCs in axon clearance has also been documented by injecting HRP into the contralateral hemisphere, which demonstrated bundles of anterogradely labeled transient CAxs on the AMC surface. Often, invaginations closed around the axons, forming vacuoles that contained electron-dense HRP precipitate. HRP labeling was absent in rats that had received the injection after callosotomy (Innocenti et al., 1983a; Innocenti et al., 1983b). AMCs also have an important role as a functional barrier, especially in the early postnatal period, when the BBB is not fully developed. In young rats, HRP injection into the circulation demonstrated callosal AMCs positive for HRP coming from callosal blood vessels. These data suggest that AMCs provide an effective barrier system against any foreign substances which may enter the brain parenchyma (Kaur et al., 1986). The microglial population shows a slow turnover throughout life. Its replacement may be related to its ability to divide, besides its recruitment from the circulating monocyte pool. A study combining a macrophage-specific antibody, F4/80, and ^3^H-thymidine injections detected numerous double-labeled cells, indicating that resident microglia can synthesize DNA and go on to divide *in situ* (Lawson et al., 1992). At slight variance with the above studies, cell counts have indicated that the relative contribution of immigrating monocytes ranged from 45% to 53%. In addition to their function in clearing callosal debris (Neuman et al., 2009), microglial cells promote myelin production. Evidence for the notion comes from OLG-microglia co-cultures showing that microglia stimulate the synthesis of sulfatide, a myelin-specific galactolipid, and the expression in OLGs of two myelin-specific proteins, myelin basic protein (MBP) and PLP. Moreover, EM showed numerous myelin-like membranes with a typical multilayered structure in cultured OLGs in the presence of microglia. These data suggest that a soluble microglial factor exerts a paracrine action that is sufficient to stimulate OLGs (Hamilton and Rome, 1994). Insulin-like growth factor 1 (IGF-1) has also been shown to play a major role in myelination. In the early postnatal brain, a subset of callosal microglial cells immunopositive for CD11c, an integrin heterodimer protein, express the *Igf-1* gene, which is critical for neurodevelopment and myelination. These cells are the chief source of IGF-1; *Igf-1* gene depletion in CD11c^+^ microglia results in reduced brain weight and a higher frequency of less myelinated CFs (Wlodarczyk et al., 2017). Brain macrophages also influence CAx elongation by secreting thrombospondin (TPS), an extracellular matrix protein. From the late embryonic stage to the end of the second postnatal week, clusters of TPS^+^ brain macrophages are detected in the rat CC. These cells have been related to the developmental changes involving the CC between P0 and P15, when several excess CFs are cleared (see above), whereas others reach their final destination (Ivy and Killackey, 1981; Innocenti, 1994). Since TPS^+^ macrophages fulfill the spatiotemporal conditions for stimulating axon growth in subcortical fiber tracts, they can favor neurite growth in those CAxs that are maintained (Chamak et al., 1995). Microglia are also involved in postnatal CF fasciculation. Two complementary approaches, pharmacological and genetic, have been harnessed to study this aspect of callosal development, i.e., induction of maternal inflammation by peritoneal injection of lipopolysaccharide (LPS) in pregnant dams and the use of embryos with a DAP12 mutation to examine the consequences of a loss of function of DAP12, a microglia-specific membrane protein required for normal phagocytosis. The two treatments downregulated the genes involved in neurite formation during development and induced dorsal CAx defasciculation (Pont-Lezica, et al., 2014). In addition to promoting myelination, microglia also exert an important homeostatic function, maintaining the number of OLGs that are involved in normal myelin formation during postnatal callosal development. A mouse study using fractalkine receptor (CX3CR1)–deficient AMCs has demonstrated a reduction in OPC engulfment that resulted in i) an OLG surplus, ii) an unchanged axon number and increased OLG/axon ratio, and iii) impaired myelin sheath formation with a reduction in myelin thickness in the adult (Nemes-Baran et al., 2020). Altogether, these experiments demonstrated that callosal microglia have important functions such as clearing excess axons, promoting axon growth, facilitating myelination, and guiding axonal pathways.

#### 3.3.4 Receptor expression on microglial cells

A wide range of experimental techniques have shown that microglial cells express a remarkable variety of receptors for neurotransmitters, neuropeptides, and neuromodulators and thus have the capacity to sense neuronal activity (Pocock and Kettenmann, 2007; Kettenmann et al., 2011; Garaschuck and Verkhratsky, 2019) (Table 5). Microglia express ionotropic and metabotropic receptors for Glu, ATP, adenosine, GABA, ACh, adrenaline, noradrenaline, serotonin, and histamine.

**TABLE 5:** Microglia receptors in the CC.

Receptor type	Species (age)	Techniques	Types, localization, function	References
Ionotropic Glu_Rs_				
Glu_R4_ subunit	Rats (neonatal, immature, adult). Bovine (adult)	ICC, double labeling, EM	All Glu_R4+_ were double-labeled with OX42, an AMC marker. Glu_R4_-AMCs were few in P1–P2 rats, they increased at P7–P10, and were rare or absent in P14 and adult rats. They were rare in adult bovine CC. The function of Glu_R4_ in callosal AMCs is unknown	Ong et al. (1996), García-Barcina and Matute, (1998)
GABA_Rs_				
GABA_A_ subunit	Mice (P6–P8). Male and pregnant female rats	Slice preparations, cell cultures, electrophysiology Immunofluorescence, Western blotting, EM, stereotaxic injection of lysolecithin	Recordings revealed that the GABA_A_ receptor agonist muscimol induced an increase in [K^+^]_o_ that in turn stimulated the release of chemokine MIP1-α. Results suggest that GABA-triggered MIP1-α controls some properties of microglia, ASs, and precursor cells in the developing CC. Remyelination processes in response to focal callosal demyelination have been studied in late pregnant, virgin, and postpartum rats. Remyelination in response to gliotoxin-induced demyelination in the CC was enhanced in late pregnant rats compared to virgin and postpartum rats and was lost when GABA_AR_ was blocked. These data suggest that pregnancy-associated promyelination operates through a GABAergic activated system	Cheung et al. (2009), Kettenmann et al. (2011), Kalakh and Mouihate (2019)
Notch_Rs_				
Notch-1	Rats (P1, P3, P7, P10, P14)	Western blotting, ICC, double labeling, glial cell cultures, RT-PCR	Notch-1 receptor expression was downregulated with age. It was very intense until P10, when it began to decline. Notch-1-immunopositive cells were confirmed to be microglia by OX42 labeling and colocalized with its ligands, Jagged-1 and Delta-1. Intraperitoneal injection of LPS increased Notch-1-positive AMC number and immunoreactivity in P3 rats, while Notch-1 neutralization reduced the expression of several cytokines and of iNOS. This suggests that AMC-Notch-1 regulates the expression on iNOS and of several cytokines	Cao et al. (2008)
Purinergic receptors				
Subtypes P_2x_, P_2z_	Mice (P5–P7)	Slice preparations, electrophysiology (patch-clamp technique)	Extracellular application of ATP generates a complex transmembrane cationic current with three components: fast, steady-state, and delayed inward currents. External application of the P_2_ purinoreceptor antagonist suramin significantly reduced the first two components of the ATP-induced currents and completely blocked the third; application of the tetra-anionic form of ATP (ATP^4-^) lowered the activation threshold of	Haas et al. (2006), Kettenmann et al. (2011)
Purinergic receptors				
			the delayed component, while reduction of ATP^4-^ concentration resulted in its disappearance. The first two components are mediated by P_2y_ purinoreceptors, the third by P_2z_. Since ATP appears in the CNS following cell damage, the activation of purinergic receptors could be a signal transduction mechanism triggering functional changes in microglia in response to brain injury	
Generic purinergic receptors, probably P	Mice (– P8)	Slice preparations, electrophysiology (patch-clamp technique)	Local ATP ejection elicited an astrocytic Ca^2+^ wave that spread over hundreds of micrometers. The wave spreads via ATP release and concomitant purinergic receptor activation. Activation of microglia purinergic receptors triggers a K^+^ outward current and cytokine secretion. Therefore, Ca^2+^ waves are not restricted to ASs. Microglial cells can sense the activation of other GCs	Schipke et al. (2002)
P_2x_, P_2y_	Mice (newborn, P6, and P9)	Microglial cell cultures	ATP application causes a transient increase in [Ca^2+^]i due to ATP interaction with P_2x_ receptors. After removing extracellular Ca^2+^ and applying ATP, it was still possible to record a transient [Ca^2+^]i due to the presence of a P_2y_ receptor capable of mobilizing Ca^2+^ from the ER	Möeller et al. (2000)
P_2X4_, P_2X7_, and P_2Y12_	Rats (P0–P7; P14)	Microglial cell cultures, ICC, double labeling, Western blotting	All three receptors were detected in rat CC. P_2X4_ was specifically localized in callosal AMCs in P0–P7 rats and showed the most intense expression. Labeling progressively diminished with age (P14). P_2X4_ expression was enhanced in P0 rats subjected to hypoxia. In hypoxic rats, IL-1β and TNF-α expression was increased, induced by ATP AMCs activation through P_2X4_ receptors. The prevalent ATP receptor P_2X4_ may be linked to regulation of AMC activation for production of proinflammatory cytokines, especially in hypoxia	Li et al. (2011)
Endothelin (ET-1, ET-2, ET-3)				
ET_Rs_ (ET_A_, ET_B_)	Rats (embryonic day 18 and 20; P0–P14)	ICC, immunoelectron microscopy, immunofluorescence, double labeling, microglial cell cultures, RT-PCR	ET was specifically localized in AMCs, as shown by double immunofluorescence with isolectin B4, a marker for microglia. ET immunoreactivity varied with age, as it was most intense at P0, and progressively declined with age. In rats subjected to hypoxia, ET-labeled AMCs were markedly reduced. ET_B_ was present in the early stage of development; at P7, it was present in some AMCs and in ASs; and at P10, it was only detected in ASs. RT-PCR demonstrated ET-1 and ET-3 mRNA, but not ET-2 mRNA	Wu et al. (2006)
TREM_CC_				
CX3CR1 (fractalkine receptor)	C57BL/6 mice (6- to 8-week-old). CX3CR1^−/−^ mice	CPZ diet, ICC, brain flow cytometry, myelin staining, EM, behavior analysis	CX3CR1 is a metabotropic receptor. In CX3CR1-deficient mice, clearance of myelin debris by microglia was blocked, affecting efficient remyelination after a primary demyelinating insult	Lampron et al. (2015)
TREM2 (triggering receptor expressed on myeloid cells 2)	TREM2^−/−^ and wild-type mice (6- to 8-week-old)	CPZ diet, immunoelectron microscopy, RT-PCR, microarray processing, ICC, microglial cell cultures, behavior analysis	TREM2 receptor is a transmembrane protein expressed by several cell types. TREM2 has been implicated in the regulation of phagocytosis and plays a critical role in microglia activation and function during CPZ-induced demyelination. Notably, TREM2^−/−^ mice exhibit defective myelin degradation by microglia	Cantoni et al. (2015)
TREM2	TREM2-genetically deficient (TREM2^−/−^) mice (6- to 8-week-old). CNS tissue from patients with multiple sclerosis aged 39–77 years TREM2^−/−^ mice	CPZ diet, EM, human macrophage cultures, RT-PCR, ICC CPZ diet, EM, ICC	TREM2 directly senses lipid components exposed after myelin damage. Treatment with a TREM2 agonist antibody promoted the clearance of myelin debris in the demyelinated areas. Moreover, TREM2 activation in microglia increased OLG precursor density and the formation of mature OLGs, thus enhancing remyelination and axon integrity TREM2 is a lipid sensor that transmits intracellular signals that promote the overall fitness of microglia during aging and their ability to respond to prolonged demyelination. Indeed, TREM2-deficient mice show defective clearance of damaged myelin by microglia	Cignarella et al. (2020) Poliani et al. (2015)

##### 3.3.4.1 Microglia glutamate receptors in the CC

Like ASs, OLGs and OPCs (see above), microglia respond to Glu. Synaptic Glu, released in the CC (Kukley et al., 2007; Ziskin et al., 2007), may diffuse into the extracellular space and activate both ionotropic and metabotropic Glu receptors on microglial cells (Pocock and Kettenmann, 2007; Table 5). In a study of the distribution of the AMPA receptor subunit Glu_R4_ in neonatal, immature, and mature rat CC (Ong et al., 1996), AMC immunolabeling was scanty in 1- and 2-day-old rats and much more abundant in 7- and 10-day-old rats. In 14-day-old and adult rats, the CC was completely free of labeling. Although the function of iGlu_R4_ on callosal AMCs is unclear, electrophysiological, immunohistochemical, and RT-PCR studies suggest that AMPA receptor activation by Glu enhances production and release of tumor necrosis factor α (TNF-α; Noda et al., 2000). The function of this cytokine is debated, since according to some authors, it potentiates Glu neurotoxicity (Chao and Hu, 1994), whereas according to others, it plays a neuroprotective role in brain responses to excitotoxic injury (Cheng et al., 1994).

##### 3.3.4.2 Microglia notch receptor in the CC

Notch-1 to Notch-4 transmembrane receptors play an important role in the CNS proliferation and differentiation processes occurring during development and are activated following interactions with their Jagged (JAG) and Delta-like family ligands (Grandbarbe et al., 2007) (Table 5). Notch receptor and its ligands affect the migration of neurons to their final destination and promote the differentiation of several GC types. JAG1-Notch-1 signaling regulates the balance between OPC proliferation and maturation in the adult CNS and is involved in OPC differentiation during remyelination (Zhang et al., 2009). Notch-1 is strongly expressed in the developing CC. Notch-1^+^ cells have been identified as AMCs, as they are also labeled with OX-42, a microglial marker. Notch-1 labeling is very intense until P10, then it begins to decline, and becomes even lower at P14. WB analysis has confirmed its age-related downregulation. JAG1 and Delta-1 ligands have been detected on AMCs expressing Notch-1 receptor using immunofluorescence. LPS treatment increased Notch-1^+^ AMC numbers and immunoreactivity in young rats (P3), as confirmed using WB analysis. Moreover, in LPS-treated microglia, RT-PCR showed increased Notch-1 and JAG1 and reduced Delta-1 mRNA levels. The two increases were abolished by dexamethasone, a potent inhibitor of activated microglia. Notch-1 receptor neutralization suppressed mRNA expression of several cytokines like interleukin 1 (IL-1) and IL-6, morphology colony–stimulating factor (M-CSF), and inducible NO synthase (iNOS). Thus, this study suggests that Notch-1 receptor and its ligands regulate AMC expression of iNOS and of various cytokines during postnatal callosal development (Cao et al., 2008).

##### 3.3.4.3 GABA receptors on callosal microglia

The effects of GABA, the principal inhibitory neurotransmitter in the CNS, are mediated through interactions with integral membrane proteins, the GABA receptors (GABA_Rs_) (Table 5). Three classes of GABA_Rs_—GABA_AR_, GABA_BR_, and GABA_CR_—are distinguished by their pharmacological, electrophysiological, and biochemical properties (Chebib and Johnston, 1999). GABA_A_ and GABA_C_ are ionotropic receptors and have integral chloride channels. GABA_B_ is a metabotropic receptor—whose action is linked to the activation of second messenger systems (phospholipase C and adenylate cyclase)—and activates K^+^ and Ca^2+^ ion channels via G-protein coupling. GABA_A_ and GABA_B_ are widely distributed in the CNS, whereas GABA_C_ is predominantly expressed in the retina (Lukasiewicz, 1996). Cell cultures and *in vivo* studies indicate that microglia express different GABA_Rs_, supporting the hypothesis that microglia are cellular targets for GABA (Kuhn et al., 2004; Kettenmann et al., 2011). In the postnatal mouse CC, microglial cells express GABA_ARs_ (Cheung et al., 2009). It has been hypothesized that GABA_ARs_ could be activated by GABA released by the growth cones of developing axons, as proposed for hypothalamic neurons (Gao and van den Pol, 2000). The notion is supported by the presence of transient axon bundles displaying GABA-like immunoreactivity in perinatal rat CC (Cobas et al., 1988). In early postnatal development, GABA plays a variety of roles, promoting cell proliferation and migration, synapse formation and differentiation, and circuit formation (Ben-Ari et al., 2007; Wang and Kriegstein, 2009). It may also do so in the CC (see below and Section 3). In postnatal CC, patch-clamp recording in AMCs treated with the GABA_AR_ agonist muscimol, has revealed a GABA-mediated increase in [K^+^]_o_ via the opening of intrinsic K_ir_ channels, which in turn stimulated the release of macrophage inflammatory protein 1α (MIP1-α). These data suggest that microglia can sense GABA_AR_ activity and that MIP1-α release has the potential to control certain properties of microglia, ASs, and precursor cells in the developing CC (Cheung et al., 2009; Kettenmann et al., 2011). The GABAergic system is also involved in the regulation of callosal myelination through GABA_AR_ activation. This aspect has been studied in late pregnancy, which is characterized by enhanced cellular and ultrastructural remyelination due to an increased GABAergic tone induced by progesterone metabolites. In lysolecithin-induced CC demyelination, pregnancy-associated remyelination was impaired when GABA_ARs_ were blocked by bicuculline (a competitive GABA_AR_ antagonist), affecting negatively the intrinsic phagocytic action of microglia (Kalakh and Mouihate, 2019). Although detailed analysis has indicated that GABA_BRs_ are widely expressed throughout the CNS, their expression in callosal microglia is yet to be explored.

##### 3.3.4.4 Purinergic receptors on microglial callosal cells

As noted above, ATP and its receptors are involved in glia–glia communication mediated by the onset and propagation of Ca^2+^ waves (Table 5). Furthermore, the activation of purinergic receptors induces activation of a K^+^ current and cytokine secretion (Fields and Burnstock, 2006; Kettenmann et al., 2011; Gundersen et al., 2015). Similar results have been described in callosal microglial cells. Electrical stimulation or local ATP ejection in acute brain slices triggered Ca^2+^ waves in the CC. The waves spread over hundreds of micrometers and activated Ca^2+^ responses in both callosal ASs and microglia, which were identified by labeling with Texas Red–conjugated tomato lectin. Recordings from dye-coupled lectin-labeled cells showed the transient induction of an outward K^+^ rectifying current upon the passage of the Ca^2+^ wave, a characteristic response of purinergic receptors (Schipke et al., 2002). Studies of 5- to 7-day-old mice have indicated that callosal microglia express several purinoreceptor subtypes that control various membrane conductance parameters. Extracellular application of 1 mM ATP evoked generation of a complex membrane current with three components: i) a fast inward current due to the activation of non-specific cationic channels, ii) a small steady-state inward current carried by K^+^ ions, and iii) a delayed inward current associated with the activation of non-selective channels. The first two components had a low activation threshold (10 μM ATP) and were significantly reduced by suramin, a P_2_ purinoreceptor antagonist, whereas the third component was only observed by applying 1 mM ATP. Notably, the addition of the tetra-anionic form of ATP (ATP^4−^) to the bath solution lowered the threshold of the third component, suggesting that ATP^4−^ is its true agonist (see above) which preferentially activates P_2Z_. These data seem to indicate that the first two components are mediated by P_2X_ purinoceptors, whereas the third is due to the involvement of P_2Z_ (Haas et al., 1996; Kettenmann et al., 2011). Studies of CC slices of 6- to 9-day-old rats have confirmed the presence of P_2_ functional receptors on callosal GCs. The application of 200 mM ATP for 30 s caused a transient increase in [Ca^2+^]_i_ in 25% of the microglial cells that had migrated to the top of the slices (Möeller et al., 2000). By contrast, adenosine failed to increase [Ca^2+^]_i_, suggesting that the changes in [Ca^2+^]_i_ are due to ATP interaction with P_2X_ receptors. [Ca^2+^]_i_ transients were also recorded after application of thapsigargin, a specific blocker of the ER Ca^2+^ pump, confirming the existence of Ca^2+^-permeable P_2X_ receptors on a population of callosal GCs. In addition to the presence of P_2X_ receptor, this study revealed the existence of P_2Y_ receptor. After removing extracellular Ca^2+^ from the bath solution and applying ATP, it was still possible to record a transient [Ca^2+^]_i_ that was reduced and/or inhibited by thapsigargin. These data suggest the presence of metabotropic P_2Y_ purinergic receptors capable of mobilizing Ca^2+^ from the ER (Möeller et al., 2000). Several purinergic receptors have recently been demonstrated in callosal AMCs in normal and hypoxic rats. P_2X4_, P_2X7_, and P_2X12_ have been detected in the CC from P0 to P7 using immunohistology; although all three receptors were detected in callosal AMCs, P_2x4_ was the most intensely expressed and progressively diminished with age. Notably, P_2X4_ expression was increased in hypoxic P0 rats, and IHC and WB analysis showed P_2X4_ upregulation at 4–72 h and P7 after hypoxia. In addition, hypoxic treatment caused similar increases in cytokine expression and release in AMCs, while WB analysis showed upregulation of IL-1β and TNF-α protein expression that was completely blocked by 2′,3′-0-(2,4,6-trinitrophenyl) adenosine 5′-triphosphate, the selective P_2X4_ receptor blocker. All these data suggest that after hypoxia, P_2X4_ receptor mediates ATP-induced callosal AMC activation and their production of proinflammatory cytokines (Li et al., 2011).

##### 3.3.4.5 Microglia expressing fractalkine receptors and triggering receptors expressed on myeloid cells

Clearance of myelin debris in the CC depends on the presence of at least two microglial receptors: i) fractalkine receptor CX3C_R1_ and ii) triggering receptor expressed on myeloid cells 2 (Table 5). Proper remyelination requires CX3C_R1_. In CX3C_R1_ knockout mice, impaired myelin phagocytosis by microglia leaves myelin debris in the CC and adversely affects remyelination (Lampron et al., 2015). Triggering receptor expressed on myeloid cells 2 (TREM2) belongs to the TREM family of extracellular innate immunoreceptors (Colonna and Wang, 2016). The extracellular region of TREM2 recognizes lipid components from callosal myelin damage (Cantoni et al., 2015; Cignarella et al., 2020). Microglial TREM2 is therefore considered to be a lipid sensor capable of binding, engulfing, and clearing myelin debris and may also contribute to microglia lipid metabolism (Cantoni et al., 2015; Poliani et al., 2015; Cignarella et al., 2020). CPZ (a demyelinating agent; Matsushima and Morell, 2001; Vega-Riquer et al., 2019)–treated TREM2-deficient (*Trem2* ^*−/−*^) mice show reduced microglia proliferation and activation, extensive myelin debris accumulation due to defective clearance in callosal areas undergoing demyelination, and defective lipid degradation inside cells (Cantoni et al., 2015; Poliani et al., 2015).

##### 3.3.4.6 Endothelin and endothelin receptor expression in the CC

Endothelin (ET), a potent EC-derived vasoconstrictor peptide, was first isolated from pig aorta ECs and named ET-1 (Yanagisawa et al., 1988; Ortega Mateo and De Artiñano, 1997). Two other isopeptides, ET-2 and ET-3, were later identified in humans (Inoue et al., 1989; Ortega Mateo and De Artiñano, 1997) (Table 5). Two mammalian ET_Rs_, ET_A_ and ET_B_, with different degrees of affinity for the three isopeptides, were subsequently isolated (Davenport et al., 2016; Houde et al., 2016). Although ET_B_ binds with equal affinity to all three ET isopeptides, ET-3 possesses very low affinity to ET_A_ (Davenport et al., 2016; Gulati, 2016; Houde et al., 2016). Both ETs and ET_Rs_ have been identified in most areas of the CNS of both humans and rodents (Jones et al., 1989; Lee et al., 1990; Niwa et al., 1991; Gulati and Srimal, 1992; McLarnon et al., 1999). They are expressed by neurons (Lee et al., 1990; Gulati and Srimal, 1992), ASs (MacCumber et al., 1990), microglia (McLarnon et al., 1999), and ECs (MacCumber et al., 1990). ET and ET_R_ expressions have been detected using ICC in rat CC during postnatal development (Wu et al., 2006). The intensity of ET immunoreactivity and the number of positive cells varied with age. The most numerous and most intensely labeled cells were observed at P0. ET expression then plummeted at P10, and no labeled cells were detected at P14 (Wu et al., 2006). Double-labeling experiments with isolectin B4, a microglial cell marker, showed that ET^+^ cells were AMCs. In rats subjected to hypoxia, the number of ET-labeled AMCs diminished considerably at each time point. In the early postnatal period, ET_B_ was detected in rat AMCs; at P7, it was observed in some AMCs and in ASs, whereas at P10, it was localized only in ASs. ETA has never been described in AMCs (Wu et al., 2006). Therefore, the morphological and functional changes of AMCs to ramified microglia determine a progressive reduction of ETs and of cytokine/chemokine synthesis. The presence of AMC-derived ETs in postnatal CC suggests that they may influence AMC and glial precursor proliferation, differentiation, and migration through chemokine synthesis and secretion. Moreover, the presence of ET_B_ in AMCs indicates that ET release can also act via an autocrine mechanism (Wu et al., 2006).

#### 3.3.5 Microglia in the nodes of Ranvier of CFs

The myelin sheath is periodically interrupted by NoRs, which expose portions of the axon membrane (Debanne et al., 2011). These very small areas contain a heterogeneous population of GCs, i.e., OPCs (NG2), ASs (see above), and microglia (Zhang et al., 2019; Ronzano et al., 2021). A study using double and triple immunofluorescence staining and EM (NoR, myelin sheath, and microglia) has demonstrated that microglial processes contact the axolemma at NoRs and are closely apposed to myelin paranodal regions, with a gap of 5–10 nm (Zhang et al., 2019). The NoRs are involved in impulse propagation (saltatory conduction), which is ensured by voltage-gated Na^+^ channels, Ca^+^-activated K^+^ channels, and the justaparanodal expression of voltage-gated K^+^ channels (Debanne et al., 2011; Gründemann and Clark, 2015). Microglia express different types of K^+^ channels (Dolga and Culmsee, 2012), in particular callosal microglia express inward rectifier K^+^ channels (Brockhaus et al., 1993) and two-pore domain (THIK-1) channels, the main CNS channels involved in microglial K^+^ conductance (Madry et al., 2018; Ronzano et al., 2021). The role of microglia at the NoRs remains elusive. It has been hypothesized that in this position, callosal microglia can monitor and resorb extracellular K^+^ released during neural activity (Zhang et al., 2019). Indeed, it has been reported that microglial contacts at NoRs are modulated by neuronal activity and that microglia–neuron communication depends on the K^+^ level at the node area, with an important role played by THIK-1 channels (Ronzano et al., 2021). K^+^ flows in the NoRs also influence remyelination. In slices of demyelinated nervous tissue, the application of tetraethylammonium and tetrapentylammonium to inhibit K^+^ channels and the microglial THIK-1 channel, respectively, drastically reduced the remyelination rate, altered microglia-NoR interaction, and impaired the microglia switch toward a proregenerative phenotype (Ronzano et al., 2021).

## 4 Conclusion

The release of neuroactive substances such as Glu, adenosine, and probably, NO in the CC; the presence of different types of receptors and vesicular transporters on CFs; the ICNs connecting different CNS regions; the different types of GCs and their receptors and transporters; and the crosstalk among all these elements make the CC a probable site of signal processing rather than one where messages are passively relayed from one hemisphere to the other.

## Figures and Tables

**Table:** 

**ACh**	Acetylcholine
**AChE**	Acetylcholinesterase
**ADP**	Adenosine diphosphate
**AMCs**	Ameboid microglial cells
**AMPA**_**Rs**_	α-Amino-3-hydroxy-5-methyl-4-isoxazolepropionic acid receptor
**Ang II**	Angiotensin II
**APs**	Action potentials
**AQP-4**	Aquaporin-4
**A**_**R**_	Adenosine receptors
**Asp**	Aspartate
**ASs**	Astrocytes
**ATP**	Adenosine triphosphate
**BBB**	Blood–brain barrier
**BDNF**	Brain-derived neurotrophic factor
**BOLD**	Blood oxygen-level–dependent signal
**CalB**	Calbindin
**CalR**	Calretinin
**CAPs**	Compound action potentials
**Ca**_**Vs**_	Voltage-gated Ca^2+^ channels
**CAxs**	Callosal axons
**CBF**	Cerebral blood flow
**CC**	Corpus callosum
**CFs**	Callosal fibers
**cGMP**	Guanosine 3′-5′-cyclic monophosphate
**CNS**	Central nervous system
**CNPase**	2′,3′-cyclic nucleotide 3′-phosphodiesterase
**CPA**	Cyclopentyladenosine
**CPu**	Caudate putamen (striatum)
**CPZ**	Cuprizone
**CTB**	Cholera toxin subunit B
**CSF**	Cerebrospinal fluid
**cADPR**	Cyclic adenosine diphosphate ribose
**DAG**	Diacylglycerol
**DiI**	1, 1′-Dioctadecyl-3,3,3′,3′- tetramethylindocarbocyanine perchlorate
**D**_**Rs**_	Dopamine receptors
**DTI**	Diffusion tensor imaging
**DTT**	Diffusion tensor tractography
**ECs**	Endothelial cells
**EGFP**	Enhanced green fluorescence protein
**EM**	Electron microscopy
**ET**	Endothelin
**ET**_**Rs**_	Endothelin receptors
**GABA**	γ-Amino butyric acid
**fMRI**	Functional magnetic resonance imaging
**GABA**_**Rs**_	GABA receptors
**GCs**	Glial cells
**GFAP**	Glial fibrillary acidic protein
**GLAST**	Glutamate aspartate transporter
**Glu**	Glutamate
**Glu**_**Rs**_	Glutamate receptors
**Glu**_**Ts**_	Glutamate transporters
**GM**	Gray matter
**Hi**	Histochemistry
**HRP**	Horse radish peroxidase
**ICFs**	Intracallosal fibres
**ICNs**	Intracallosal neurons
**IG**	Indusium griseum
**IGF-1**	Insulin-like growth factor 1
**IHC**	Immunohistochemistry
**IL**	Interleukin
**IP**_**3**_	Inositol trisphosphate
**I-OLGs**	Interfascicular oligodendrocytes
**LM**	Light microscopy
**LPS**	Lipopolysaccharide
**LVs**	Lateral ventricles
**MAP**	Microtubule-associated proteins
**MBP**	Myelin basic protein
**MCT**	Monocarboxylate transporter
**mGlu**_**Rs**_	Metabotropic glutamate receptors
**MIP1-α**	Macrophage inflammatory protein 1α
**MRI**	Magnetic resonance imaging
**NADPHd**	Nicotinamide adenine dinucleotide phosphate diaphorase
**NADPHd Ns**	Nicotinamide adenine dinucleotide phosphate diaphorase neurons
**NeuN**	Neuronal nuclear antigen
**NG2**	Cells expressing chondroitin sulfate proteoglycan
**NK1**_**R**_	Neurokinin 1 receptor
**NMDA**_**Rs**_	*N*-methyl-D-aspartate receptor
**NO**	Nitric oxide
**nNOS**	Neuronal nitric oxide synthase
**nNOS Ns**	Neuronal nitric oxide synthase neurons
**NPY**	Neuropeptide Y
**NSE**	Neuron-specific enolase
**OLGs**	Oligodendrocytes
**OPCs**	Oligodendrocyte precursor cells
**PG**	Prostaglandin
**PLP**	Proteolipid protein
**SP**	Substance P
**TLD**	Laterodorsal tegmental nucleus
**TNF-α**	Tumor necrosis factor
**UFs**	Unmyelinated fibers
**vGlu**_**Ts**_	Vesicular glutamate transporters
**WB**	Western blot
**WGA**	wheat germ agglutinin
**WM**	White matter
